# The molecular mechanism of constructive remodeling of a mechanically-loaded polymer

**DOI:** 10.1038/s41467-022-30947-8

**Published:** 2022-06-07

**Authors:** Chenxu Wang, Sergey Akbulatov, Qihan Chen, Yancong Tian, Cai-Li Sun, Marc Couty, Roman Boulatov

**Affiliations:** 1 Department of Chemistry, University of Liverpool, Liverpool, L69 7ZD UK; 2 Manufacture Française des Pneumatiques Michelin, Clermont-Ferrand, 63000 France

**Keywords:** Polymers, Reaction mechanisms, Polymer characterization

## Abstract

Large or repeated mechanical loads usually degrade polymers by accelerating fragmentation of their backbones but rarely, they can cause new backbone bonds to form. When these new bonds form faster than the original bonds break, mechanical degradation may be arrested or reversed in real time. Exploiting such constructive remodeling has proven challenging because we lack an understanding of the competition between bond-forming and bond-breaking reactions in mechanically-stressed polymers. Here we report the molecular mechanism and analysis of constructive remodeling driven by the macroradical products of mechanochemical fragmentation of a hydrocarbon backbone. By studying the changing compositions of a random copolymer of styrene and butadiene sheared at 10 °C in the presence of different additives we developed an approach to characterizing this growth/fracture competition, which is generalizable to other underlying chemistries. Our results demonstrate that constructive remodeling is achievable under practically relevant conditions, requires neither complex chemistries, elaborate macromolecular architectures or free monomers, and is amenable to detailed mechanistic interrogation and simulation. These findings constitute a quantitative framework for systematic studies of polymers capable of autonomously counteracting mechanical degradation at the molecular level.

## Abstract (web summary)

Large mechanical loads usually degrade polymers by accelerating fragmentation of their backbones but rarely, they can cause new backbone bonds to form leading to arrested or reversed degradation when new bonds are formed faster than the original bonds break.Here, the authors report the molecular mechanism and analysis of constructive remodeling driven by the macroradical products of mechanochemical fragmentation of a hydrocarbon backbone.

## Introduction

Mechanical load accelerates fragmentations of polymer chains, degrading its properties^1–6^. A popular chemical approach to reversing this damage is to control the products of such fragmentations by incorporating mechanochemically labile bonds into common polymer backbones^7–13^. Once the load is removed, bimolecular additions of pairs of the resulting fragments, usually upon energy input in the form of heat, irradiation or coreactants, regenerates many (but not all^14^) failed relatively labile backbone bonds. Despite numerous impressively clever proof-of-the-concept demonstrations, practical implementations of this approach face considerable barriers. Incorporating dissociatively labile bonds into polymer backbones necessarily affects the mechanical properties (which may be partially ameliorated by including such bonds into chain loops^15,16^) and processibility of the polymer^5^, increases costs and complicates synthesis.

The alternative to healing the damage after it occurred is to form new backbone bonds in the loaded material fast enough for the size of the average chain to remain constant or grow despite simultaneous competing mechanochemical chain fracture. Such constructive remodeling^9,17,18^ is a potentially effective approach to autonomously reinforcing overstressed volumes of the loaded material that are at the highest risk of failure. Its only known demonstration relied on forming new bonds away from the sites of chain fracture in a sheared mixture of a polymer of dihalocyclopropane (DHC) and a small-molecule bifunctional crosslinker^18^. Mechanical load accelerated isomerization of DHC monomers to allylic halides that combine spontaneously with one terminal group of the small-molecule crosslinker. Unlike the covalent rebonding approach above, no mechanically fractured bond was regenerated, and the new bonds formed irreversibly. The resulting crosslinking kinetics is largely uncontrollable (although a partial work-around was demonstrated in solution^19^): at the onset of the remodeling, the concentration of isomerized monomers is high and new bonds form much faster than mechanochemical bond scission, causing embrittlement. As either reactant is depleted, crosslinking can no longer compensate bond fracture and the material degrades as usual.

The above analysis suggests that enabling spontaneous backbone bond formation to outcompete mechanochemical bond scission is a necessary but insufficient requirement to realize practical constructive remodeling. We identify at least 4 additional requirements. First, it doesn’t require mechanochemically labile backbone bonds. Second, it generates backbone bonds that are as chemically indistinguishable from the lost bonds as possible, to avoid the problem of reactive-group depletion. Third, it forms new bonds both between products of chain fracture and between intact chains, to rapidly grow the average chain (or increase the crosslinking density) in overstressed volumes. Fourth, the number of new bonds formed per fractured bond is independent of the degree of remodeling and tunable by changes in the polymer composition and/or microstructure to yield stable remodeling behavior adjustable to the anticipated loading profiles the material will experience during use.

The above considerations suggests that the abundance of simple C–C bonds in backbones of synthetic polymers^20^ makes spontaneous regeneration of simple C–C backbones in a mechanically stressed polymer a particularly attractive target for constructive remodeling. When overstretched, a C–C backbone bond usually homolyzes to a pair of macroradicals^1,2,21^. Such mechanochemically generated macroradicals were previously used as macroinitiators of radical polymerization of styrene or acrylates^9,22–24^, which forms new backbone bonds. Whereas swelling polymers with a monomer or decorating them with pendant acrylate or styrene groups is impractical, a number of polymers already in commercial use, exemplified by polybutadiene, have either backbone or pendant olefin C=C bonds. In theory, such polymers could serve as a starting point for integrating constructive remodeling into existing and emerging engineering materials. In practice, we lack an understanding of the factors that control the rates of spontaneous regeneration of simple C–C backbones under mechanical load or the tools to enumerate these factors. The present study is a first step to filling this knowledge gap.

Unlike the more reactive C=C bonds of acrylates or styrenes, few examples of uncatalyzed condensed-phase radical polymerizations of unconjugated C=C have been reported, or studied mechanistically^25^. The reaction is slow and only short kinetic chain lengths have been achieved in solids even at >100 °C^26^. Heating certain polybutadienes or styrene/butadiene copolymers in the presence of an organic peroxide creates up to 20 crosslinks per peroxide molecule, presumably by addition of chemically-generated macroradicals to the vinyl groups of the adjacent chains^27^. However, other unsaturated polymers, including polyisoprene, neoprene and many butadiene copolymers only produce (sub)stoichiometric amounts of crosslinks. Crosslinking by addition of a macroradical to a backbone C=C bond was also proposed in unsaturated polyolefins at >60 °C^28,29^ but the number of new bonds per macroradical is unknown and likely substoichoimetric. Conversely, macroradical recombinations, not additions to sp^2^ C atoms, are thought to dominate polymer crosslinking in reactive melt processing^26,30^ and in polyolefins under γ irradiation. Finally, in ball milling of unsaturated polyolefins, such as polyisoprene, or their mixtures with saturated polymers the new bond formation, evident by chain grafting, is always accompanied by a reduction in the average chain size, suggesting that chain fracture dominates^21,23^.

Although these examples suggest that olefinic C=C bonds may support spontaneous formation of new backbone C–C bonds without the need for free monomer, they tell us little about whether such bond formation can outcompete bond loss by chain fracture, which is the minimum requirement of constructive remodeling. Biasing this competition towards bond formation is more challenging in a mechanically-stressed than in a heated polymer because the susceptibility of a chain to fracture mechanochemically scales much more steeply with chain size^2^ and the degree of branching^31^ than thermal chain scission. In other words, the large and topologically complex products of addition of macroradicals to surrounding chains are proportionally more likely to fracture when overstretched than heated. No existing knowledge allows the effect of the resulting mechanochemical feedback loops on the remodeling kinetics to be estimated even qualitatively.

Molecular mechanisms of mechanochemical remodeling of bulk polymers have been little studied, which means that the field lacks consensus protocols that govern the design of such studies or interpretations of their results. Here we adopted a physical organic approach^32^, combining experiments, quantum-chemical calculations and mechanistic microkinetic simulations, to enumerate and validate the simplest mechanism responsible for changes in the composition and microstructure of random styrene/butadiene copolymer under steady-state shear. Our results demonstrate that constructive remodeling based on radical oligomerization of low-reactivity unconjugated C=C bonds, initiated by mechanochemically generated macroradicals, is both feasible under practically relevant conditions and mechanistically tractable at the molecular level.

## Results

**Fig. 1:**
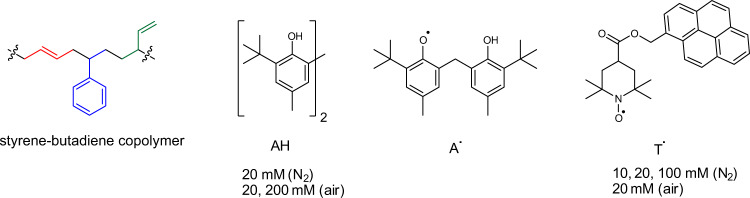
The styrene-butadiene copolymer and radical scavengers. The sheared polymer had *T*_g_ = −35 °C and contained 30.5 ± 0.5% and 27.1 ± 0.7% by mass of styrene (blue) and 1,2-enchained butadiene (green), respectively, with the rest being 1,4-enchained butadiene (red), as determined by NIR spectroscopy (Supplementary Fig. 1). AH represents a class of antioxidant additives routinely used for protection of commercial polymers against thermooxidative aging^38,39^. A^•^ is a radical scavenger produced in situ when a macroradical abstracts phenolic hydrogen atom from AH. T^•^ combines a well-established radical trap^40^ (TEMPO) with pyrene, whose characteristic absorption spectrum allows simple and accurate spectroscopic quantitation of the number of T moieties bound to a polymer chain. Bulk concentrations of AH and T^•^ used in a subset of our experiments are also listed.

We chose to study the mechanism of regeneration of C–C backbones in sheared random copolymer of butadiene and styrene (Fig. 1) because of its commercial importance (its annual production is worth $13b), the mapped relationship between its mechanical properties and its composition^33^, and synthetic accessibility of copolymers with different ratios of the three repeat units^34^. These attributes allowed us to select the composition that maximized the achievable level of mechanistic characterization of its remodeling. Our current study did not aim at enumerating or understanding physical processes that accompany remodeling, including the rheological behavior^35^ and how the applied stress is transduced into overstretched chains^36^ because the challenge of molecular interpretation of such processes^37^ reduces their utility in mechanistic studies.

## Experimental results

All experimental results discussed here are for samples of identical mass distribution (*M*_n_ = 151 ± 5 kDa, *M*_w_ = 165 ± 2 kDa) sheared at 10 °C to minimize thermal remodeling; see Supplementary Fig. 7 and accompanying text for a brief discussion of the results at other temperatures and for other initial mass distributions. Each sample was repeatedly passed through a 1 × 10 mm (dia. × length) capillary at a constant velocity and frequency in either nitrogen atmosphere or dry air (see Methods and Supplementary Information for further details). We continuously recorded the applied force (Supplementary Fig. 6) and periodically sampled the material for analysis by size-exclusion chromatography (SEC) and spectrophotometry. In addition to neat copolymer, we sheared samples containing a dissolved radical scavenger, T^•^ (at 10, 20 and 100 mM for anaerobic experiments and 20 mM for aerobic shearing, Fig. 1) and a common antioxidant additive, AH (at 20 mM for anaerobic and 20 and 200 mM for aerobic experiments). T-containing samples allowed us to understand the relative contributions of chains of different mass and topology to remodeling kinetics. We quantified the effects of O_2_ and the antioxidant (AH) on the remodeling mechanism because constructive remodeling needs to operate in the presence of either solute to be practical. In our experiments the concentration of AH was up to 20 times greater than that used in commercial polymers to maximize its mechanistic effects. Because chain/chain additions produce complex mixtures of chains of different topology, the true mass distribution of a sheared material is accessible only from simulations^41^ as described below. Here all reported measured molar masses are apparent: for linear chains, the true and apparent masses are identical, whereas branched chains of the same hydrodynamic radius have the same apparent mass irrespective of their microstructures and masses.

**Fig. 2:**
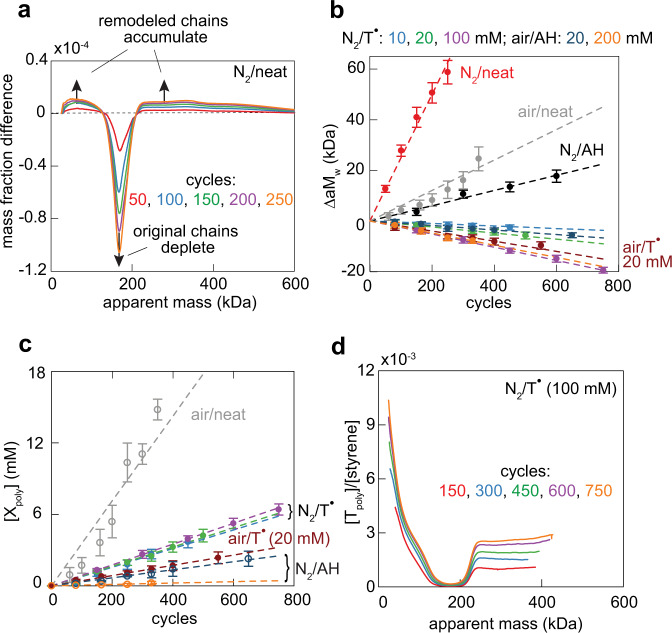
Summary of experimental results. **a** Changes in the mass fraction of chains of each apparent mass relative to that in the initial sample of neat copolymer sheared under N_2_ (see Supplementary Figs. 10, 11 for all absolute and differential aMMDs and Supplementary Figs. 12–14 for corresponding SECs). **b** Changes of apparent *M*_w_ of sheared samples. **c** The concentration of polymer-bound T or OOH moieties, [X_poly_], (filled and open circles, respectively), as a function of the number of shearing cycles. Note that for aerobically sheared T-containing sample, only [T_poly_] was measured. Data sets not individually labelled in (**b,** **c**) are identified in the legend at the top of (**b**). The error bars define the 2σ confidence intervals. **d** The number of polymer-bound T moieties per styrene, [T_poly_]/[styrene], for chains of each apparent mass in anaerobically sheared copolymer containing 100 mM T^•^ (see Supplementary Fig. 16 for the other samples). The low [T_poly_]/[styrene] ratios at 160–200 kDa reflect the dominance of intact chains at these masses. All data illustrated here and the SI is provided in the Supplementary Data file (see data availability statement).

Apparent molar mass distributions (aMMDs) of sheared samples demonstrate accumulation of chains with masses both smaller and larger that the initial chains under all studied conditions (Fig. 2a and Supplementary Fig. 10), suggesting that chains both fracture and the resulting macroradicals add to surrounding chains. In all samples the distribution of the low-mass product fraction was centered at the mass approximately half that of the initial sample, as is commonly observed in bulk samples under diverse loads^1,2^. Compared to anaerobically sheared neat copolymer, changes in the apparent weight-average mass, a*M*_w_ of all other sheared samples (Fig. 2b) along with their bifurcated aMMDs (Supplementary Fig. 10) suggest that O_2_, T^•^, and AH suppress but do not eliminate the C–C bond regeneration even at the highest concentrations employed (0.1 and 0.2 M for T^•^ and AH, respectively).

Transient generation of macroradicals in aerobically-sheared samples is suggested by the accumulation of chain-bound hydroperoxy (OOH) groups, whose concentrations we quantified by iodometry^42,43^. Conversely, hydroxy or carbonyl groups which accumulate in aerobically heated polyolefins^44^ by thermal decomposition of hydroperoxidized chains were undetectable. AH reduced chain peroxidation ~10-fold at 20 mM and 60-fold at 200 mM. We could not quantify the concentration of OOH moieties in aerobically-sheared T-containing samples because of the interference from residual T^•^.

Shearing samples containing dissolved T^•^ incorporated T moieties into polymer chains, as evidenced by the appearance of the pyrene absorption in UV-vis spectra of SEC fractions primarily at apparent masses smaller and larger than those of the intact sample. By deconvoluting the spectrum of each fraction into that of styrene and pyrene (Supplementary Fig. 15), we estimated the average number of T moieties per styrene for all chains of the same apparent mass. The total concentration of these moieties, [T_poly_], scaled linearly with the number of shearing cycles. In contrast to the accelerated loss of a*M*_w_ in samples with higher bulk T concentrations, [T]_bulk_, the accumulation rate of [T_poly_] was independent of [T]_bulk_, but slowed by O_2_ (Fig. 2c: green, magenta and blue lines vs. brown line). This suggests that under N_2_, T^•^ binding is the dominant sink of macroradicals at all [T]_bulk_ studied, whereas O_2_ enables additional radical-destroying reactions. Negligible addition of macroradicals to pyrene of T^•^ is evidenced by the total pyrene absorption of sheared samples remaining constant throughout shearing.

### The remodeling mechanism from DFT computations

**Fig. 3:**
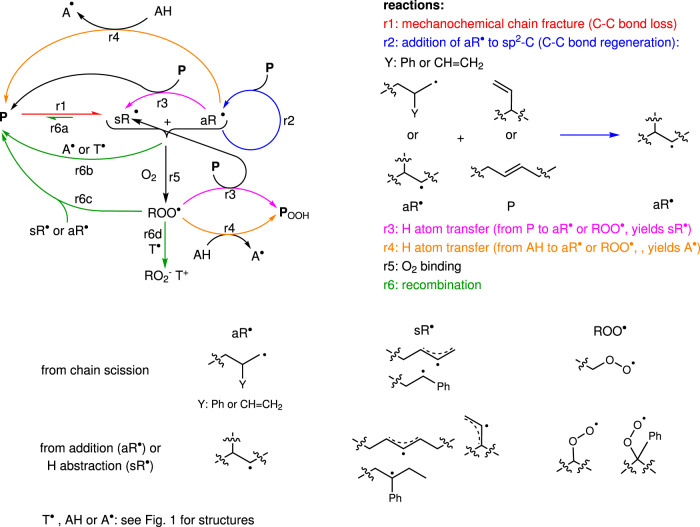
The simplest mechanism of low-temperature mechanochemical remodeling of neat and doped copolymer under N_2_ and air. P is any closed-shell copolymer chain regardless of microstructure, size or functional groups.

Fragmentation of a polymer chain produces an intractable mixture of structurally distinct macroradicals. Reactions of these macroradicals with other chains or small-molecule solutes further increase the number of species. To reduce this complexity, we calculated the reaction and activation enthalpies, Δ*H*° and Δ*H*^‡^, respectively, of H-atom transfer and addition to sp^2^-C atoms for representative pairwise combinations of short copolymer and macroradical segments (Supplementary Tables 4–7). The results confirmed that the reactivities of all macroradicals plausibly formed in mechanically loaded copolymer can be represented by two species: aR^•^ that includes all alkyl radicals and sR^•^ (for stabilized radicals) that includes all allylic and benzylic radicals (Fig. 3). The results were broadly consistent across the 6 functionals we tested (Supplementary Table 3), with the numbers cited throughout the main paper are at the uMPW1K/6–31 + G(d) level. Calculated Δ*H*° and Δ*H*^‡^ for either addition or abstraction vary by <2 kcal/mol among radicals of the same type regardless of their size, branching, or the internal vs. terminal location of the unpaired electron. All calculated Δ*H*° and Δ*H*^‡^ of aR^•^ reactions are less than those for equivalent sR^•^ reactions by >15 and ~5 kcal/mol, respectively (Supplementary Tables 3, 4), consistent with the known differences between alkyl and stabilized (allylic/benzylic) radicals^45^.

The fraction of aR^•^ in the fragmentation products of the copolymer was calculated to increase monotonically with fracture force before plateauing at ~50% for ≥4.2 nN (Supplementary Fig. 20a). The chain fracture force(s) in a sheared material are unknown. However, they must be high enough to reduce the half-life of the overstretched backbone to (much) less than the residence time of the chain in the capillary (1.6 s) and its terminal relaxation time (<10 s, see SI for further details), because otherwise, the chain will relax before fracturing. At 10 °C and τ_½_ < 1.6 s, the average chain fracture produces 0.8–0.98 aR^•^ on average.

Based on these results we identified 4 generic reactions (r1-r3 and r6a, Fig. 3) that constitute the simplest mechanism of mechanochemical remodeling of neat copolymer under N_2_. Mechanochemical chain scission (r1, red arrow) yields a pair of shorter macroradicals, sR^•^ and aR^•^, thus reducing the average chain size and the number of backbone bonds. A new backbone C–C bond is formed whenever aR^•^ adds to a random non-aromatic sp^2^ C (either backbone or pendant) of an adjacent chain (P), yielding a larger and more branched alkyl radical, aR^•^ (r2, blue arrow). The resulting aR^•^ continues to accrete P chains sequentially until it abstracts an allylic or benzylic H atom from an adjacent chain (r3, magenta arrow), creating internal sR^•^ and itself becoming P, or when it recombines with another aR^•^ or sR^•^ (r6a, green arrow). Addition of sR^•^ to an sp^2^-C atom requires traversing considerably higher activation barrier than that of aR^•^, so that in the absence of an exogenous reactant, the main sink of sR^•^ is macroradical recombinations (r6a, green line).

The presence of A^•^, T^•^ or O_2_ enables additional reactions. Our calculations suggest, in accord with previous experimental^40,46^ and computational^47,48^ data, that all macroradicals bind A^•^, T^•^ or O_2_ at diffusion-limited rates (Supplementary Table 14), which are determined both by the molecular size of A^•^, T^•^ or O_2_ (Supplementary Table 14) and their concentrations. Binding of T^•^ or A^•^ to a macroradical (r6b) yields closed-shell products whose reactivity is qualitatively identical to that of the initial chain, P. These reactions compete with additions of aR^•^ to P (r2) and with recombinations of macroradical pairs, reducing the average size of remodeled P, which is indeed observed. Another reaction that can terminate aR^•^ growth is H atom transfer from AH (r4, orange), which is the primary source of A^•^ in the absence of O_2_.

Diffusion-limited binding of O_2_ to sR^•^ or aR^•^ yields peroxy radicals, ROO^•^ (r5)^49^, whose reactivity is, according to our calculations, independent of the nature of R (Supplementary Table 5) and distinct from that of C-based radicals in at least three important aspects. First, ROO^•^ has no affinity for O_2_ or A^•^ and is rapidly reduced by T^•^^50^. Second, unlike aR^•^, ROO^•^ kinetically favors H atom abstraction (r3) over addition to sp^2^-C atoms (r2). Third, the relatively slow addition of ROO^•^ to sp^2^ C yields a radical that undergoes spontaneous irreversible O–O bond homolysis to regenerate chains of the original size and microstructure faster than the addition rate (Supplementary Tables 5, 10, 11, and ref. ^49^). Consequently, addition of ROO^•^ to sp^2^ C atoms is expected to have no observable manifestations and is omitted from our mechanism. In the absence of AH or T^•^, ROO^•^ is primarily depleted by H abstraction from an adjacent chain (r3). This reaction generates internal sR^•^ and a closed-shell chain containing a hydroperoxide moiety (P_OOH_), whose reactivity at the experimental temperature is indistinguishable from that of any other closed-shell chain of the copolymer. Recombination of ROO^•^ with aR^•^ or sR^•^ (r6c), generating a larger chain with an O–O backbone is of secondary importance as explained in the next section. Finally, in the presence of AH, diffusion-limited rate of H transfer from AH to ROO^•^ (r4, Supplementary Table 11) further increases the fraction of ROO^•^ that becomes stable P_OOH_.

The relative simplicity of the mechanism in Fig. 3 is due, in part, to the low temperature of our experiments, which considerably reduces the range of reactions that are kinetically competent to contribute to remodeling. For example, diverse reactions initiated by thermal decomposition of peroxidized polymers at >60 °C, which are thought to be responsible for self-accelerating degradation of polyolefins heated in air^51^, are made irrelevant by the ~10^4^-year half-life of alkyl hydroperoxides at 10 °C^52,53^. The same applies to β-scissions of macroradicals, which are important in anaerobic pyrolysis^51^ and reactive melt processing of polyolefins^26,30^ (Supplementary Table 11). Other reactions, such as intramolecular additions of certain macroradicals and intramolecular H migration (Supplementary Table 8) may be kinetically competitive with reactions in Fig. 3 but because they do not change the chain size or interconvert aR^•^ and sR^•^ macroradicals, they do not affect the observable properties of the remodeling polymer, making their inclusion in the remodeling mechanism unsupportable by the available empirical data.

### Mechanistic microkinetic simulations

We confirmed that the mechanism in Fig. 3 reproduces quantitatively all available experimental data using kinetic parameters whose values are close to those calculated by DFT. To do so we simulated the evolution of both the composition and topology of each polymer chain comprising the remodeling sample, which is more informative than fitting a putative mechanism to either the observed apparent MMD or the bulk composition only, as has been standard in the field. For example, apparent time-dependent MMDs of a polymer that undergoes competing non-mechanochemical chain fracture and crosslinking were simulated using a simple empirical mathematical expression without defining the underlying reaction mechanism^54^. Such an approach obviously cannot demonstrate that a mechanism reproduces the experiments^51^. Conversely, the reported mechanistic simulations attempted to reproduce only the total concentrations of certain functional groups while ignoring how these functional groups were distributed among chains of different masses or/and to simulate only apparent time-dependent *M*_w_ or *M*_n_ rather than the full MMD. This alternative is equally unsuitable for a mechanochemically remodeling polymer because chains of different size and topology are characterized by vastly different susceptibilities to fracture when overstretched. The other advantage of our simulations is the detailed description of the distribution of chain sizes and topologies in the remodeling samples that it yields.

To make keeping track of tens of thousands of chains of unique mass and topology that comprise a remodeling polymer tenable, we exploited simple relationships between the size and contour length of each chain and its reaction probabilities to define the tens of thousands of microscopic rate constants that describe the kinetics of each unique chain using just 12 independent kinetic parameters. First, we relied on a previously established finding^1,2^ that the fragmentation rate constant of a chain scales as power, *m*, of its contour length (or spanning lengths for branched chains^2,31^) and that the probability of each bond of an overstretched backbone to homolyze follows a normal distribution with width σ and the maximum at the middle of the stretched segment (e.g., Supplementary Eq. (14)). Second, we incorporated a common assumption that each C=C bond in a macromolecule reacts independently^55^, which means that the probability of a chain to add an aR^•^ macroradical is proportional to the chain mass. The same applies to an H atom transfer to an aR^•^ radical (e.g., Supplementary Eq. (16)). Finally, we assumed that the reactivity of either aR^•^ or sR^•^ macroradicals is independent of their size or microstructure as suggested by the DFT calculations described above.

**Fig. 4:**
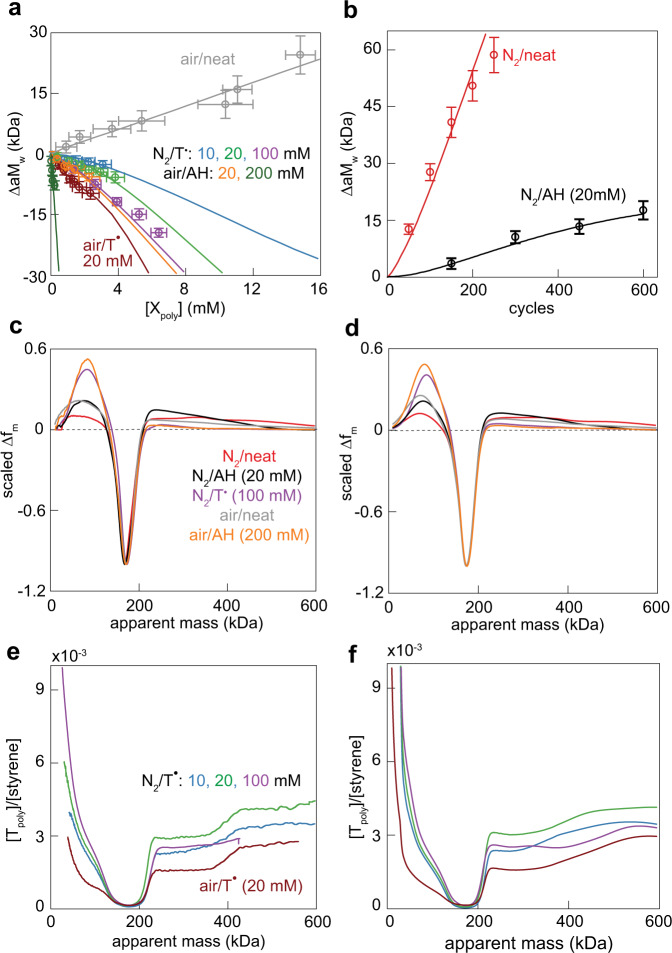
The proposed remodeling mechanism reproduces all experimental observations. **a** Experimental (dots) and simulated (lines) correlations between the concentration of polymer-bound T or OOH moieties, [X_poly_], and change in a*M*_w_; **b** Experimental (dots) and simulated (lines) increase in a*M*_w_ as a function of the number of shearing cycles for the conditions that do not generate polymer-bound T or OOH moieties. Error bars define 2σ confidence intervals. Representative experimental (**c**) and simulated (**d**) differential aMMDs at the end of each shearing experiment. Each differential aMMD was scaled to the same minimum value to facilitate comparisons. See Supplementary Figs. 25 and 23 for all simulated apparent and true MMDs. Distribution of polymer-bound T moiety per styrene, [T_poly_]/[styrene] as a function of the apparent chain mass at the end of each listed experiment (**e**), and the corresponding simulated distribution (**f**). Because larger chains contain more styrene moieties per chain, the approximately constant [T_poly_]/[styrene] vs. mass correlations at >200 kDa correspond to increasing numbers of T moieties per average chain: for example, an average 400 kDa chain with [T_poly_]/[styrene] = 3 × 10^−3^ carries 4.7 ± 0.4 T moieties (Supplementary Fig. 32).

Because the mechanism in Fig. 3 is independent of the overall rate of the material remodelling on the laboratory timescale we set the rate constant for mechanochemical fragmentation of the most abundant chain in the intact polymer (150 kDa), *k*_f_^ref^, to 1 and expressed all other rate constants (Supplementary Table 18), and the remodeling time as multiples of *k*_f_^ref^. We systematically varied the rate constants along with the chain-fragmentation parameters, *m* and σ, to reproduce measured a*M*_w_ as a function of the concentration of either polymer-bound T or OOH, [X_poly_], Fig. 4a. These correlations also yielded the absolute values of *k*_f_^ref^ for neat and AH-doped copolymer sheared in air, and T-doped copolymers sheared in N_2_ and air. The small variations of *k*_f_^ref^ across this range of conditions (average *k*_f_^ref^ = (4.7 ± 0.8) × 10^−4^ cycle^−1^, Supplementary Fig. 29) is consistent with the hypothesis that mechanochemical kinetics in flows is primarily determined by the local strain rate^2,56^, which was identical in all our experiments and independent of the extent of remodeling. We used these absolute *k*_f_^ref^ to confirm that the simulations reproduced measured a*M*_w_ vs. shearing cycles correlations of anaerobically sheared neat and AH-containing polymers (Fig. 4b). These conditions did not generate detectable chain-bound species and therefore lack [X_poly_] vs. a*M*_w_ correlations of the other samples. Our simulations also reproduced the measured distributions of both the apparent chain masses, aMMD, and polymer-bound T moieties, T_poly_ (4c-f and Supplementary Figs. 24, 25). We used a combination of measured and fitted contraction factors^41^ to convert true chain masses in simulations to measured apparent masses.

The relative rate constants that reproduced the experimental correlations were comparable to the corresponding ΔΔ*H*^‡^ from DFT calculations, with the biggest difference of ~3 kcal/mol for the relative rate constants of H-atom abstraction by ROO^•^ vs. aR^•^. This suggests that the mechanism in Fig. 3 reflects the intrinsic reactivities of macroradicals and sp^2^ carbons, and is not specific to the loading mode used in this study. Such conclusion is further supported by the existing literature demonstrating that the same polymer produces the same macroradicals in different mechanical loading scenarios^1,2,6^, including grinding^23^, uniaxial compression of a bulk sample, freeze-fracture, phase transition^57^, and sonication in solution. It also suggests that using relative rate constants from DFT calculations can predict the evolution of the composition of the loaded material accurately enough to reduce the need for measurements.

### Key mechanistic findings

**Fig. 5:**
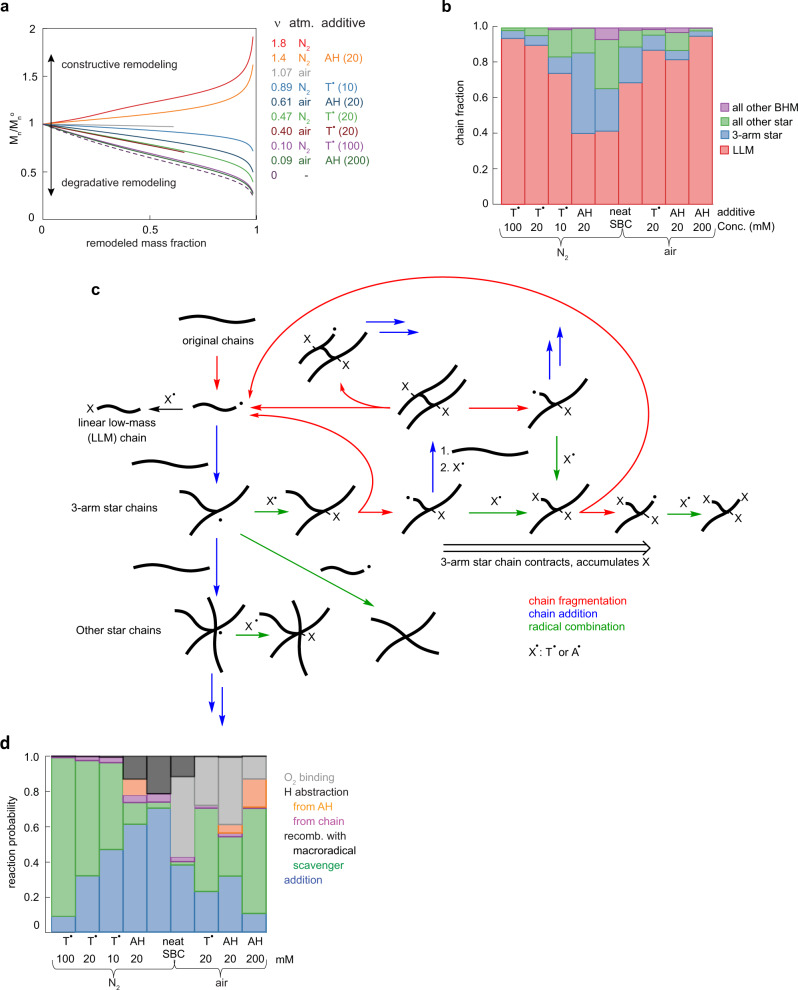
Key findings and predictions of the mechanism. **a** The molar mass of the average chain relative to that of the starting material, *M*_n_/*M*_n_^o^ as a function of the mass fraction of remodeled material; ν is the number of backbone bonds formed per fracture. **b** The composition of the product chains at different polymer compositions. **c** The primary reactions responsible for repeated formation and fracture of branched chains in the mechanically loaded polymer. H-atom transfers are omitted. Chain fragmentation always yields two macroradicals; when they have the same microstructure, only one chain is shown for simplicity. **d** Reaction selectivities of aR^•^ in polymers of different composition. Relatively slow diffusion in the solid allows additions (blue) to compete with barrierless recombinations with T•, A• or O_2_ (green and grey). The equivalent data for sR^•^ and ROO^•^ is plotted in Supplementary Fig. 28.

The data described above is consistent with the formation of new backbone C–C bonds in all mechanically loaded samples. The simulations confirmed that in neat copolymers under N_2_ or air and in AH-doped copolymer under N_2_ the mass of the average chain, *M*_n_ increases during shearing and the number of newly formed backbone bonds per chain fracture, ν, exceeds 1 (Fig. 5a). In other words, the remodeling is constructive. In all other loading conditions the new backbone bond formation reduced the extent but didn’t reverse mechanical degradation, as evidenced by the corresponding *M*_n_ values being below that of the intact sample, *M*_n_°, but exceeding *M*_n_ for the polymer in a dilute sonicated solution, where no backbone bonds reform (ν = 0, dotted line). These examples illustrate the point made in the introduction: a spontaneous bond formation doesn’t guarantee that an average chain of a loaded sample grows, i.e., that the remodeling is constructive instead of degradative.

In all samples, the competition between bond homolysis and regeneration creates mixtures of linear low-mass (LLM) and branched high-mass (BHM) chains (Fig. 5b and Supplementary Fig. 31). Analysis of the [T_poly_]/[styrene] ratios as a function of chain mass suggests that remodeling is mediated primarily by repeated fracture and regeneration of BHM chains (Fig. 5c), while LLM chains contribute little. The [T_poly_]/[styrene] ratios of BHM chains (apparent mass >200 kDa, Fig. 4e and Supplementary Fig. 16) correspond to 4–14 T moieties per chain (Supplementary Fig. 32) and increase almost proportionally with mass. At the experimentally studied remodeling extents, chains incorporate T moieties predominantly by recombination of a macroradical and T^•^, so that the number of T moieties each chain bears increases approximately linearly with the number of times the chain has fractured (Fig. 5c). The multiple T moieties per chain thus mean that an average BHM chain undergoes multiple cycles of mechanochemical fracture (r1, Fig. 3), which reduces its size, chain growth by addition of surrounding chains (r2) and chain termination by recombination. Conversely, LLM chains have ≤1 T per chain irrespective of chain mass, suggesting that once formed, LLM chains are largely inert. This divergent roles of BHM and LLM chains reflect the dependence of reaction probabilities of a chain on its size, which scales either as its contour length to the power of ~2.5 for chain fracture or linearly for the probability of reacting with a macroradical (either by addition or H-atom transfer). Consequently, BHM chains are more likely both to fracture than shorter LLM and intact chains, and to grow even larger by reacting with a radical.

The same trends are expected to lead to preferential peroxidation of largest chains, which may impact how a mechanically stressed material behaves when heated. It may also result in the response of unsaturated polymers to thermomechanical loads, of the types encountered, for example, at the surface of an operating tyre, being qualitatively different from a sum of responses to purely mechanical and purely thermal loads.

In the absence of exogenous reactants, recombination of sR^•^ with aR^•^ is the primary reaction that limits ν (Fig. 5d). This substantial contribution of macroradical recombinations may reflect fast diffusion of unpaired electron along chains by multiple sequential intramolecular additions and/or H-atom shifts (Supplementary Table 8). We cannot yet distinguish between chain-diffusion and radical-diffusion recombination mechanisms because they yield similar distributions of chain sizes and degrees of branching.

Exogenous reactants (T^•^, AH, O_2_) decrease ν by competing for aR^•^, reducing the average size and steady state concentration of these actively growing radicals. Because the rate of macroradical recombinations scales quadratically with their concentration, the contribution of such recombinations to the average chain size is particularly sensitive to the presence of chain-terminating additives. For example, the largest chains in neat copolymer result from recombination of pairs of aR^•^ radicals; all additives completely eliminate this path, but they affect aR^•^+sR^•^ recombinations differently. T^•^ eliminates them by capturing sR^•^ quantitatively, thereby increasing the yield of LLM chains per fracture. Conversely, the negligibly slow reaction of AH with sR^•^ increases the relative importance of aR^•^+sR^•^ recombinations, which enriches the remodeling mixtures in 3- and 4-arm star chain at the expense of larger BHM congeners. This ability to control the distribution of chain topologies by using an additive with different kinetic selectivities towards the macroradicals of different types represents a potentially valuable design tool to control the course of remodeling. Chain growth remains detectable even at highest [T^•^]_bulk_ employed (Fig. 5d) thanks to the relatively slow diffusion of even small-molecule solutes in the polymer and the very fast addition of aR^•^ to adjacent chains thanks to low Δ*H*^ǂ^ (<9.5 kcal/mol, Supplementary Table 4) and high concentration of sp^2^-carbons (Supplementary Table 9).

Unlike T^•^ and AH, O_2_ doesn’t reduce the total concentration of macroradicals, and the distinct patterns of remodeling in N_2_ and air reflect rapid binding of O_2_ to aR^•^ and sR^•^ and the kinetic preference of resulting ROO^•^ for H-atom abstraction over addition (Fig. 5d and Supplementary Fig. 28). In the absence of antioxidants, conversion of ROO^•^ to P_OOH_ by H abstraction increases the flux of sR^•^ (r4, Fig. 3), which allows peroxidation of another chain, eventually yielding 3.9 chain-bound OOH moieties per chain fracture. A comparable yield was speculated to characterize low-temperature radical polymerizations in the presence of O_2_ traces^58^. In both cases, P_OOH_ chains accumulate because they are inert at the respective reaction temperatures. AH reduces the peroxidation yield because diffusion-limited H-atom transfer from AH to ROO^•^ suppresses regeneration of sR^•^. The concomitant increase in the A^•^ flux, which adds rapidly to aR^•^ but is inert to ROO^•^, makes AH nearly as effective at inhibiting chain growth in air as T^•^ (Fig. 5d). Consequently, effective suppression of chain peroxidation while maximizing constructive remodeling requires minimizing O_2_ concentrations in the overstressed volumes of material.

Importantly for prospective applications of macroradical-driven constructive remodeling, O_2_ does not accelerate degradation of the loaded polymer, in contrast to thermal oxidation of polyolefins, which often demonstrate self-accelerating kinetics^59^. The reason is the strong kinetic selectivity of ROO^•^ towards H atom abstraction over any other reaction, including recombination, thanks to low Δ*H*^ǂ^ and the abundance of allylic and benzylic H atoms in the copolymer. H atom abstraction yields a peroxidized chain, P_OOH_ (r4, Fig. 3), whose thermal and mechanochemical reactivity is identical to that of OOH-free chain of the same mass and topology at 10 °C. Conversely, recombination of ROO^•^ with aR^•^ or sR^•^ produces a chain with an O–O backbone bond, which is much more susceptible to homolysis when overstretched than a C–C bond (Supplementary Fig. 22). Whatever O–O bonds do form are distributed randomly with respect to the center of the resulting chains, where the fragmentation probability peaks. This reduces the likelihood of a chain containing an O–O bond to fragment by homolysis of the O–O instead of an C–C bond enough to have no discernable effect on remodeling.

## Discussion

The data above demonstrates autonomous regeneration of backbone bonds in a loaded polymer initiated by macroradical products of chain fracture without the limitations inherent to any previously demonstrated strategy of achieving constructive remodeling, such as the use of mechanochemically-labile reactive moieties or swelling the polymer with reactive monomers. As a result, it’s also free of the constraints that limit the practical utility of the approaches to achieving molecular self-healing reported to date. For example, simulations of the remodeling material confirmed that the number of new bonds formed per chain fracture, ν, does not depend on the fraction of the remaining initial chains because the formed C–C bonds are no more or less likely to dissociate mechanochemically than any other C–C bond at the same fractional displacement from the center of the stretched segment. The steep scaling of the fragmentation probability with chain size makes constructive remodeling self-regulating, i.e., ν decreases weakly as *M*_n_ increases. As larger chains accumulate the average fragmentation rate increases even at constant loading rate, raising the steady-state concentration of macroradicals and accelerating macroradical recombination. The latter reduces ν because it competes with addition of macroradicals to adjacent chains.

Our results demonstrate that forming bonds between intact chains is a more efficient strategy of maintaining or increasing the average chain size under mechanical load than polymerizing monomers. In our experiments, substantial chain growth requires ν of only slightly above 1, because each newly formed bond increases the chain size by a factor of 2 or more. In contrast, the average macroradical needs to add multiple monomers before its mass reaches that of the fractured precursor chain. Difficulty achieving the required kinetic chain lengths in a mechanically loaded polymer may explain why the average degree of polymerization of polymers milled in excess of an unsaturated monomer decreases^21,60^.

The maximum number of new backbone bonds formed per chain fracture, ν_max_, achievable by radical-additions depends on the chemical composition of the backbone, which determines the capacity of the products of chain fracture to initiate addition reactions, and the capacity of the radical acceptor groups to sustain radical propagation. Experimental ν could be lowered below ν_max_ by exogenous reactants with affinity for macroradicals (e.g., O_2_ or antioxidants). Our results suggest that microkinetic simulations using relative rate constants derived from DFT calculations can predict the evolving composition of the remodeling polymer accurately. It is however laborious. The data reported here provides a reference for constraining ν_max_ of other polymers to a plausible range by calculating their yield of macroinitiators per chain fracture, *f*, and bulk pseudo-first order rate constants for addition and H-atom transfer (*k*_a_ and *k*_h_) without further computations (see SI for further details). For example, for poly(1,2-butadiene) *f*, *k*_a_ and *k*_h_ are 1, 2.7 and 0.18 times those of the copolymer studied here and hence its ν_max_ ≫ 1.8 (Supplementary Table 16). For ethylene/vinyl norbornene copolymer these values are 2, 0.80 and 0.05, respectively, suggesting ν_max_ ≫ 1.8 because the higher yields of macroinitiators compensates for slower *k*_a_ and low *k*_h_ inhibits termination. Finally, poly(1,4-butadiene) is estimated to have ν_max_ ~1 because its fragmentation produces only allylic macroradicals which recombine faster than add to the relatively inert C=C bonds of surrounding chains. This illustrates the broader point that autonomous regeneration of C–C bonds in mechanically stressed polymers requires either backbones whose fracture yields highly reactive alkyl macroradicals, or pendant groups that are highly reactive to radical addition, such as acrylates or styrenes.

Above estimates may be sufficiently accurate to prioritize candidate polymer compositions for subsequent microkinetic simulations or measurements because only 3 distinct ranges of remodeling appear to exist. At ν <0.6 chain degradation dominates: branched chains account for <5% of the mass and are unlikely to affect the bulk properties of the polymer. For many purposes, chain/chain addition can simply be ignored. At ν between 0.6 and 1, average aR^•^ grows faster than the average original chain degrades, increasing *M*_w_ until the mass fraction of the remodeled material exceeds ~0.6. Subsequently, accelerated fragmentation of BHM chains and accumulation of LLM chains reverse the trend in *M*_w_, although *Ɖ* continues to increase (Supplementary Fig. 30). Such non-monotonic changes are sometimes observed during high-temperature reactive processing^30^ of polymer melts. Net chain growth (constructive remodeling) is reached at ν >1, resulting in monotonically increasing *M*_n_, *M*_w_ and *Ɖ* as the initial chains are depleted.

Mechanistic interpretations of polymer reactions present conceptual and technical challenges absent in small-molecule studies^51^. A mechanochemically remodeling polymer is comprised of intractably many structurally unique components, each with distinct kinetic and thermodynamic stabilities. Accounting for all this diversity is neither feasible nor necessary because the differences between many such components are simply undetectable and lack practical or conceptual significance. Consequently, a useful mechanism of a reacting polymer is predicated on identifying a logical, internally consistent and ideally generalizable strategy for reducing this complexity (coarse-graining^61^). Here we described a twofold approach. First, the mechanism in Fig. 3 identifies the subset of reactions that we propose are primarily responsible for remodeling under shear. This mechanism enables both qualitative discussions of the chemistry that underlies remodeling and systematic building of reaction networks needed for quantitative simulations of the evolving composition of a remodeling sample. Second, we assumed that the probability of a specific chain to react in any of the reactions of mechanism in Fig. 3 is a simple function of the chain mass and contour length (Supplementary Eqs. (13)–(16) and Supplementary Table 18). This allowed us to use just 12 independent kinetic parameters to define a realistic and tractable reaction network of tens of thousands of components (i.e., chains of unique size, composition and topology) that comprise a remodeling sample. These kinetic parameters reflect the weighted average kinetics of all chains in the sample. The excellent agreement between the observations and microkinetic simulations based on mechanism in Fig. 3 validates our coarse-graining strategy, which is independent of the underlying chemistry.

The importance of highly branched chains in determining the remodeling, revealed by our work, means that quantitative descriptions of remodeling must account for chain topology explicitly. This presents a particular challenge for mechanistic understanding and predictive simulations, because mixtures of branched chains are difficult to characterize experimentally^41,62^, while simulating the complex relationship between the size of a branched chain, its fragmentation probability, and the multitude of microstructures generated both by its fragmentation and by addition of the resulting macroradicals to random locations of other branched chains is resource-demanding. Analysis of mass-dependent distribution of small-molecule chromophores that are incorporated into chains either simultaneously or in competition with new bond formation offers useful mechanistic insights that simplify, accelerate or even reduce the need of full-scale simulations. For radical additions, pyrene-modified stable organic radicals, such as TEMPO, work well. For approaches reliant on small-molecule crosslinkers^10,18,63^, incorporating a chromophore in the crosslinker will likely be similarly effective.

In conclusion, we described the molecular mechanism of spontaneous regeneration of backbone C–C bonds in mechanically-stressed polymer initiated by macroradical products of scission of overstretched polymer chains. We demonstrate that even fairly inert unstabilized C=C bonds support the sufficiently facile formation of new bond for the average chain in the loaded sample to grow despite simultaneous competing chain scissions, providing means to suppress or even reverse mechanical degradation autonomously and in real time. Importantly for prospective practical applications of such constructive remodeling, it is compatible with O_2_ and commercial antioxidants, manifests clear and potentially exploitable dependence on the polymer composition, and requires neither mechanochemically-labile monomers, co-reactants nor complex chain architectures. The reported molecular mechanism enables detailed simulations of the evolving composition of the loaded material over usefully long loading times. Such simulations reproduced the experimental results accurately using relative rate constants similar to those calculated at the DFT level for model radicals. This confirms that the observed mechanochemistry reflects intrinsic kinetics and thermodynamics of the individual steps of the mechanism irrespectively of the specific means of generating the macroradicals or the polymer studied. We also described a simplified method of predicting the competition between bond-forming and bond-breaking reactions without the need for extensive simulations. The presented approach and data provide a quantitative framework for systematic studies of polymers capable of forming new backbone bonds under load and of means to engineer this reactivity in practically relevant materials.

## Methods

### Shearing experiments

Polymer samples were sheared in a custom apparatus, comprised of a purpose-made capillary flow cell coupled to an 831.20 400 Hz Elastomer System from MTS Systems Corporation. This tester controls the magnitude and rate of the vertical displacement and measures the force needed to achieve them. The cylindrical flow cell consists of two reservoirs of 10 mm in diameter and 2.5 cm long (~2 mL capacity) each connected by a capillary of 1 mm diameter and 10 mm length (Supplementary Fig. 5) and two pistons fitting the reservoir chambers. During the operation the two pistons were maintained at a constant vertical separation of 30 mm and the cell was moved up and down at a linear rate of 0.625 mm/s corresponding to a single shearing cycle of 32 s (the oscillation frequency of 0.0315 Hz). The temperature of the cell was maintained constant at 10 ± 2 °C using a cooling coil wrapped conformally around the cell through which a coolant was circulated while the temperature of the cell was monitored by a thermocouple inserted in a borehole orthogonal to the capillary. The cell was placed in a sealed glovebag to control the atmosphere around it and hence the gases dissolved in the sheared melt. The oxygen concentration in the bag was measured with a Setnag oxygen analyser.

In each experiment, the flow cell was filled with the sample (1.4 g) in two steps. First, ~1.2 g of the copolymer was placed in one of the chambers as mm-size pieces, and the cell was subject to 4 shearing cycles, after which the cell was dismantled, and the remaining 0.2 g was added. For anaerobic shearing, all operations were performed under N_2_ atmosphere in the glovebag. Samples of sheared material of ~50 mg each were taken periodically from 3–5 different locations of the upper reservoir and stored under Ar at −37 °C until analysed. Variations of all measured parameters among samples taken from different locations of the reservoir were within the experimental uncertainties, suggesting homogeneity.

We confirmed that the sheared material equilibrates rapidly with the atmosphere around the cell during the experiment. For example, setting up the cell under N_2_ as described above with an N_2_-saturated copolymer sample and replacing the atmosphere surrounding the cell with air yielded the same mass-distribution and degree of peroxidation after >100 cycles as a similar sample that was loaded in the cell in air and the shearing was performed exclusively in air. Because the copolymer oxidizes spontaneously if slowly in air, its exposure to air before shearing should be minimised regardless of whether the sample is intended to be sheared in air or N_2_.

To estimate the contribution of local heating to remodeling of sheared samples, we sheared a 0.1% (mass) solution of anthracene dimer in the copolymer. The dimer is a thermally labile species (Δ*G*^ǂ^ dissociation ~28 kca/mol^64^) which forms anthracene upon mild heating (Supplementary Fig. 9). The characteristic absorption spectrum of anthracene enables its detection in sheared samples when >0.1% of the dimer dissociated during the shearing experiment. No detectable amount of anthracene was produced in sheared dimer-doped samples.

### Sample analysis

THF solutions of sheared material for further analysis were prepared by adding a weighted portion of a sample (15 mg) to an aliquot of freshly distilled THF in a 1.5 mL vial, which was then shaken until the material dissolved. The sample was then transferred to a mini-centrifuge tube and centrifuged at 10,000 rpm (~6200 g in relative centrifugal force) to sediment any solids. For SEC analysis, a 100 µL aliquot of the supernatant was diluted with anhydrous THF to give ~0.5 mg/mL concentration (1.5 mg/mL for T-doped samples).

The concentration of hydroperoxy moieties in sheared melts was quantified by iodometry of dissolved sheared material, by monitoring the absorbance of I_3_^−^ formed in the reduction of hydroperoxy moieties. A 500 µL aliquot of a solution of a sheared sample was transferred to a spectral-glass cuvette, diluted with distilled THF (500 µL), anhydrous isopropanol (300 µL) and acetic acid (100 µL). All additions were performed under N_2_. After recording the absorption spectra of the sample, a solution of NaI in isopropanol (15 mg in 100 μL) was injected into the sample, and the spectra between 300–600 nm were collected every 36 s for 30 min (Supplementary Fig. 8).

Shearing of polymers containing dissolved pyrene-modified TEMPO, T^•^, generates polymer chains bearing covalently bound T moieties when a C-based macroradical recombines with T^•^. Pyrene served as a spectroscopic marker, thanks to its distinct UV-vis spectrum, to enable quantitation of polymer-bound TEMPO by UV-vis analysis of mass-resolved SEC fractions. We deconvoluted the spectrum at each retention time that manifested absorption at both 262 nm and 344 nm (which are absorption maxima for styrene and pyrene, respectively) of >3 mAU to the styrene and pyrene contributions using the reference spectra measured under the same conditions (Supplementary Fig. 15). Note that the [T_poly_]/[styrene] ratios are independent of the chain mass and are therefore valid regardless of how the retention time and the chain mass are related. The total amount of polymer-bound T was quantified by deconvoluting the spectrum of the sample, rather than individual fractions.

### DFT calculations

All computations were performed with the Gaussian 09.E software package at the (u)MPW1K/6–31+G(d,p) level. In addition we calculated six key activation enthaplies with 5 other functionals (Supplementary Table 3) to assess how sensitive the results were to the model chemistry.

To find the minimum number of segments (chains of two or more repeat units terminated by CH_3_ groups, Supplementary Fig. 18) that quantitatively capture the kinetics and stoichiometry of mechanochemical fragmentation of a chain of styrene/butadiene copolymer of an arbitrary length and stretched to an arbitrary force at an arbitrary loading rate (because these parameters in sheared samples are unknown), we divided all backbone bonds of the styrene/butadiene copolymer into those formed during polymerisation (the “between” bonds) and those carried over from each monomer (the “within” bonds). To estimate the kinetics of mechanochemical fragmentation of an arbitrary “between” bond of a stretched chain, we calculated force-dependent activation free energies of fragmentation of the “between” bond in all 9 pairwise combinations of the 3 repeat units comprising the copolymer (Fig. 1 and Supplementary Fig. 17). To confirm that the calculated activation barriers are not biased by the relatively close proximity of the scissile bond to the atom at which force acts, which is a direct result of using small molecules to mimic the behaviour of a long polymer chain^65^, we repeated the calculations for 2 longer homologues. The force-dependent free energies were insignificantly different for both pairs of homologues, validating our choice of two-repeat unit segments to model macrochains.

In the absence of force homolysis of C–C bonds in these segments lacks transition states. To locate the transition states for mechanochemical fracture of a C–C backbone, we first optimized the longest conformer of each segment mentioned above with its _Me_C^…^C_Me_ distance constrained with a very soft virtual harmonic spring to the restoring force of 4.5 nN (implemented with the iop(1/164) overlay procedure of Gaussian). We then performed a relaxed potential energy scan (rPES) of the scissile bond in this externally constrained molecule, followed by Berny optimization of the scan point with the highest electronic energy to a (constrained) transition state.

The calculations of force-dependent activation energies followed the previously described^66^ and theoretically^67^ and experimentally^16,68^ validated method. First, we performed rPES on the longest conformer of each kinetically-significant stationary state by increasing its _Me_C∙∙∙C_Me_ distance stepwise up to the restoring force of 6 nN. These scans yielded electronic energies and constrained distances at multiple increasingly large values of the stretching force. Second, we calculated analytically frequencies of a subset of the converged scan points to estimate force-dependent thermodynamic corrections. Such calculations are theoretically sound because they are performed on the molecule plus its infinitely-compliant constraint (rather than just the molecule), which is a stationary point with all internal forces at 0^67,69^. Finally, we interpolated electronic energies, distances and thermodynamic corrections to yield continuous Δ*G* and Δ*H* as a function of stretching force up to 6 nN (Supplementary Eq. (5)).

### Microkinetic mechanistic simulations

We simulated the remodeling of the copolymer predicted by the mechanism in Fig. 3 by representing every chain by a specific number of unbreakable inert repeat units, U, of 2.5 kDa each, connected by massless linkers each capable of linking an arbitrary number of U. All chemistry happens at a linker. The system is closed, of constant volume and spatially homogeneous, with no concentration gradients. The homogeneity of sheared samples was confirmed experimentally as described above. We ensured the finite size of the model without violating the conservation of mass by assuming that only chains whose concentration exceeds a threshold value react.

Fracture of a branched chain and addition of a macroradical to another chains create product chains of different topologies (see Supplementary Tables 16, 17 for examples). We manually defined the relationship between the distribution of product chain topologies for each reactant chain topology of up to 4 branch points, which allowed us to create product distributions programmatically (see the Supplementary Information for further details). We described each chain by a size vector, which is a sequence of integers in specific order that uniquely define the size of each chain segment and their relative position in the molecule, and related to the chain topology by systematic notations (Supplementary Table 15). A chain segment is a portion of a chain between two branch points or between a terminus and a branch point. Each number of the size vector is the number of U in each segment. A linear chain has a single segment and is coded by a single integer. An n-arm star chain is coded by a sequence of n integers in increasing order, each describing the size of each arm.

In sheared material a chain of each structure is present in several chemically distinct forms, each comprising a unique component. For example, each linear chain, U_n_, exists as a closed-shell macromolecule, and as a radical, with the unpaired electron at a terminus or at any of the ~n/2 unique linkers. Thus, the composition of a mixture is defined by a set of pairs of vectors, with each size vector paired with a concentration vector that specified the concentrations of various forms of the chain whose structure is defined by the size vector. The length and the structure of the concentration vector depend on the number of chemically-distinct species being tracked. For example, each linear chain in anaerobically-sheared neat copolymer and samples containing AH is characterized by a 4-element concentration vector, corresponding to the concentrations of the closed-shell form, a terminal alkyl radical (aR^•^), a terminal stabilized radical (sR^•^), and an internal radical, which can only be stabilized (because it can only be produced by H atom transfer from a linear chain). Because the unpaired electron was equally likely to localize between any pair of U, so that only the total concentration of internal macroradicals, rather than the concentration of each isomer of such macroradicals, need to be specified.

## Supplementary information

Supplementary Information

## Data Availability

the data described in this paper is collected in the data.mat file deposited with the University of Liverpool DataCat research data catalog (10.17638/datacat.liverpool.ac.uk/1697).

## Code availability

Matlab code for generating the distributions of product chains resulting from chain additions or mechanochemical chain fracture, and illustrative examples of input/output data sets are available in file archive deposited with the University of Liverpool DataCat research data catalog (10.17638/datacat.liverpool.ac.uk/1697).
