# Supplementary material for: The molecular mechanism of constructive remodeling of a mechanically-loaded polymer
Source: Nat Commun. 2022 Jun 7;13:3154. doi: 10.1038/s41467-022-30947-8 (PMC9174275; doi:10.1038/s41467-022-30947-8)
Supplement: Supplementary file 1 — Supplementary Information [file 41467_2022_30947_MOESM1_ESM.pdf]

The molecular mechanism of constructive remodeling of a mechanically-loaded polymer.

Chenxu Wang†[,1], Sergey Akbulatov†[,1], Qihan Chen[1], Yancong Tian[1], Cai-Li Sun[1], Marc Couty*[,2], Roman Boulatov*[,1]

Supplementary Material

Supplementary Material 1
Materials and Methods 3
Materials 3
Styrene-Butadiene Copolymer 3
Doped copolymer 5
Equipment 7
Flow cell for multipass capillary shearing 7
Instruments for characterization 7
Experimental protocols 8
Shearing experiments 8
Other shearing conditions and polymer masses studied 9
Sample preparation for analysis 10
Hydroperoxide determination by iodometry 10
Spectrophotometric quantitation of carbonyl groups. 11
Spectrophotometric quantitation of OH and CO_2H groups. 11
Experimental validation of negligible heating during shearing. 11
Data processing protocols 12
Calculation of apparent mass distributions. 12
Calculation of number of T moieties per styrene 17
DFT calculations 19
General 19
Kinetics of mechanochemical chain fragmentation 20
Kinetics and structure-reactivity relationships of macroradical reactions. 24
Fracture of O-O bond in the backbone under mechanical load 29
Estimated diffusion-limited rate constants 30
Kinetic simulations based on the reaction mechanism 31
Distribution of product chain topologies 32
Code for generating product distributions. 34
Macroscopic and microscopic rate constants 35
Fitting strategy 37
Estimates of the contraction factors 38
Estimates of the confidence intervals on fitting parameters 39
Appendix 1: numerical description of chain structures and concentrations 45
Supplementary references 49

Table of figures

Supplementary Fig. 1 NIR (a) and 1H NMR (b) spectra of the copolymer used in our study. 4
Supplementary Fig. 2 Synthesis of pyrene-TEMPO ($T^{\bullet}$). 5
Supplementary Fig. 3 ^{1}H-NMR (a) and ^{13}C APT NMR (b) spectra of pyrene-TEMPO in $CDCl_3$. 6
Supplementary Fig. 4 HRMS spectrum of pyrene-TEMPO. 6
Supplementary Fig. 5 A schematic of the flow cell assembly used for shearing experiments. 7
Supplementary Fig. 6 Forces during shearing. 8
Supplementary Fig. 7 Summary of the results of shearing experiments under non-standard conditions. 10
Supplementary Fig. 8 Representative example of spectrophotometric data from iodometry. 10

Supplementary Fig. 9 Quantitation of local heating during shearing. 12
Supplementary Fig. 10 Examples of measured apparent molar mass distributions...................................13
Supplementary Fig. 11 The differential apparent mass distributions, obtained by subtracting the MMD of the intact sample from all subsequent apparent MMDs shown in the preceding figure. 14
Supplementary Fig. 12 The RI output of SEC of sheared samples. 15
Supplementary Fig. 13 The PDA output (262 nm) of SEC of sheared samples. 16
Supplementary Fig. 14 The PDA output (375 nm) of SEC of sheared T-doped samples. 16
Supplementary Fig. 15 Illustrative data for determination of [T_{poly}]/[styrene] ratios..................................17
Supplementary Fig. 16 Representative examples of measured [T_{poly}]/[styrene] ratios. 18
Supplementary Fig. 17 The three repeat units comprising styrene-butadiene copolymer, along with their molecular weights and molar fractions in the copolymer used by us. 19
Supplementary Fig. 18 The mechanochemical kinetics of fragmentation of ''between'' bonds of a chain of the styrene/butadiene copolymer. 21
Supplementary Fig. 19 Segments for calculations of the free energy of fragmentation of the ''within'' bonds. 21
Supplementary Fig. 20 Calculated stoichiometry of chain fracture. 24
Supplementary Fig. 21. Different types of C and H atoms in the copolymer. 28
Supplementary Fig. 22 Computed effect of O-O backbone bonds on chain fracture. 30
Supplementary Fig. 23 Simulated molar mass distributions (MMDs) whose aM_w most closely match corresponding measured values for each shearing condition. 39
Supplementary Fig. 24 Simulated [T_{poly}]/[styrene] ratios.. 40
Supplementary Fig. 25 Simulated apparent molar mass distributions (aMMDs) that most closely match each measured aM_w for each shearing condition. 40
Supplementary Fig. 26 Simulated apparent differential mass distributions obtained by subtracting the mass distribution of the intact sample from those of sheared samples at each cycle shown. 41
Supplementary Fig. 27 Simulated [T_{poly}]/[styrene] ratios averaged over all chains with the apparent chain mass in each 100-Da wide bin that most closely match each experimental total [T_{poly}] value. 41
Supplementary Fig. 28 Reaction selectivities of sR$^•$ (a) and ROO$^•$ (b) macroradicals under different conditions. 42
Supplementary Fig. 29 Correlation between simulation reaction time (unitless) and shearing cycles based on matching [T_{poly}]. 42
Supplementary Fig. 30 Number- and weight-average molar masses of the remodelling samples, M_n and M_w; the number-average molar mass of the product chains and the dispersity index of the sample as a function of the remodelled mass fraction. 43
Supplementary Fig. 31 The fractions of chains of different topologies in the product mixtures as a function of the remodeled mass fraction...43
Supplementary Fig. 32 The average number of T moieties per chain of each mass at the end of each shearing experiment based on the contraction factors estimated from simulations..................................44

Table of tables

Supplementary Table 1 The concentrations of t-BuOOH solution determined by iodometric titration.......11
Supplementary Table 2 The reactions used for computational model of chain fragmentation kinetics. 22
Supplementary Table 3 The effect of the functional on the calculated activation enthalpies of key reactions underlying remodeling. 24
Supplementary Table 4 The activation/reaction enthalpies (in kcal/mol) for addition of alkyl or stabilized radicals to a side or backbone C=C bond at uMPW1K/6-31+G(d,p) level..25
Supplementary Table 5 Calculated activation/reaction enthalpies (in kcal/mol) for addition of diverse $RO_2^•$ to an sp^2 C atom or abstraction of an allylic H atom at uMPW1K/6-31+G(d,p) level. 26
Supplementary Table 6 Standard free energy of barrierless binding of O_2 to alkyl and stabilised radicals (in kcal/mol) at uMPW1K/6-31+G(d,p) level in vacuum. 26
Supplementary Table 7 The activation/reaction enthalpies (in kcal/mol) of H abstraction by an alkyl and stabilized radicals at uMPW1K/6-31+G(d,p) level...26

Supplementary Table 8 Reaction, ΔH^o, and activation, Δ$H^‡$, enthalpies of intramolecular reactions of alkyl radicals. 27
Supplementary Table 9 Concentration (M) of reactive functional moieties in the styrene/butadiene copolymer used in our studies. 27
Supplementary Table 10 Estimated relative pseudo-1st order rate constant (s^{-1}) for reactions of aR$^•$, sR$^•$ and ROO$^•$ macroradicals based on DFT calculations and the concentrations of various atoms in neat copolymer. 28
Supplementary Table 11 Reaction, ΔH^o, and activation, Δ$H^‡$, enthalpies of key reactions between macroradicals and small-molecule solutes and related reactions. 28
Supplementary Table 12 Calculated activation enthalpies, Δ$H^‡$ (kcal/mol) under standard conditions for shown recombination reactions. 29
Supplementary Table 13 Calculated activation energies, Δ$G^‡$ (kcal/mol) under standard conditions for β-scission of radicals at the uMPW1K/6-31+G(d,p) level in the gas phase. 29
Supplementary Table 14 Estimated diffusion coefficients of T$^•$ and AH and the diffusion-limited rate constants for reactions of O_2, T$^•$ and AH with macroradicals in sheared copolymer at 10 °C based on DFT calculations. 31
Supplementary Table 15 The notations of different chain topologies used in the code provided. 32
Supplementary Table 16 An example of the method for determining the product distribution and stoichiometry of fragmentation of a branched chain. 33
Supplementary Table 17 An example of the method for generating size vectors of the products of addition of a macroradical to a closed-shell chain for a terminal macroradical. 34
Supplementary Table 18 The definitions of the rate constants used in the simulations and the corresponding model reactions used for DFT calculations of the reaction mechanism. 35
Supplementary Table 19 Estimates of the number of C-C bonds formed by radical addition per fractured C-C bonds, ν, based on the relative rate constants of addition to sp^2 carbons and abstraction of allylic H atoms using the calculated values for styrene/butadiene copolymer, ν^{ref}, k_a^{ref} and k_h^{ref}.. 44
Supplementary Table 20 Examples of size and concentration vectors for different chain topologies and shearing conditions. 47

Materials and Methods

Materials

Styrene-Butadiene Copolymer

All experiments described below used a random copolymer of styrene and butadiene of M_n = 151±5 kDa, M_w = 165±2 kDa and T_g = -35 °C, containing 30.5±0.5% by weight of styrene, 27.1±0.7% by weight of 1,2-bound butadiene, with the rest being 1,4-bound trans and cis-butadiene (trans/cis ratio of 1.5:1) based on NIR measurements (see below).

The polymer was synthesized by anionic polymerization of styrene and butadiene in methylcyclohexane initiated by *n*-BuLi. In a typical procedure 550 mL of anhydrous methylcyclohexane was introduced into a pressurized stirred reactor followed by an injection of styrene (12 mL, 0.10 mol), butadiene (48 mL, 0.57 mol) and 320 μL of unstabilized anhydrous THF as a polar additive. The polymerization is started by adding a solution of *n*-BuLi in methylcyclohexane (1.96 mL, 0.122 mol/L) at 50°C. The polymerization was quenched when the monomer conversion reached 80%, by adding an excess methanol. The solvent and unreacted monomers were evaporated under vacuum at 60°C for 24 hours and the dry product was sealed under Ar in a pouch for storage at -30°C to avoid any contact with oxygen and light prior to its use.

The composition of the polymer was confirmed by FTNIR spectroscopy using TENSOR 37 BRUKER spectrometer on a film following the previously validated approach.[1] The ^{1}H NMR spectrum of the polymer dissolved in CS_2/C_6D_{12} confirmed by statistical distribution of the distinct repeat units. The spectrum was recorded in a magnetic field strength of 11.4 T with a BRUKER AVANCE III HD console and a 5 mm cryoprobe in a 30° One Pulse experiment (zg30), a Time Domain of 32k and a spectral width of 16 ppm. The FID was zero-filled to 64k and a 0.3 Hz line broadening apodization was applied before Fourier Transfor-

mation. The absence of styrene blocks of longer than 6 repeat units is evident by the absence of resonances at <6.55 ppm); all aromatic protons absorb at 6.0 - 7.5 ppm, consistent with a random distribution of styrene.

a

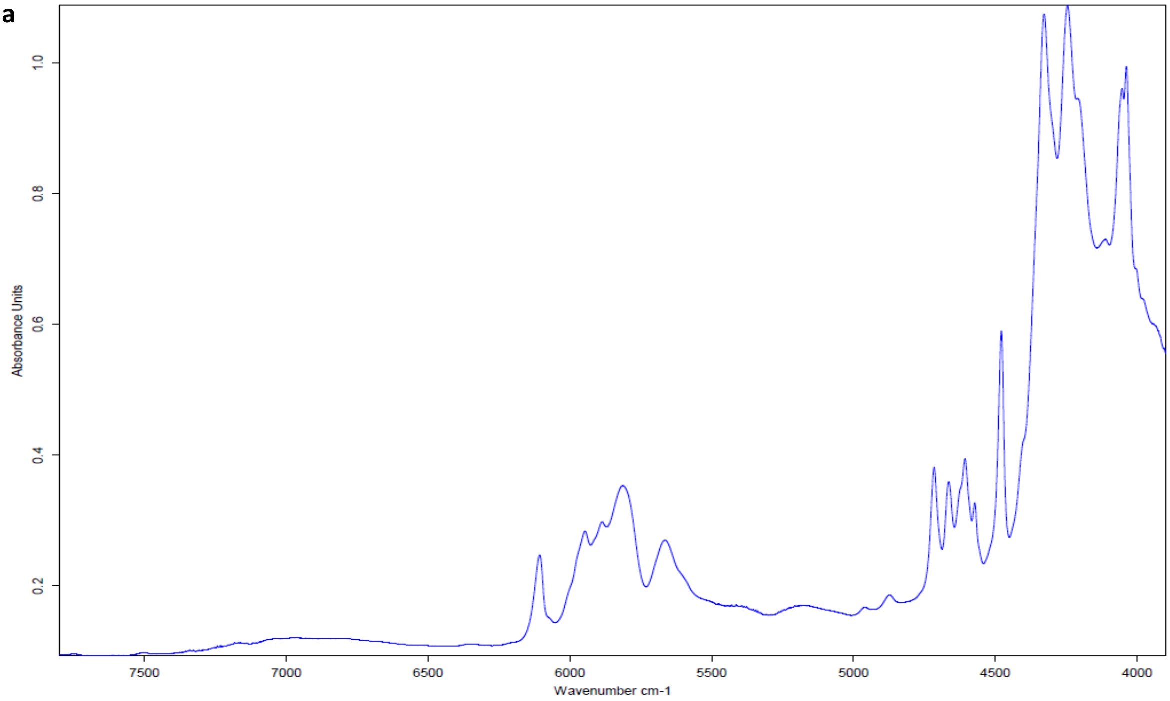

b

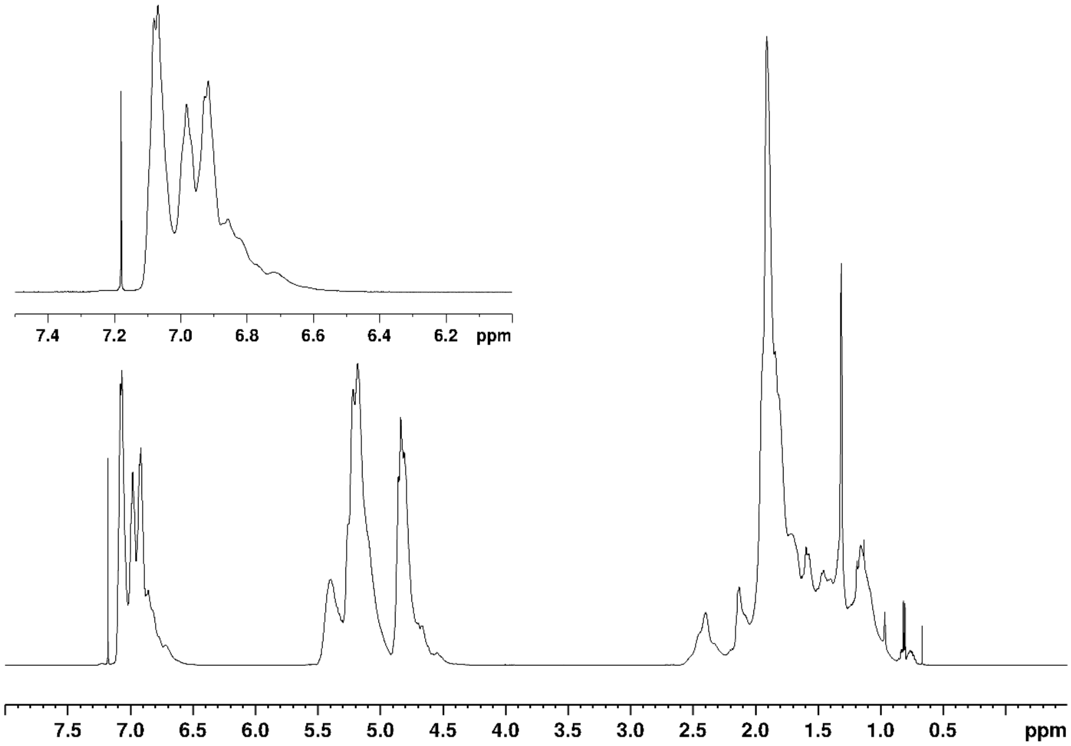

Supplementary Fig. 1 NIR (a) and 1H NMR (b) spectra of the copolymer used in our study.

Doped copolymer

In addition to additives-free copolymer, we sheared samples containing dissolved commercial antioxidant, AO2246 (AH, Fig. 1 in main text, purchased from VWR in 97% purity) or a stable organic radical, pyrene-modified TEMPO ($T^{\bullet}$, Fig. 1 in main text). TEMPO binds C-based radicals in a barrierless reaction, and characteristic absorption of pyrene enabled us to quantify the number of TEMPO moieties bound to polymer chains of various masses.

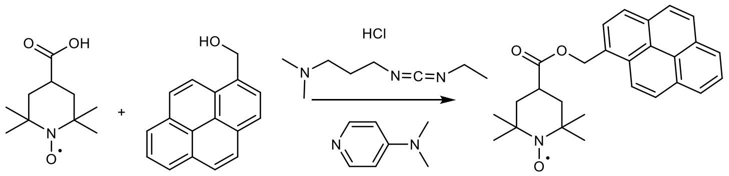

Supplementary Fig. 2 Synthesis of pyrene-TEMPO ($T^{\bullet}$).

We synthesised $T^{\bullet}$ by adding a solution of EDC (24.89 mg, 0.130 mmol) in DCM (0.4 mL) to a mixture of commercial 4-carboxy-TEMPO (20 mg, 0.100 mmol), pyrene-1-ylmethanol (34.8 mg, 0.150 mmol) and DMAP (1.22 mg, 10 µmol) in DCM (0.4 mL) in a glovebox under N_2 atmosphere. After stirring the reaction mixture for 24 h at room temperature, we evaporated the solvent under reduced pressure and purified the residue by flash chromatography on silica under gradient conditions of pure hexane to 50% EtOAc in hexane to yield the product pyrene-TEMPO (20 mg, 48 %). We characterised the product by ^{1}H and ^{13}C NMR and HRMS (Supplementary Figs. 3-4). Chemical shifts: ^{1}H NMR spectrum: δ 8.40-8.00 (m, 9H, Pyrene), 5.95 (br. s, 2H, O-CH_2); ^{13}C APT NMR spectrum: δ 132.0, 131.3, 130.7, 129.7, 128.7, 128.4, 128.1, 128.0, 127.4, 126.3, 125.8, 125.6, 125.0, 124.8, 124.7, 122.8. HRMS spectrum: [M+Na]+ calc'd for $C_{27}H_{28}NNaO_3$ (m/z) 437.1961, found 437.1970.

a

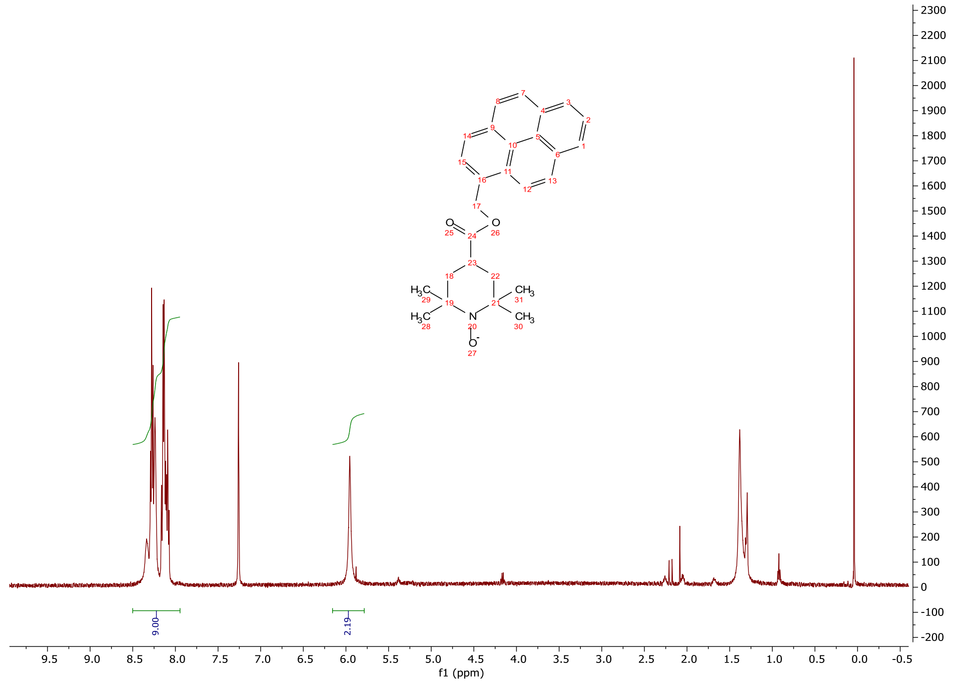

b

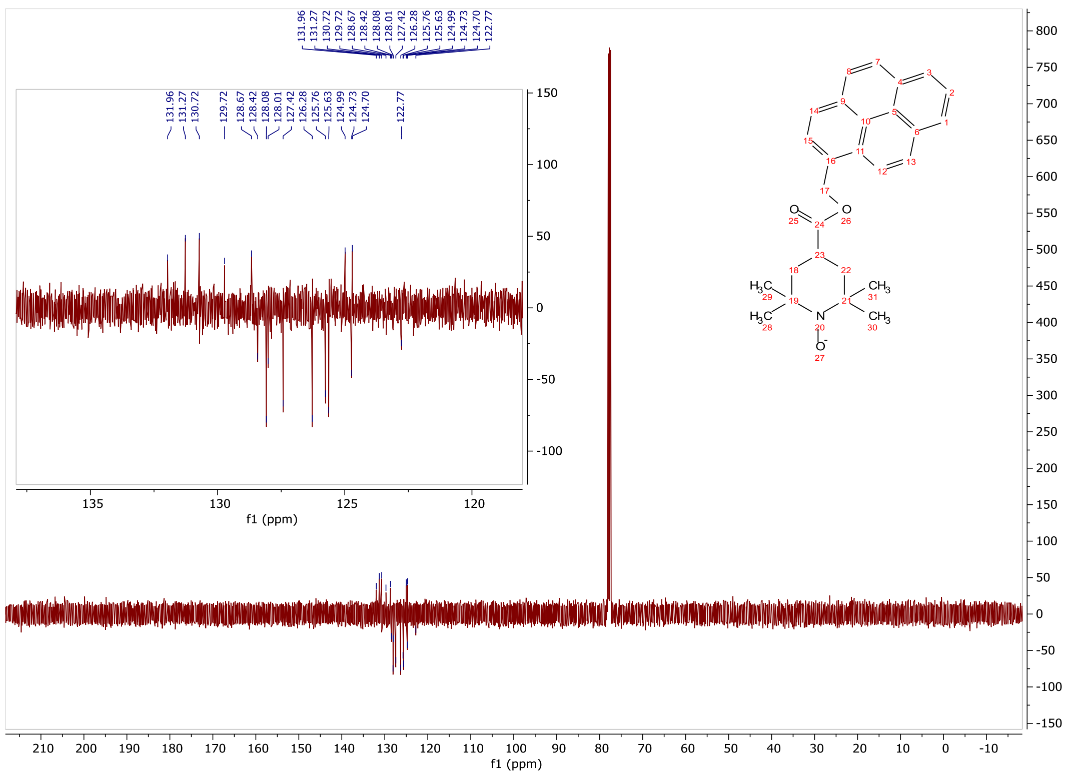

Supplementary Fig. 3 ^{1}H-NMR (a) and ^{13}C APT NMR (b) spectra of pyrene-TEMPO in $CDCl_3$ at 500 MHz and 126 MHz, respectively.

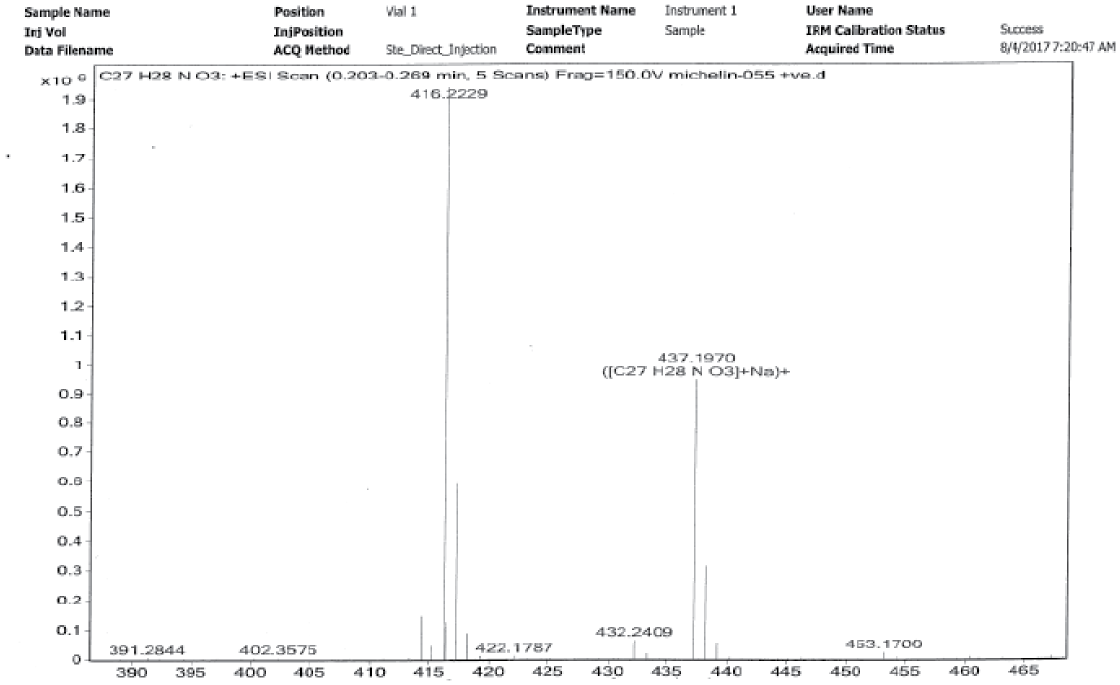

Supplementary Fig. 4 HRMS spectrum of pyrene-TEMPO.

We prepared doped polymer samples by swelling additives-free copolymer (1.4 g) with a toluene solution (7 mL) of the additive (either T$^{\bullet}$ or AH) at the desired concentration under N_2 until all liquid was absorbed. Vials containing swollen polymer were dried under high vacuum for 48 hrs. The dried samples manifested no signs of phase separation.

TEMPO abstracts allylic and benzylic H atoms with the calculated free energy of activation of 31.6 kcal/mol (and measured rate constant at 25 °C of 10^{-11} s^{-1}) and the generated allylic/benzylic radicals then

bind another TEMPO molecule. Consequently, solid solutions of pyrene-TEMPO in copolymer must be stored at -37 °C or below and used as soon after preparation as possible.

Equipment

Flow cell for multipass capillary shearing

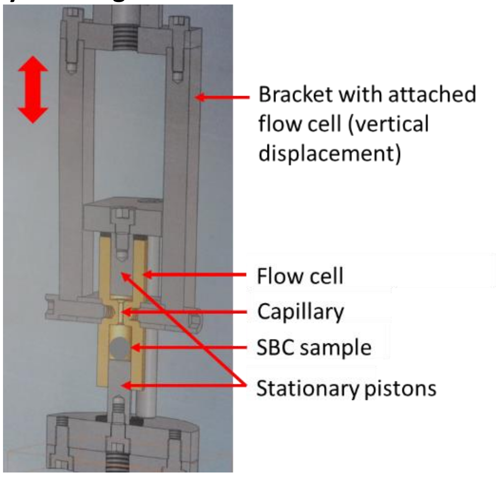

Supplementary Fig. 5 A schematic of the flow cell assembly used for shearing experiments.

Polymer samples were sheared in a custom apparatus, comprised of a purpose-made capillary flow cell coupled to an 831.20 400Hz Elastomer System from MTS Systems Corporation. This tester controls the magnitude and rate of the vertical displacement and measures the force needed to achieve them. The cylindrical flow cell consists of two reservoirs of 10 mm in diameter and 2.5 cm long (~2 mL capacity) each connected by a capillary of 1 mm diameter and 10 mm length (yellow in Supplementary Fig. 5) and two pistons fitting the reservoir chambers. During the operation the two pistons were maintained at a constant vertical separation of 30 mm and the cell was moved up and down at a linear rate of 0.625 mm/s corresponding to a single shearing cycle of 32 s (the oscillation frequency of 0.0315 Hz). For comparison, the terminal relaxation time of styrene/butadiene copolymer of comparable composition is estimated at <10 s based on the data in ref. [2] and the zero-shear viscosity at 10 °C of 7 MPa·s).

The temperature of the cell was maintained constant at 10±2 °C using a cooling coil wrapped conformally around the cell through which a coolant was circulated while the temperature of the cell was monitored by a thermocouple inserted in a borehole orthogonal to the capillary. The cell was placed in a sealed glovebag to control the atmosphere around it and hence the gases dissolved in the sheared melt. The oxygen concentration in the bag was measured with a Setnag oxygen analyser.

Instruments for characterization

Analytical SEC was carried out on Waters Acquity UPLC system with an isocratic solvent manager, sample manager, column heater, photodiode array (PDA, wavelength rage: 240 - 400 nm) detector and RI detector using three Waters Acquity APC columns in series (APC XT 900, 150 × 4.6 mm, APC XT 450, 150 × 4.6 mm, APC XT 200, 150 × 4.6 mm). The flow rate of THF was 0.6 mL/min and sample injection volume 10 μL. The column heater, RI flow cell and sample manager temperatures were 35.0±0.3, 35.0±0.1 and 15.0±0.1 °C, respectively. All polymer solutions were filtered through PTFE syringe filters (pore size: 0.45 μm) prior to analysis. The SEC columns were calibrated in the range of 250 Da - 2.5 MDa using 28 narrow polystyrene standards from Sigma-Aldrich and Scientific Polymers Inc. The Mark-Houwink coefficients, estimated using Viscotek TDA 302 triple-detector, at 35 °C in THF were: 0.712±0.004 and $(1.28\pm0.03)\times10^{-4}$ for polystyrene standards and 0.693±0.008 and $(4.10\pm0.09)\times10^{-4}$ for linear styrene/butadiene copolymer used in our studies. These values are within the range of literature values for the same conditions.[3]

The use of Waters Acquity UPLC system offers considerably greater mass resolution than the conventional GPC, which was essential for experimental validation of simulated mass distributions and hence the mechanism. However, only specially designed detectors, using the same diameter microbore tubing as the rest of the solvent-handling system, can provide output without degrading mass resolution. At the time of this work, only PDA and RI detectors were fully compatible with Acquity UPLC. Because fractions

eluting from the columns contain mixtures of chains of different sizes and microstructures, any detector output represents some averaged parameters of the fraction. Because no additional detector would yield absolute masses of each component of these mixtures,[4] nor obviate the need to fit contraction factors, we avoided using conventional GPC for analysis of sheared samples.

UV-vis spectra were measured with a Cary 50 UV-vis spectrometer equipped with a multi-sample thermostated holder from Quantum Technologies with the temperature set at 25 °C.

High-resolution mass spectrometry (HRMS) was performed on a Micromass LCT TOF Mass Spectrometer at the University of Liverpool Mass Spectrometry Laboratory. Nuclear magnetic resonance (NMR) spectra were recorded on Bruker 500 MHz (Advance III HD) spectrometer.

Although NMR is sometimes useful for detailed characterization of structural aspects hyperbranched polymers, including the degree of branching,[5] both ^1H and ^{13}C solution NMR spectra of remodelled neat polymer (either aerobically or anaerobically) were very similar to those of intact copolymer, except for considerable broadening of the signals.

Experimental protocols

Shearing experiments

In each experiment, the flow cell was filled with the sample (1.4 g) in two steps. First, ~1.2 g of the copolymer was placed in one of the chambers as mm-size pieces, and the cell was subject to 4 shearing cycles, after which the cell was dismantled, and the remaining 0.2 g was added. For anaerobic shearing, all operations were performed under N_2 atmosphere in the glovebag. Samples of sheared material of ~50 mg each were taken periodically from 3-5 different locations of the upper reservoir. Samples were stored under Ar at -37 °C until analysed. Variations of all measured parameters among samples taken from different locations of the reservoir were within the experimental uncertainties, suggesting homogeneity.

A typical force profile over a single shearing cycle is illustrated in Supplementary Fig. 6a. Because the sample does not take up the whole volume of a single reservoir of the flow cell, at the beginning of each half-cycle little force is required to displace the cell relative to the plungers because the material is compressed but not pushed through the capillary. Once the free volume is displaced, the force required to continue the displacement increases rapidly to >2 kN as the melt starts moving through the capillary (and hence sheared).

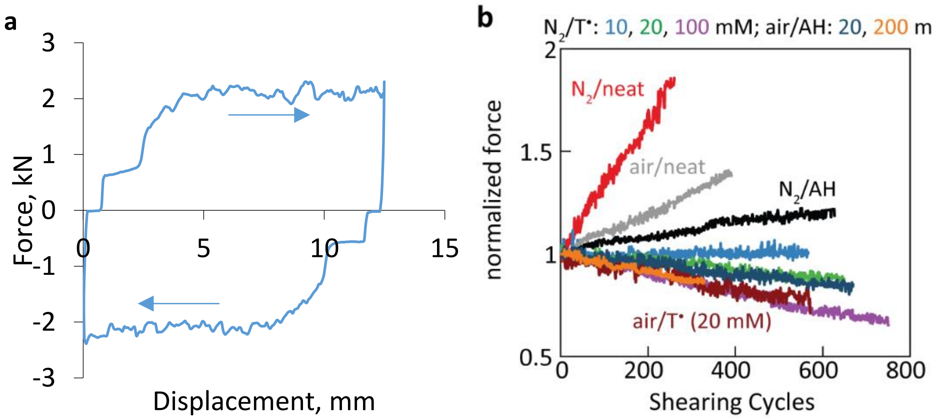

Supplementary Fig. 6 Forces during shearing. (a) A single shearing cycle. The upward movement of the piston (the displacement increasing) generates a positive force, followed by downward movement (the displacement decreasing), which generates the negative force. (b) Normalized force (as the average of the two plateau regions of each cycle) for representative samples of each unique composition.

We confirmed that the sheared material equilibrates rapidly with the atmosphere around the cell during the experiment. For example, setting up the cell under N_2 as described above with an N_2-saturated copolymer sample and replacing the atmosphere surrounding the cell with air yielded the same mass-distribution and degree of peroxidation as a similar sample that was loaded in the cell in air and the shearing was performed exclusively in air. Because the copolymer oxidizes spontaneously if slowly in air, its exposure to

air before shearing should be minimised regardless of whether the sample is intended to be sheared in air or N_2.

Other shearing conditions and polymer masses studied
The shearing parameters used in our study, including the size of the polymer were optimized to maximize the accuracy of the correlations in Figs. 4a-b (main text), and hence of the kinetic modeling by screening experimental control parameters in preliminary experiments. A larger copolymer (M_n = 186 kDa, M_w = 212 kDa) sheared neat under N_2 at the standard shearing frequency reached force of 4.5 kN, which is the maximum value that our flow cell is rated to operate at, after only 58 shearing cycles. Accurate quantitation of the evolution of the composition of such a sample would have required us to sample it every ~10-12 cycles (vs. ≥50 cycles for the measurements described above). Such frequent sampling presented both technical issues and risked skewing the results because it takes 3-5 cycles for force to reach a steady value after each sampling. The normalized differential apparent MMD of this larger copolymer is very similar to that of the M_n = 151 kDa sample used for most measurements (Supplementary Fig. 7a), confirming that the remodelling mechanism is insensitive to the initial polymer size within the range studied. Conversely, shorter styrene/butadiene copolymer (M_n = 69 kDa, M_w = 72 kDa) remodeled negligibly over 24 h of anaerobic shearing (Supplementary Fig. 7), which is the maximum duration of a single shearing experiment, making it incompatible with our current instrument.

Aerobic shearing of M_n=151 kDa sample at 3 °C (the minimum temperature accessible in our experiments) and at 25 °C didn't indicate any temperature effects on the product distribution of mechanochemical remodelling, as evident by the corresponding differential apparent MMDs being indistinguishable from those measured at 10 °C (Supplementary Fig. 7). We avoided shearing at temperatures >25 °C to minimize the contribution of thermal remodeling to changes in the composition of the sheared material. We chose the linear displacement rate of 0.625 mm/s to minimize the magnitude of the variations of the plateau force (Supplementary Fig. 6), which increased rapidly with the rate. We do not know the physical mechanism responsible for these force fluctuations but they may indicate flow instabilities at linear rates >1 mm/s.[6]

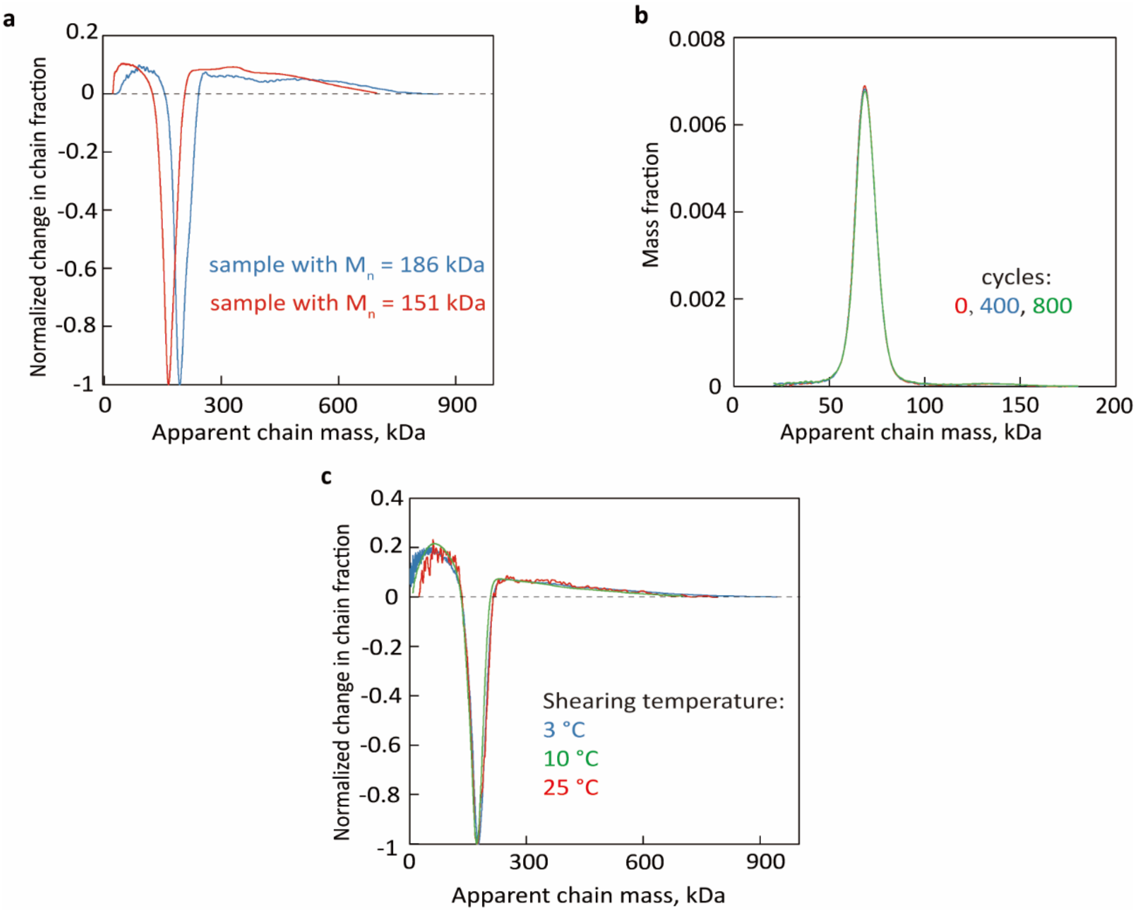

Supplementary Fig. 7 Summary of the results of shearing experiments under non-standard conditions. (a) average normalized apparent mass distributions from anaerobic shearing at 10 °C of neat copolymer with different M_n values; (b) aparent mass distributions from anaerobic shearing of neat copolymer at 10 °C with M_n = 69 kDa at 0, 400 and 800 shearing cycles, demonstrating no changes in composition; (c) average normalized apparent mass distributions from aerobic shearing at different temperatures. The distribution at 25 °C is noisy because the degree of conversion achievable over 24 h of shearing was low. Normalized differential mass distributions are independent of the degree of remodeling, allowing comparisons between samples of different remodelling extent.

Sample preparation for analysis

THF solutions of sheared material for further analysis were prepared by adding a weighted portion of a sample (15 mg) to an aliquot of freshly distilled THF in a 1.5 mL vial, which was then shaken until the material dissolved. The sample was then transferred to a mini-centrifuge tube and centrifuged at 10,000 rpm (~6200 g in relative centrifugal force) to sediment any solids. For SEC analysis, a 100 µL aliquot of the supernatant was diluted with anhydrous THF to give ~0.5 mg/mL concentration (1.5 mg/mL for T-doped samples).

Hydroperoxide determination by iodometry

The concentration of hydroperoxy moieties in sheared melts was quantified by iodometry of dissolved sheared material, by monitoring the absorbance of I_3^- formed in the reduction of hydroperoxy moieties by I^- ($3I^-(aq) + H_2O_2(aq) + 2H^+(aq) \rightarrow I_3^-(aq) + 2H_2O$). A 500 µL aliquot of a solution of a sheared sample was transferred to a spectral-glass cuvette, diluted with distilled THF (500 µL), anhydrous isopropanol (300 µL, Sigma-Aldrich) and acetic acid (100 µL, Sigma-Aldrich, 99.9995% pure). All additions were performed under N_2. After recording the absorption spectra of the sample, a solution of NaI in isopropanol (15 mg in 100 µL) was injected into the sample, and the spectra between 300 – 600 nm were collected every 36 s for 30 min.

The concentration of hydroperoxy moieties per unit mass of the copolymer, c_{OOH}, was calculated by Eq. (1)

$$c_{OOH} = \frac{(\Delta\Delta A_s - \Delta\Delta A_b)V}{\varepsilon \times l \times m}\ mol \cdot g^{-1} \tag{1}$$

where ε = 25000 $M^{-1}cm^{-1}$ is the extinction coefficient of I_3^- in isopropanol at 365 nm, l = 1 cm is the length of the optical path, m is the amount of the copolymer in the sample, V = 1.5 mL is the volume of the sample and $\Delta\Delta A$ is given by Eq. (2), where 365 nm and 600 nm are wavelengths at which the absorbance is measured, and 0 min and 30 min are the time since addition of NaI when it is measured; subscripts s and b refer to the sample solution and blank (all components except the copolymer).

$$\Delta\Delta A = A(365\ nm, 30\ min) - A(365\ nm, 0\ min) - \left(A(600\ nm, 30\ min) - A(600\ nm, 0\ min)\right) \tag{2}$$

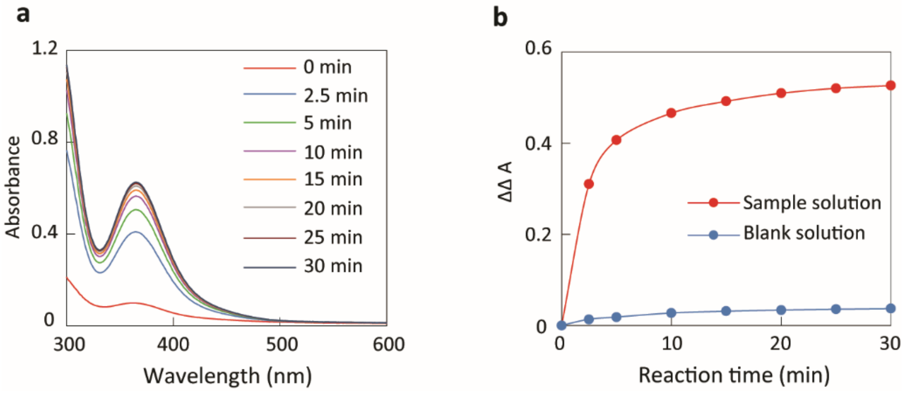

Supplementary Fig. 8 Representative example of spectrophotometric data from iodometry. (a) spectra; (b) the change in the absorbance at 365 nm as a function of the reaction time. The difference between the sample and background absorption plateaus at ~25 min. Only every 10th sample is shown.

The systematic error on the concentration of hydroperoxide measured by iodometric titration was estimated by titrating a solution of known concertation of tert-butyl peroxide (t-BuOOH) in H_2O-isopropanol solution. In one case, the solution also contained pristine copolymer at 5 mg/mL concentration. The results (Supplementary Table 1) showed the relative systematic error of <2%, regardless of the presence of the copolymer, suggesting that functional groups present in intact copolymer do not interfere with the titration.

Supplementary Table 1 The concentrations of t-BuOOH solution determined by iodometric titration in the presence or absence of intact copolymer where within 2% of the true values.

Sample	Prepared concentration, µM	Measured concentration, µM	Relative systematic error
t-BuOOH-isopropanol	19.4	19.2	0.01
t-BuOOH-isopropanol		19.4	0
t-BuOOH-isopropanol+ rubber stock solution		19.0	0.02

Spectrophotometric quantitation of carbonyl groups.
We modified a reported method[7] based on the formation of hydrazone, which has characteristic absorbance at 367 nm with the extinction coefficient of 22,000 $M^{-1}cm^{-1}$. A 100 µL aliquot of a THF solution of aerobically remodelled copolymer was first treated with PPh_3 to reduce OOH moieties, as previously described,[8] by mixing it with 10 µL of 0.05 M solution of PPh_3 in toluene/THF (1:1 vol). After 24 h at room temperature in the dark, copolymer was precipitated by adding 100 µL of EtOH and dried under vacuum for 12 h. After redissolving precipitated copolymer in 100 µL of THF, 10 µL of 0.2 M solution of 2, 4-dinitrophenylhydrazine hydrochloride (DNPH) in THF was added, followed by 10 µL of 1 M HCl. After stirring the solution at 25 ^oC for 3 h under N_2, the solvents were evaporated and the residue was washed with EtOH (3x100 µL), dried under vacuum and redissolved in THF for spectrophotometric determination of the [DNPH]/[styrene] ratios as described above for T-modified samples. In tests of the copolymer oxidized at 60 ^oC in air, [DNPH]/[styrene] ratios $<5\times10^{-4}$ were reliably detected. We confirmed that the detected DNPH was polymer-bound by demonstrating the appearance of the DNPH absorption in UV-vis spectra of SEC fractions as described for polymer-bound T.

Spectrophotometric quantitation of OH and CO_2H groups.
We adopted a previously reported preparative method.[9] A mixture of a THF solution of aerobically remodelled copolymer (100 µL), and 30 µL of CH_2Cl_2 solution of dicyclohexylcarbodiimide (7.1 mM), 4-dimethylaminopyridine (4.1 mM) and either 9-anthracenecarboxylic acid or 9 anthracenemethanol, depending on whether the OH or CO_2H groups are being detected (8.0 mM) was stirred at 0 ^oC for 5 h under N_2, followed by 24 h under ambient conditions in the dark. The solvents were evaporated under vacuum, the residue washed with acetone (3x100 µL) and dried for 12 h before redissolving it in THF for spectrophotometric analysis as described above. The method was validated using commercial OH and CO_2H terminated polystyrenes (M_n = 10.9 and 12.8 kDa, respectively), which manifested the expected 1.0±0.1 anthracene moieties per chain in SEC analysis of the treated solutions.

Experimental validation of negligible heating during shearing.
To estimate the contribution of local heating to remodeling in mechanical experiments, we sheared a 0.1% (mass) solution of anthracene dimer, diAnt, in the copolymer. diAnt is a thermally labile species ($\Delta G^‡$ ~28 kca/mol) which forms anthracene upon mild heating (Supplementary Fig. 9). The characteristic absorption spectrum of anthracene enables its detection in sheared samples when >0.1% of the dimer dissociated during the shearing experiment.

diAnt-containing samples was prepared using the same protocol as copolymer samples containing $T^•$ and AH (above). We confirmed that anthracene is generated when diAnt-containing copolymer is heated to 80 ^oC under N_2, as evidenced by the increase in the absorbance of such material in the 350 – 410 nm spectral window. In contrast, no detectable increase in the absorbance of sheared sample was observed under standard anaerobic shearing.

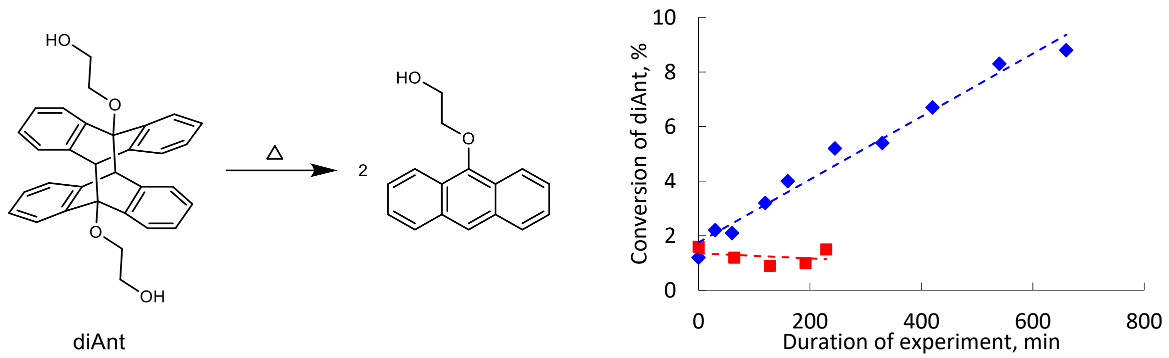

Supplementary Fig. 9 Quantitation of local heating during shearing. Thermal dissociation of di-anthracene (**di-Ant**) yields anthracene chromophore when a sample of the copolymer containing 0.1% (mass) of diAnt is heated under N_2 at 80 ^oC (blue dots and line). No detectable amount of anthracene was produced when the same sample was sheared (red dots and line).

Data processing protocols

Calculation of apparent mass distributions.

The Acquity SEC output has the sampling rate of 0.1 s^{-1}, i.e., each retention time/absorbance or retention time/change in refractive index pair corresponds to a fraction of the sample in a 0.1 μL volume of the eluant. Because of the exponential relationship between the retention time and the apparent chain mass, the mass range contained in every 0.1 μL volume of the eluant varies with the retention time. Proper conversion of the measured detector output to mass distributions requires partitioning this output into bins of equal size of mass (rebinning). We achieved this first by splitting each 0.1 μL bin equally into 10 0.01 μL bins, converting the retention time of each bin into apparent mass of the copolymer using the calibration curve and the Mark-Houwink's parameters and redistributing the detector output into bins of 100 Da each, followed by normalizing the total output to 1 to obtain mass fractions. The resulting apparent mass distributions are shown in Supplementary Fig. 10. The corresponding SEC outputs (RI and PDA detectors at 262 nm and 375 nm; the latter for T-doped samples only) are shown in Supplementary Fig. 12, Supplementary Fig. 13, Supplementary Fig. 14).

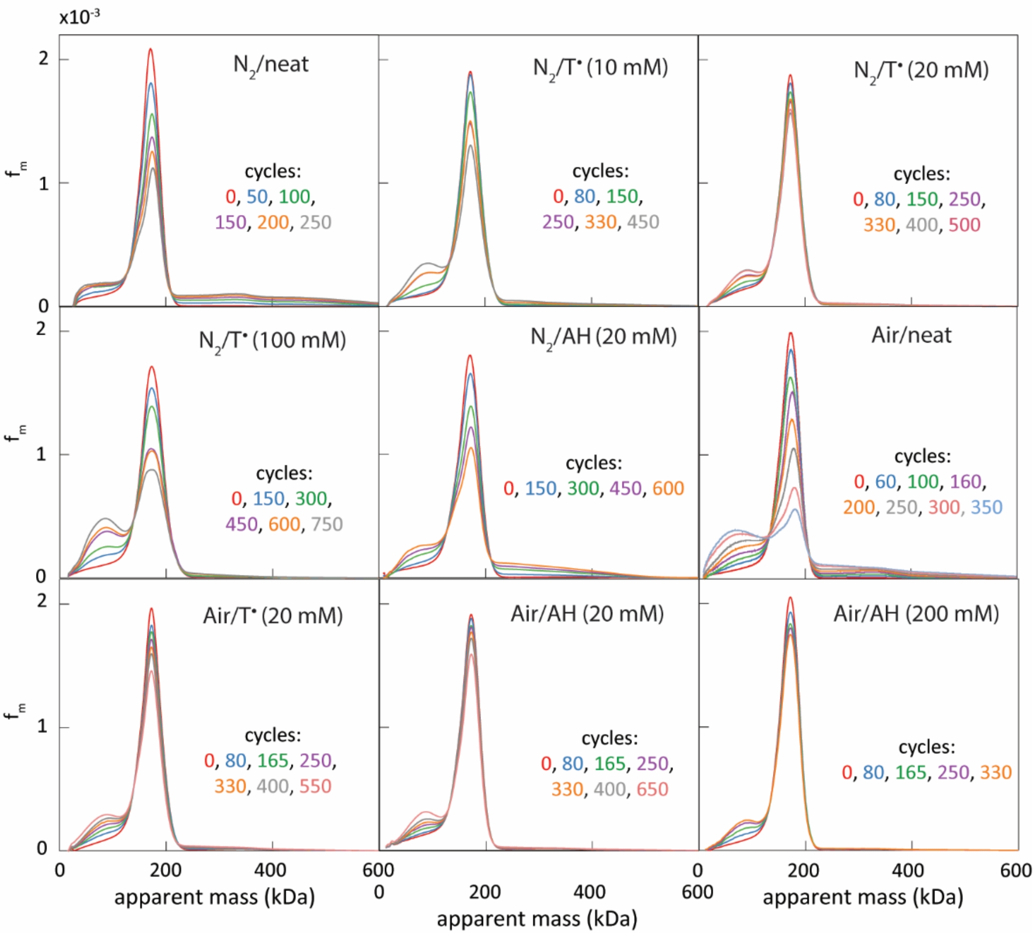

Supplementary Fig. 10 Examples of measured apparent molar mass distributions. The corresponding SECs are in the supplementary data.mat file.

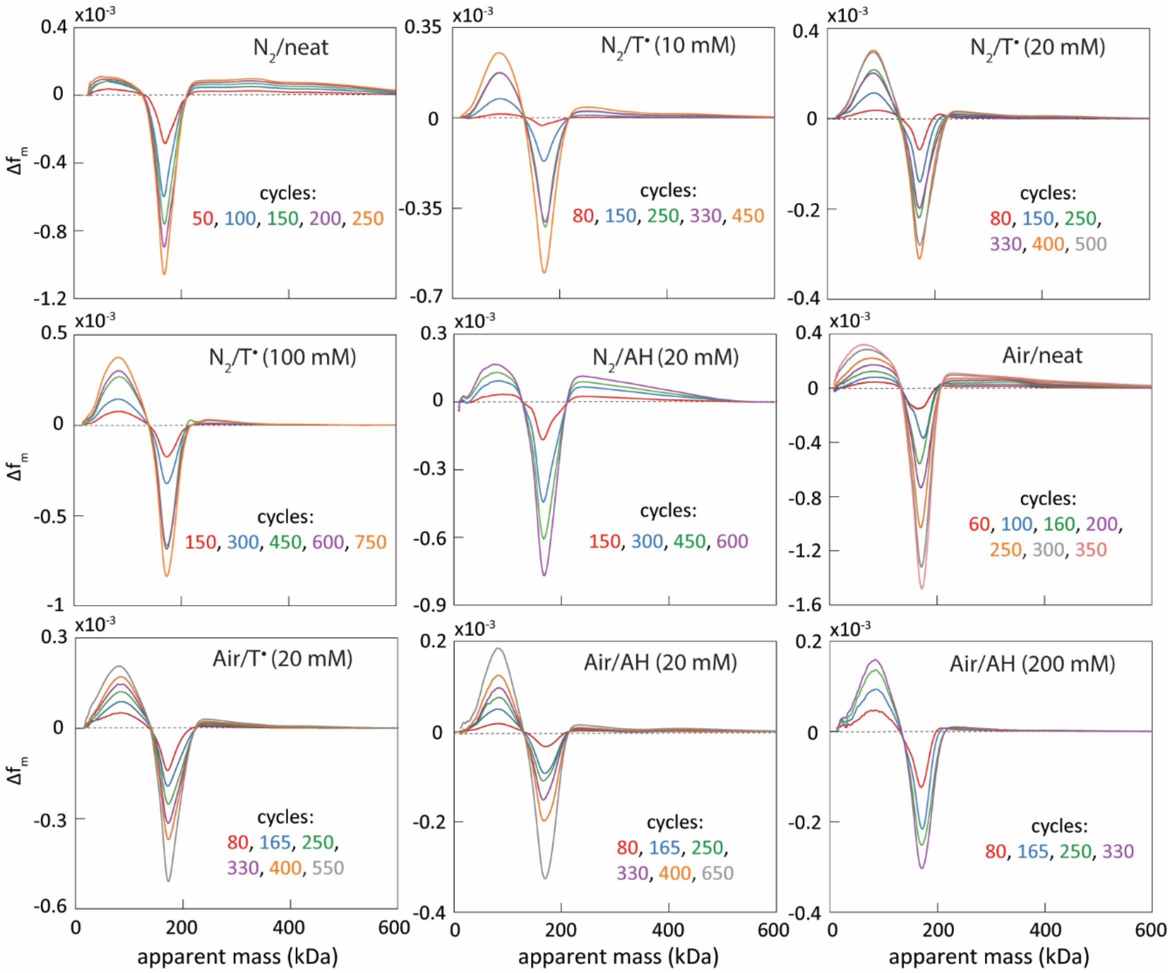

Supplementary Fig. 11 The differential apparent mass distributions, obtained by subtracting the MMD of the intact sample from all subsequent apparent MMDs shown in the preceding figure.

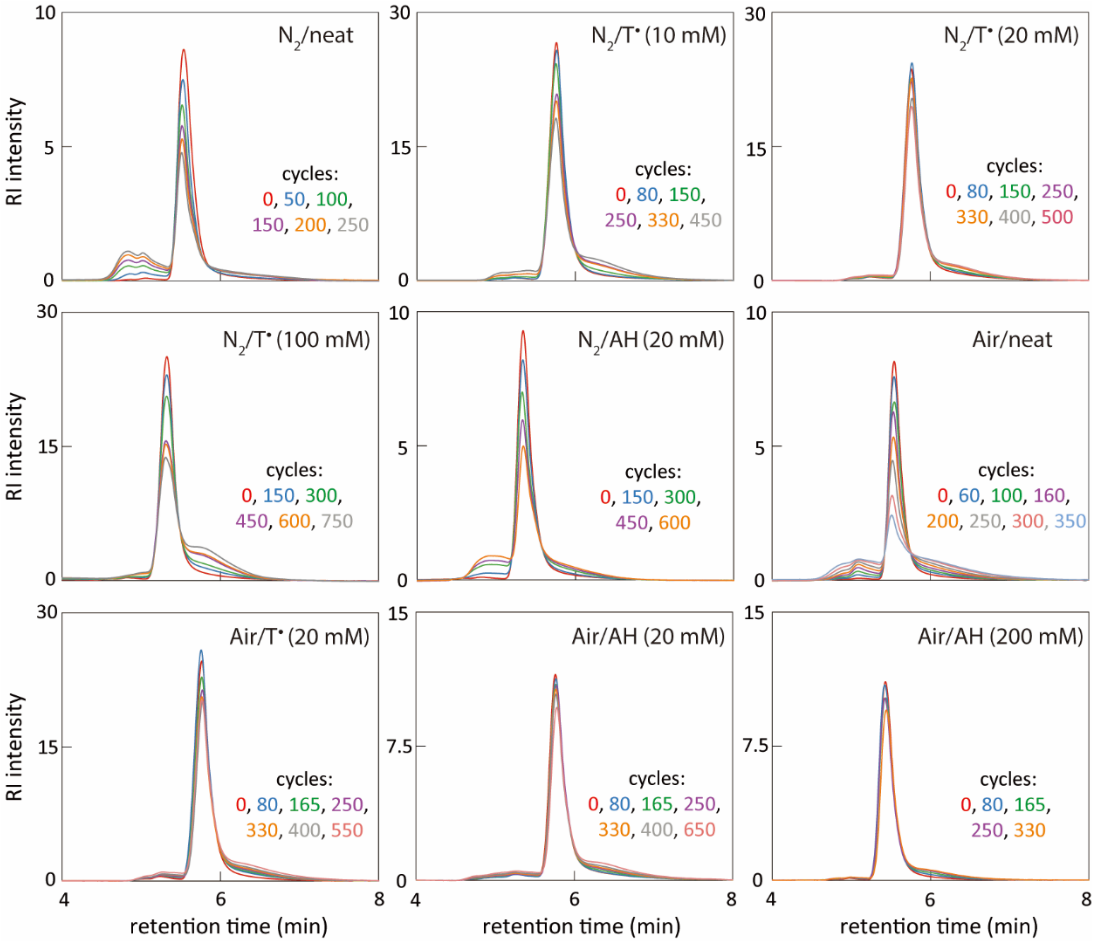

Supplementary Fig. 12 The RI output of SEC of sheared samples.

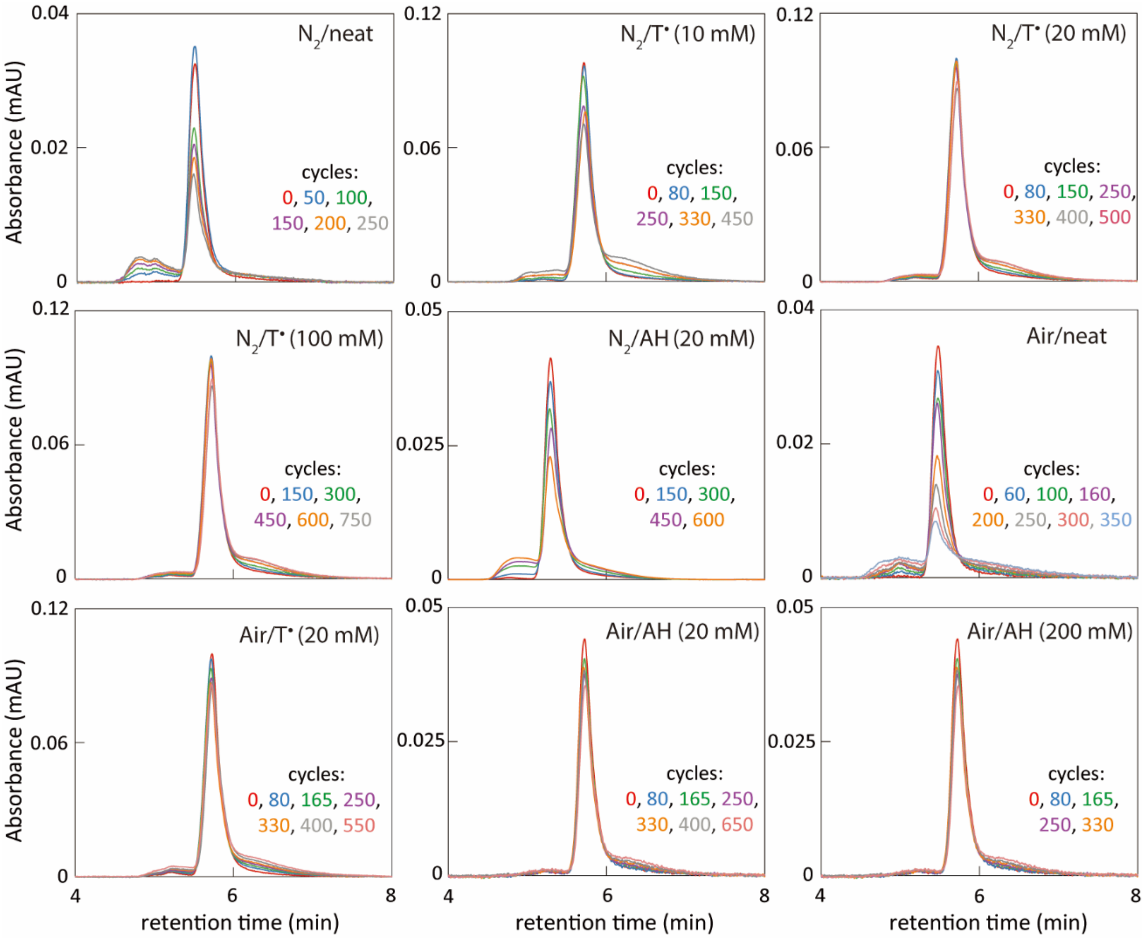

Supplementary Fig. 13 The PDA output (262 nm) of SEC of sheared samples.

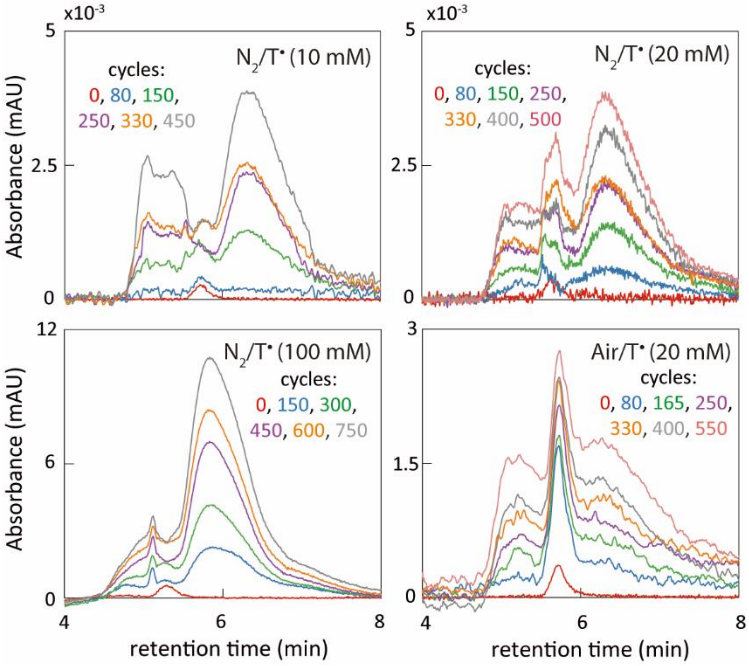

Supplementary Fig. 14 The PDA output (375 nm) of SEC of sheared T-doped samples.

Calculation of number of T moieties per styrene

Shearing of polymers containing dissolved pyrene-modified TEMPO, $T^{\bullet}$, generates polymer chains bearing covalently bound T moieties when a C-based macroradical recombines with $T^{\bullet}$. Pyrene served as a spectroscopic marker, thanks to its distinct UV-vis spectrum, to enable quantitation of polymer-bound TEMPO by UV-vis analysis of mass-resolved SEC fractions. We deconvoluted the spectrum at each retention time that manifested absorption at both 262 nm and 344 nm of >3 mAU (which are absorption maxima for styrene and pyrene, respectively) to the styrene and pyrene contributions using the reference spectra measured under the same conditions (Supplementary Fig. 15). Note that the [T_{poly}]/[styrene] ratios are independent of the chain mass and are therefore valid regardless of how the retention time and the chain mass are related. The total amount of polymer-bound T was quantified by deconvoluting the spectrum of the sample, rather than individual fractions. In all cases, the confidence intervals of the fitted parameters, *S*, were derived from the Jacobian, *J*, returned by the lsqnonlin function of Matlab using Cholesky decomposition for inversion of Jacobian ((4), where *iJ* is inverse *J*, *chol* is Cholesky decomposition, *I* is the identify matrix of the same size as $J'\times J$, *D* is the matrix of the experimental data that was fitted and *n* is the number of independent experimental observations). Relative fitting errors were 0.8 – 9% for mass-resolved data and 0.2 – 0.9% for total T_{poly} at 2σ. The errors of data in Figs. 2c and 4a (main text) are dominated by the statistical (reproducibility) uncertainty.

$$iJ = chol(J' \times J)\backslash(chol(J' \times J)'\backslash I) \tag{3}$$

$$S = \frac{1.96}{n}\sqrt{(D - J \times iJ \times J' \times D)' \times (D - J \times iJ \times J' \times D) \times diag(iJ)} \tag{4}$$

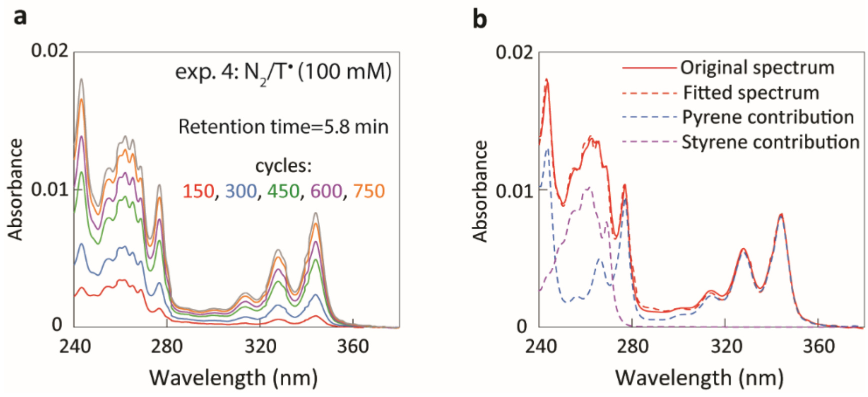

Supplementary Fig. 15 Illustrative data for determination of [T_{poly}]/[styrene] ratios. (a) A sequence of single-retention-time spectra and (b) a deconvoluted spectrum showing the contributions of styrene and pyrene.

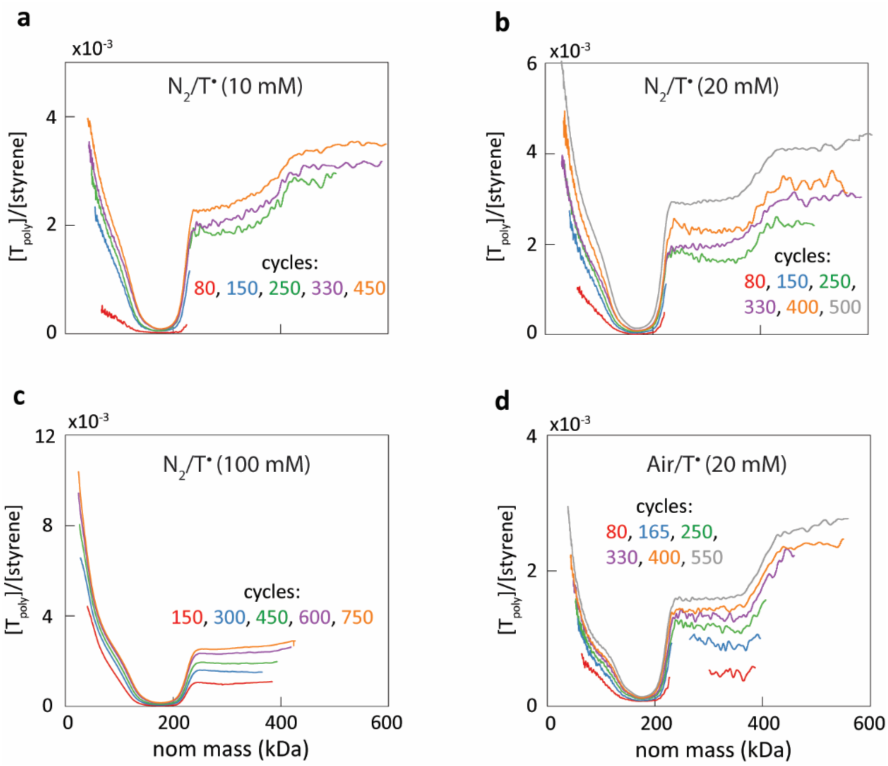

Supplementary Fig. 16 Representative examples of measured [T_{poly}]/[styrene] ratios for T-doped samples sheared in N2 (a-c) or air (d). The gaps in the ratios at low cycles in (d) and truncation of ratios at different apparent chain masses is due to the absorption intensity at the corresponding retention times being below the minimum threshold (3 mAU at 262 and 344 nm).

DFT calculations

General

In most mechanically stressed polymers, mechanochemical fragmentation of overstretched macrochains by homolysis of a backbone bond is the main source of macroradicals. Because styrene-butadiene copolymer chains are made of random sequences of 3 repeat units (Supplementary Fig. 17), each of its backbone bonds is unique and its fragmentation generates a very large number of structurally distinct macroradicals, whose subsequent reactions (including reactions with small-molecule solutes, such as O_2, as well as with adjacent polymer chains) create even more structurally distinct species. Most are highly reactive intermediates whose steady-state concentrations are below the detection limit of the existing analytical techniques for characterising polymeric materials. The huge number of species and reactions responsible for mechanochemical remodelling means that any effort to develop a plausible molecular mechanism of remodelling needs guidance from quantum-chemical calculations.

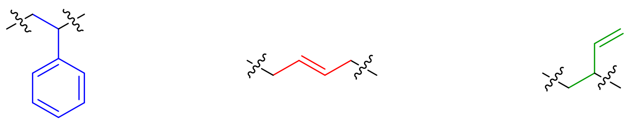

1
1-phenylethanediyl
MW=104.2 g/mol
15.4% (mol)

2
trans-but-2-en-1,4-diyl
(1,4-enchained butadiene)
MW=54.1 g/mol
50.0% (mol)

3
but-3-en-1,2-diyl
(1,2-enchained butadiene)
MW=54.1 g/mol
34.6% (mol)

Supplementary Fig. 17 The three repeat units comprising styrene-butadiene copolymer, along with their molecular weights and molar fractions in the copolymer used by us. *Cis*-but-2-en-1,4-diyl unit is not shown because its fraction is small and its isomerisation to the *trans* analogue is accelerated by tensile load.

Kinetic barriers of radical reactions (including those of molecular fragmentation that produces radicals) can be calculated with useful accuracy (likely deviation from true activation enthalpies of <3 kcal/mol) on hydrocarbons with fewer than ~50 non-H atoms. Consequently, quantum-chemical description of copolymer remodelling must not only systematically reduce the number of reactions needed to describe the underlying chemistry but also use fairly small molecules to represent reactivity of macromolecules. This coarse-graining relies on DFT-level calculations of reaction and activation energies to identify structurally distinct intermediates with sufficiently similar reactivity to be represented by a single species. The “effective” or “lumped” kinetics and thermodynamics of these representative species combined with microkinetic modelling (see below) allowed us to predict the observable chemical manifestations of remodelling (e.g., changes in the fractions of chains of different masses, the concentrations of polymer-bound hydroperoxy or T moieties) with useful accuracy.

All the DFT calculations were performed with the Gaussian 09E software package. The Berny algorithm was applied to locate stationary points. Tight convergence criteria and ultrafine integration grids were used in optimisations and frequency calculations. Thermodynamic corrections (*TC*s) to electronic energies of individual molecules were calculated statistical-mechanically in the pseudo-harmonic oscillator/rigid rotor/ideal gas approximations, as $3RT + ZPE + U_{vib} - TS$, where *ZPE* is the zero-point energy, U_{vib} is the vibrational component of the internal energy and *S* is the total entropy. Vibrational frequencies below 500 cm^{-1} were replaced with 500 cm^{-1} as previously recommended,[10] to avoid the artifactually high contribution of such low-frequency modes to the vibrational entropy. The use of analytical frequencies calculated on converged force-coupled geometries in this study is theoretically sound because the calculation is performed on the molecule plus its infinitely-compliant constraint (rather than just the molecule), which is a stationary point with all internal forces at 0.[11,12]

For reactions between macroradicals, we compared enthalpies rather than free energies because entropies of a reaction between a macroradical and an adjacent chain in neat polymer is likely small as the translational and rotational motion of such chains is already strongly restricted. For reactions between a small-molecule solute (e.g., O_2, radical scavengers or antioxidants) and a macroradical we calculated free energies using the reaction entropy calculated in the ideal-gas approximation scaled by a factor of 0.2, as previously recommended.[13]

Guess structures for transition states of reactions between a radical and a closed-shell molecule were obtained by relaxed potential energy scans (rPESs) of the breaking or the forming bond, followed by calculation of the analytical frequencies on the highest-energy structure and Berny optimization of the candidates with the plausible reactive vibration to a saddle point. In the absence of force, the transition states for homolysis of a C-C or a O-O bond do not exist. To locate the transition states for mechanochemical fracture of a C-C backbone, we first optimized the longest conformer of each segment mentioned above with its $_{Me}C{\cdots}C_{Me}$ distance constrained with a very soft virtual harmonic spring to the restoring force of 4.5 nN (implemented with the iop(1/164) overlay procedure of Gaussian). We then performed a relaxed potential energy scan (rPES) of the scissile bond in this externally constrained molecule, followed by Berny optimization of the scan point with the highest electronic energy to a (constrained) transition state.

As expected, inclusion of reaction solvent (modeled as SMD parameterized for n-hexane and THF) affected the calculated activation free energies minimally, with the mean absolute difference for reactions in Supplementary Table 3 of 0.1 and 0.3 kcal/mol for hexane and THF, respectively.

Kinetics of mechanochemical chain fragmentation

To find the minimum number of segments (chains of two or more repeat units terminated by CH_3 groups, Supplementary Fig. 18) that quantitatively capture the kinetics and stoichiometry of mechanochemical fragmentation of a chain of styrene/butadiene copolymer of an arbitrary length and stretched to an arbitrary force at an arbitrary loading rate (because these parameters in sheared samples are unknown), we divided all backbone bonds of the styrene/butadiene copolymer into those formed during polymerisation (the "between" bonds) and those carried over from each monomer (the "within" bonds). To estimate the kinetics of mechanochemical fragmentation of an arbitrary "between" bond of a stretched chain, we calculated force-dependent activation free energies of fragmentation of the "between" bond in all 9 pairwise combinations of the 3 repeat units comprising the copolymer (1-phenylethanediyl, **1**, *trans*-but-2-en-1,4-diyl or 1,4-enchained butadiene, **2**, and but-3-en-1,2-diyl or 1,2-enchained butadiene, **3**, in Supplementary Fig. 17) (Supplementary Fig. 18). To confirm that the calculated activation barriers are not biased by the relatively close proximity of the scissile bond to the atom at which force acts, which is a direct result of using small molecules to mimic the behaviour of a long polymer chain,[14,15] we repeated the calculations for 2 longer homologues, (3*E*,7*E*,11*E*)-tetradeca-3,7,11-triene, **13** and 2,4,6,8-tetraphenyldecane, **14**. The force-dependent free energies were insignificantly different for both pairs of homologues (**10** vs **13** and **12** vs **14**), validating our choice of two-repeat unit segments to model macrochains.

The calculations of force-dependent activation energies followed the previously described[16] and theoretically[14,17] and experimentally[15,18-20] validated method. First, we performed an rPES on the longest conformer of each kinetically-significant stationary state, with the $_{Me}C{\cdots}C_{Me}$ distance increased stepwise to cover forces up to 6 nN (for fragmentation TSs, scans were performed with both increasing and decreasing distance, depending on the stretching force at which the initial TS was optimised). These scans yielded electronic energies, E and constrained distances q at several increasingly large values of the stretching force, f. Analytical frequency calculations were performed on a subset of the converged scan points to estimate force-dependent thermodynamic corrections, $TC(f)$. The data, which correspond to non-uniformly spaced force increments were interpolated to yield $E(f)$, $TC(f)$ and $q(f)$ at the same uniformly spaced values of f for all species. The force-dependent activation free energies were calculated as

$$\Delta G^{\ddagger}(f) = E^{\ddagger}(f) + TC^{\ddagger}(f) - f \times q^{\ddagger}(f) - [E^{R}(f) + TC^{R}(f) - f \times q^{R}(f)] \tag{5}$$

Because all mechanochemical reactions are unimolecular, their activation enthalpies and free energies were within 1 kcal/mol.

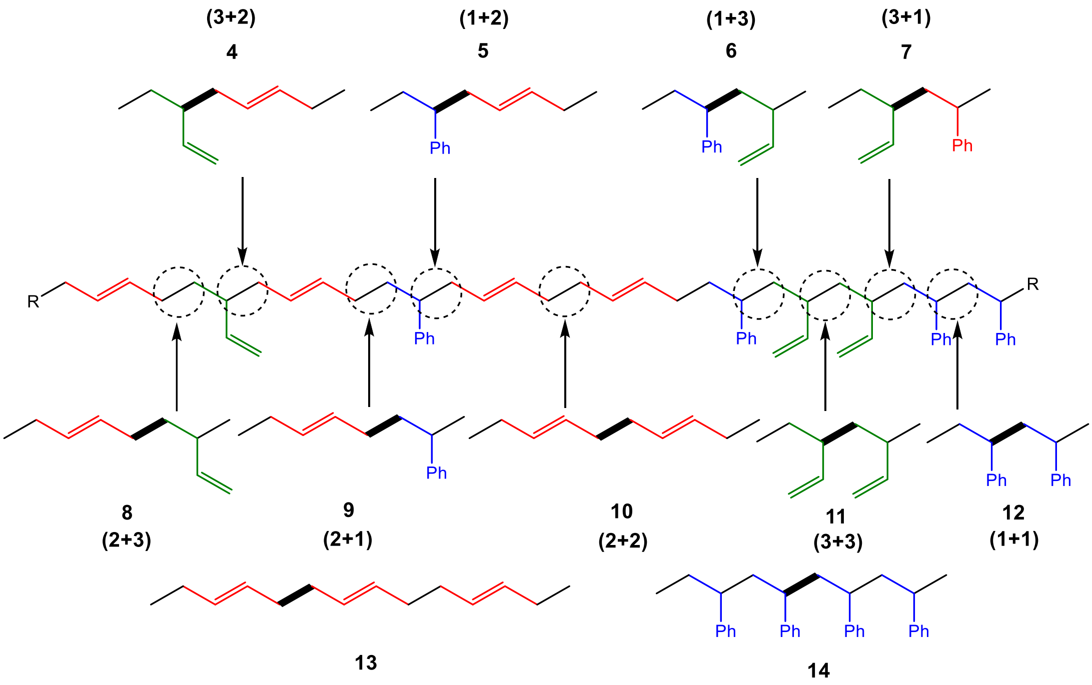

Supplementary Fig. 18 The mechanochemical kinetics of fragmentation of ''between'' bonds of a chain of the styrene/butadiene copolymer (i.e., bonds formed between repeat units, illustrated by circles, labelled as **B1-B9,** Supplementary Table 2) was modelled by computing the force-dependent activation free energies of homolysis, $\Delta G^{\ddagger}(f)$, of the bonds highlighted in bold in the 9 pairwise combinations of repeat units, **4**-**12**. We tested the effect of molecular size on computed $\Delta G^{\ddagger}(f)$ by calculating $\Delta G^{\ddagger}(f)$ for homolysis of a "between" bond (bold) in 2 larger homologues, **13** and **14**, of two-repeat-unit segments, **10** and **12**, to estimate the effect of molecular size on computed force-dependent activation free energies of fragmentation.

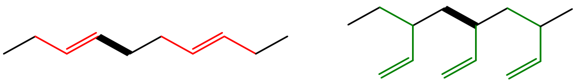

15 **16**

Supplementary Fig. 19 Segments for calculations of the free energy of fragmentation of the ''within'' bonds, i.e., bonds within the repeat units (**B10**-**B11**, Supplementary Table 2). To model the scission kinetics of "within" bonds, we calculated force-dependent activation barriers of fragmentation of (3*E*,7*E*)-deca-3,7-diene, **15** (**B10**, Supplementary Table 2) and (5*R*)-3-ethyl-7-methyl-5-vinylnona-1,8-diene, **16** (**B11,**Supplementary Table 2) (Supplementary Fig. 19). The fairly large size of the tetraphenyl derivative, **14**, means that the two backbone bonds connecting the central benzylic C are very nearly equivalent (i.e., they can be classified equally as a "within" or a "between" bond) and we used $\Delta G^{\ddagger}(f)$ calculated for **14**, which is equal to $\Delta G^{\ddagger}(f)$ calculated for **12** (**B9,** Supplementary Table 2), as representative of the fragmentation kinetics of the "within" bond of the 1-phenylethanediyl repeat unit. All fragmentation reactions discussed above are summarised in Supplementary Table 2, while the fractions presented in the table were calculated based on the structure of the copolymer as described later.

Supplementary Table 2 The reactions used for computational model of chain fragmentation kinetics. Fractions do not add to 1 because of rounding. In calculations of fragmentation probabilities, the fractions of bonds used added to 1.

Bond	Segment	Model scission reaction	Fraction among *scissile* backbone bonds
B1	4	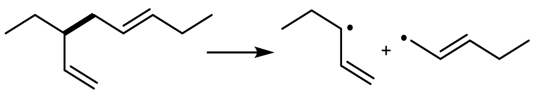	0.12
B2	5	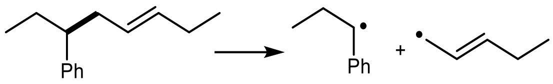	0.05
B3	6	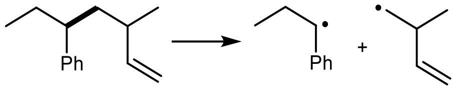	0.04
B4	7	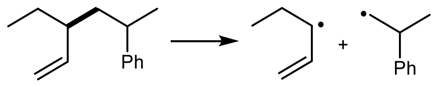	0.04
B5	8	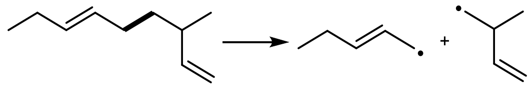	0.12
B6	9	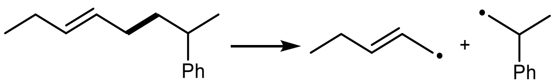	0.05
B7	10	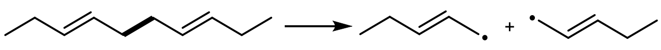	0.16
B8	11	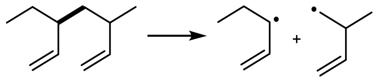	0.08
B9	12	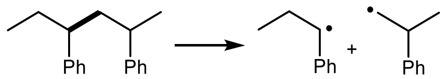	0.12[1]
B10	15	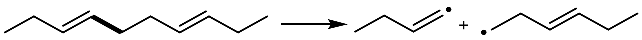	0[2]
B11	16	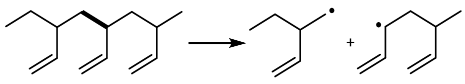	0.23[3]

1. Includes the fraction of the within bond of the styrene-derived repeat unit for all possible combinations of the flanking moieties.
2. Fracture of this bond is too slow to be competitive with other backbone bonds and it is considered non-scissile.
3. Includes all within bond of the 1,2-butadiene derived repeat unit, regardless of the flanking moieties, not just of the segment shown.

The calculations showed that the barrier for fragmentation of the "within" bond of the 1,4-enchained butadiene (but-2-en-1,4-diyl, **B10**, Supplementary Table 2), is at least 5 kcal/mol higher than the barrier for scission on any other bond. Consequently, homolysis of this bond is not kinetically competitive at any force, and it is excluded from further discussion. Likewise, we assumed that chain never fractures by dissociation of backbone C=C bonds. The next highest-energy barriers correspond to homolysis of the "between" bond in **9**-**10** (**B6**-**B7,** Supplementary Table 2**)**: these reactions contribute negligibly to the chain fragmentation kinetics. At force <3.4 nN, chains fracture primarily by dissociation of bond B1 or B2, and at higher forces, B9 bond is most labile.

The fragmentation kinetics of a chain that is long enough to assume statistical distribution of the repeat units and wherein each backbone bond experiences the same average stretching force (a situation that occurs for example when an isolated chain is stretched by pulling its terminal atoms away from each other as in single-molecule force experiments[21]) is governed by a composite rate constant (Eq. (6)), where χ_i is the fraction of the scissile backbone bonds with the fragmentation activation energy of $\Delta G_i^{\ddagger}(f)$, with each *i* representing one of the 11 unique scissile bonds (**B1-B11**) in Supplementary Table 2.

$$k = \frac{k_B T}{h} \sum_{i=1}^{11} \chi_i e^{-\frac{\Delta G_i^{\ddagger}(f)}{RT}} \tag{6}$$

The fragmentation kinetics of "within" bonds appears independent of the nature of the flanking repeat units. Consequently, the fraction of a within bond *i*, among the *scissile* backbone bonds of the copolymer chain, χ_i, is proportional to the molar fraction of each repeat unit, m_i, as

$$\chi_{i\,within} = \frac{m_i}{2 - m_2} \tag{7}$$

Where m_1, m_2 are mole fractions of 1-phenylethanediyl, **1**, and but-2-en-1,4-diyl, **2,** respectively (Supplementary Fig. 17). The denominator reflects the fact that each but-2-en-1,4-diyl repeat unit contributes 1 scissile backbone bonds (its single "between" bond, while homolysis of its 3 "within" is too slow to contribute to chain fragmentation), whereas the other repeat units contribute 2 scissile backbone bonds each. Note that Eq. (7) does not apply to 1,4-enchained butadiene (but-2-en-1,4-diyl, **2**), because it lacks scissile "within" bonds.

The activation free energy of homolysis of a "between" bond depends on the nature of the two repeat units, *p* and *q*, where *p*=1-3 and *q*=1-3, connected by the scissile bond and the order in which they are connected (head-to-tail or tail-to-head). The fraction of segment *pq*, $\chi_{pq} = \chi_{qp}$, is determined by the product of the molar fractions of the two repeat units, which reflects the probability that two repeat units are bound to each other in either order as

$$\chi_{i\,between} = \chi_{pq} = \frac{(2 - \delta_{pq}) m_p m_q}{2 - m_2} \tag{8}$$

Where δ_{pq} is Kronicker's δ function.

This data allows the number of alkyl, aR$^\bullet$, and stabilized (allyl/benzylic), sR$^\bullet$, radicals produced by fragmentation of an average chain to be calculated as a function of fracture force or half-life, $\tau_{1/2}$, of the stretched chain (Supplementary Fig. 20). A chain cannot remain stretched (much less to the very high force required for homolysis of C-C bond at 10 °C) for longer than either the terminal relaxation time or the residence time of the chain in the capillary of the flow cell. The latter is 1.6 s based on the constant volumetric flow rate maintained by the shearing apparatus; we estimated the former by scaling the reported terminal relaxation time for a styrene/butadiene copolymer of very similar composition to the one used in our study (2 s) by the ratio of M_w of our sample to that of the literature one (165 vs. 130 kDa) and the corresponding zero-shear viscosities (7 MPa for our polymer at 10 °C vs. ~2 MPa for the literature polymer).

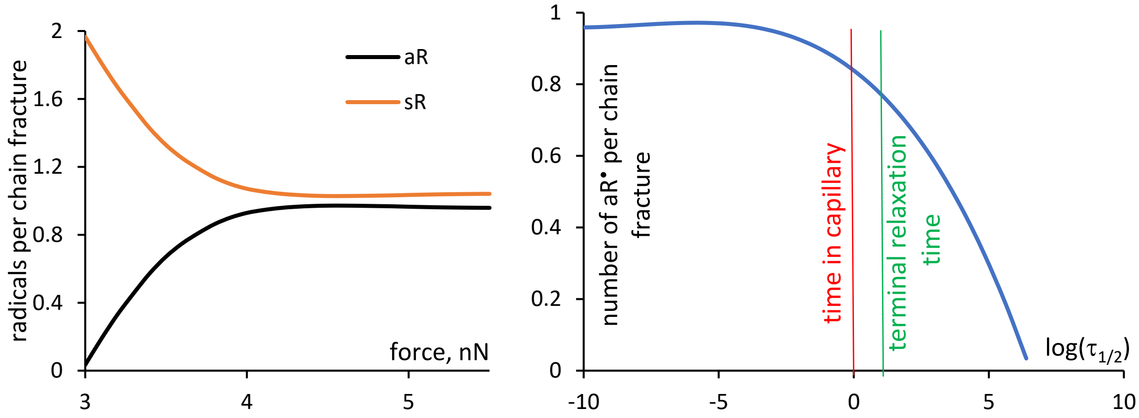

Supplementary Fig. 20 Calculated stoichiometry of chain fracture, expressed as the number of the specified macroradicals per chain fracture as a function of force at break or the half-life of the stretched chain. The distribution of half-lives of fragmenting chains in a loaded material is unknown but they should be (much) less than either the time that material is loaded or the terminal relaxation time, indicated by vertical graphs. This allows the plausible average stoichiometry of chain fracture in sheared material to be estimated at >0.8 aR$^{\bullet}$ + <1.2 sR$^{\bullet}$.

Kinetics and structure-reactivity relationships of macroradical reactions.

Our computations summarised below suggest that the full range of reactivities of the macroradicals produced by fragmentation of a chain of the styrene/butadiene copolymer is adequately captured by two species: a simple alkyl radical, aR$^{\bullet}$, and a stabilized radical, sR$^{\bullet}$, which includes all allylic and benzylic radicals.

Supplementary Table 3 The effect of the functional on the calculated activation enthalpies of key reactions underlying remodeling. All calculations were at the UHF formalism with 6-31+G(d) basis set in vacuum.

	Reaction	$\Delta H^{\ddagger}$ (kcal/mol)						
		APFD	BMK	CAM-B3LYP	M06-2X	wB97xD	MPW1K	exp
aR$^{\bullet}$	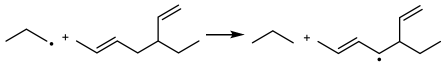	3.5	9.5	10.3	7.2	5.6	12.1	10.3[22]
	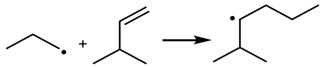	4.4	8.7	9.1	6.7	5.9	9.6	6.6[23]
sR$^{\bullet}$	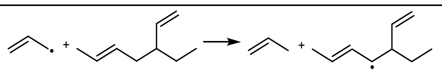	11.2	18.3	19.6	14.7	14.4	20.2	
	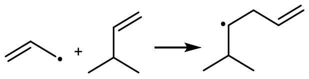	9.2	14.5	15.7	11.7	12.2	13.6	13.0[23]
ROO$^{\bullet}$	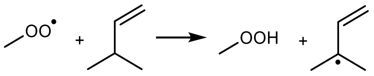	5.7	13.7	12.6	10.5	10.5	14.3	
	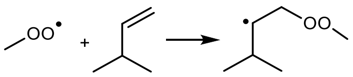	7.2	13.4	12.7	11.4	10.8	13.8	

The effect of substituents at the β-C (for alkyl and allyl radicals) or α-C (for ROO$^{\bullet}$) on computed activation and reaction enthalpies, $\Delta H^{\ddagger}$ or ΔH^{o}, is small (Supplementary Table 4 and first two columns of Supplementary Table 5), including affinity for O_2 (Supplementary Table 6). Alkyl radicals manifest modest but statistically significant, selectivity for addition to an unsubstituted olefinic C over substituted one (or aromatic C, $\Delta\Delta H^{\ddagger}$ 2.4 and 3.9 kcal/mol, respectively, Supplementary Table 4), for abstraction of allylic vs. alkyl or benzylic H atoms ($\Delta\Delta H^{\ddagger}$ = 3.6 kcal/mol, Supplementary Table 7), and for addition over abstraction ($\Delta H^{\ddagger}_{abs}$ > $\Delta H^{\ddagger}_{add}$ by 0.7 kcal/mol across 6 functionals in Supplementary Table 3). Conversely, ROO$^{\bullet}$ radicals manifest a

smaller preference for H atom abstraction ($\Delta H^{\ddagger}_{abs} < \Delta H^{\ddagger}_{add}$ by 0.3 kcal/mol across 6 functionals in Supplementary Table 3; see also Supplementary Table 5).

Supplementary Table 4 The activation/reaction enthalpies (in kcal/mol) for addition of alkyl or stabilized radicals to a side or backbone C=C bond at uMPW1K/6-31+G(d,p) level. See Supplementary Table 11 for additional data.

olefin		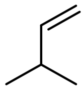		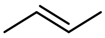	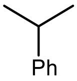
Product / Radical, R•		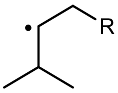	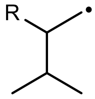	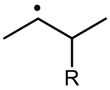	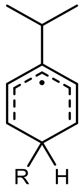
aR•	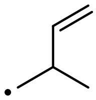	9.4/-25.4	11.4/-19.2	11.0/-20.4	12.6/-10.8
	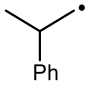	8.9/-24.9	11.3/-18.7		
	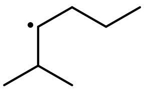	9.2/-21.8	11.1/-22.1		
sR•	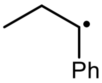	13.1/-9.9		15.5/-6.4	
	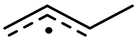	13.7/-9.7[a]<br>15.6/-7.9[b]<br>14.7/-4.8[c]		14.5/-7.2[d]	19.7/9.3

[(a-c)] Enthalpies for the production of 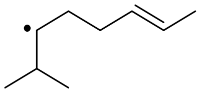 (a); 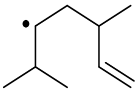 (b); 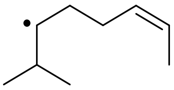 (c).

[(d)] for 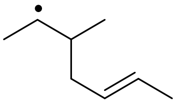

Supplementary Table 5 Calculated activation/reaction enthalpies (in kcal/mol) for addition of diverse $RO_2^\bullet$ to an sp^2 C atom or abstraction of an allylic H atom at uMPW1K/6-31+G(d,p) level. See Supplementary Table 11 for activation enthalpies of decomposition of β-peroxy radicals.

reactant	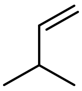		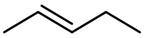		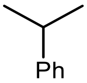
Product / $RO_2^\bullet$	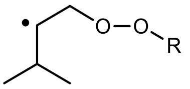	ROOH + 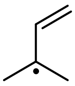	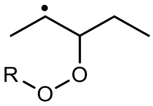	ROOH + 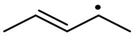	ROOH+ 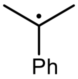
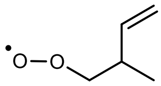	14.0/-2.9	14.8/-1.5	14.5/-3.9	15.0/0.3	12.9/0.2
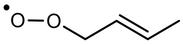	13.9/-3.0	15.6/-1.6			
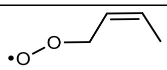	13.8/-3.1	14.6/-1.7			
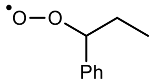	13.9/-3.5	15.9/-2.3			

Supplementary Table 6 Standard free energy of barrierless binding of O_2 to alkyl and stabilised radicals (in kcal/mol) at uMPW1K/6-31+G(d,p) level in vacuum. A tertiary allyl radical (last entry) is accessible only by H-atom abstraction from an intact chain by an alkyl radical. All other radicals are products of mechanochemical chain scission. When O_2 addition produces multiple isomers, the most favourable reaction energy is shown. Experimental values are -32.1 and -17.0 kcal/mol for O_2 binding to ethyl[24] and allyl radicals, [25] respectively.

Alkyl radicals, $aR^\bullet$						Stabilised radicals, $sR^\bullet$		
Major species				Minor species				
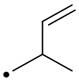	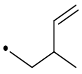	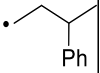	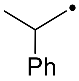	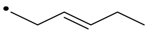	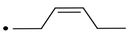	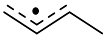	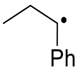	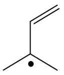
-28.3	-27.4	-27.4	-28.1	-28.5	-28.6	-13.1	-16.2	-11.7

Supplementary Table 7 The activation/reaction enthalpies (in kcal/mol) of H abstraction by an alkyl and stabilized radicals at uMPW1K/6-31+G(d,p) level.

H atom donor	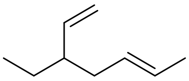				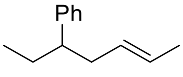
Product / reactant	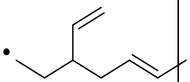	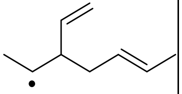	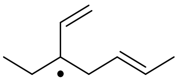	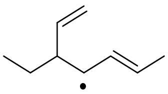	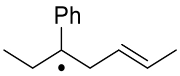
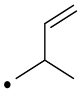 $aR^\bullet$	15.9/0.9	16.6/-0.9	12.9/-20.0	12.3/-18.2	14.2/-14.7

Intramolecular reactions of aR•, which include cyclisations and 1,n-H transfers, are highly sensitive to substituents multiple atoms away from the C radical (Supplementary Table 8). Such reactions do not affect the microstructure, mass distribution or type of the macroradical, and hence are not yet experimentally detectable. Their probable role in the remodeling mechanism is discussed in the main text.

Supplementary Table 8 Reaction, Δ*H*°, and activation, Δ*H*‡, enthalpies of intramolecular reactions of alkyl radicals.

Reactions	Δ*H*°, kcal/mol	Δ*H*‡,kcal/mol
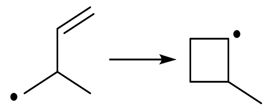	-1.9	N/A
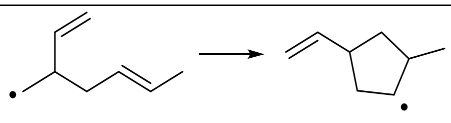	-21.3	16.4
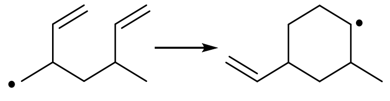	-30.5	9.3
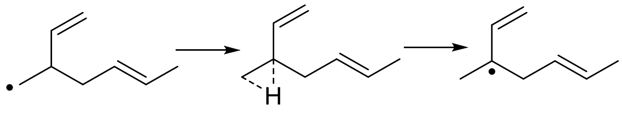	-20.4	33.2
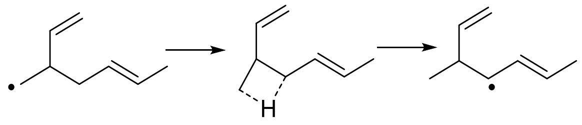	-17.7	31.4
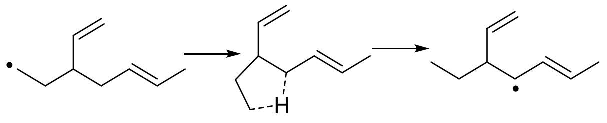	-19.1	14.3
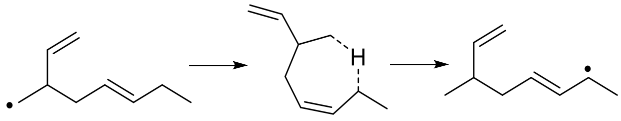	-18.8	14.5

Taking into account the concentrations of various types of C and H atoms in the copolymer (Supplementary Table 9), which changes negligibly during the reaction and using ΔΔ*H*‡ averaged over the 6 functionals in Supplementary Table 3 we estimated relative pseudo-unimolecular rate constants for various reactions of aR•, sR• and ROO• radicals using the total rate constant for addition of aR• to olefinic C atoms as 1. For aerobic shearing we used pseudo-1st order diffusion-limited rate constant for addition of O_2 to a macroradical of $3.2x10^3$ s^{-1}, based on the reported diffusion coefficient of O_2 in neat copolymer at 10 °C of $7.5x10^{-9}$ cm^2/s and the steady-state concentration of 1.5 mM (according to the formula: 0.0042exp(-277/T) M/bar, where T is the absolute temperature)[26]. Our assumption that the O_2 concentration in the sheared material remains constant despite continuous peroxidation of the melt is based on the observation of facile diffusion of O_2 into sheared melt saturated in N_2 prior to shearing in air.

Supplementary Table 9 Concentration (M) of reactive functional moieties in the styrene/butadiene copolymer used in our studies. See Supplementary Fig. 21 for the definition of the different types of C and H atoms.

Backbone C	sp^2 C				Allylic H		Benzylic H
	Total	2°	1°	aromatic	Total	3°	
60	41	16	5.5	15	37	5.5	2.5

Formulas used to calculate the values: Total concentration of sp^2 C atoms: $(0.26*6/MW_s+0.74*2/MW_{bd})\rho$; concentration of secondary sp^2-C: $(0.3+0.44*2)\rho/MW_{bd}$; concentration of primary sp^2-C = concentration of tertiary

allylic H: 0.3ρ/MW_{bd}; concentration of aromatic carbons: 0.26*6ρ/MW_s; concentration of allylic H: 0.74*2ρ/MW_{bd}; concentration of aromatic H: 0.26*5ρ/MW_s; ρ is the density of the copolymer, MW_s and MW_{bd} are molar masses of styrene and butadiene.

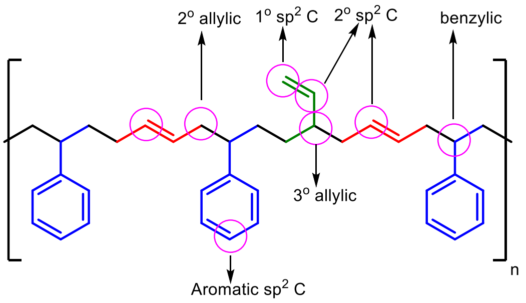

Supplementary Fig. 21. Different types of C and H atoms in the copolymer.

Supplementary Table 10 Estimated relative pseudo-1st order rate constant (s^{-1}) for reactions of aR•, sR• and ROO• macroradicals based on DFT calculations and the concentrations of various atoms in neat copolymer.

	Intermolecular addition to			Abstraction of allylic/benzylic H	All intramol. rxns
	Vinyl C	Aromatic C	O_2[(a)]		
aR•	1	<10^{-7}	0.01	0.06	0.02
sR•	8x10^{-4}	0	0.01	<10^{-7}	3x10^{-7}
ROO•	10^{-3}	0		2x10^{-3}	5x10^{-5}

[(a)] diffusion-limited: aerobic shearing only

Supplementary Table 11 Reaction, Δ*H*°, and activation, Δ*H*‡, enthalpies of key reactions between macroradicals and small-molecule solutes and related reactions.

	Δ*H*‡, kcal/mol	Δ*H*°, kcal/mol
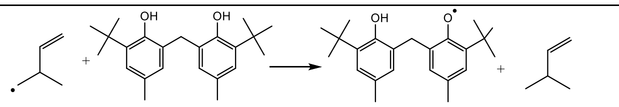	9.8	-21.5
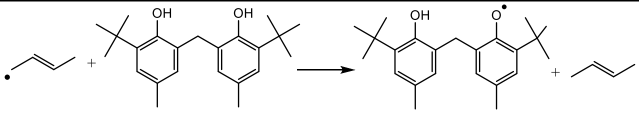	16.7	-6.8
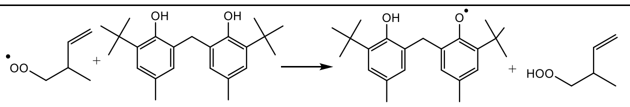	5.5	-2.2
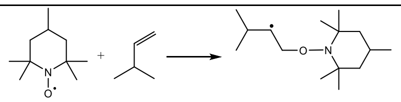	30.3	16.3
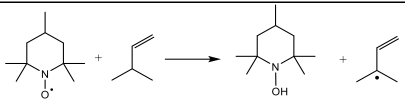	28.0	13.2
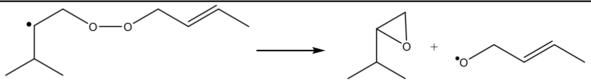	12.0	-29.5
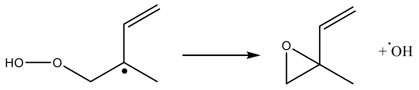	20.3	-7.7
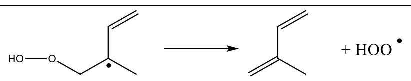	28.0	19.6

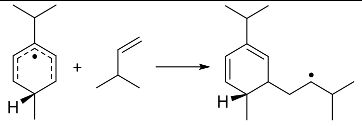	24.5	2.2

Supplementary Table 12 Calculated activation enthalpies, $\Delta H^{\ddagger}$ (kcal/mol) under standard conditions for shown recombination reactions. All results are in the gas phase with 6-31+G(d,p) basis set. Note that all calculated barriers are less than the effective $\Delta H^{\ddagger}$ for diffusion of each X in the melt.

Rxn	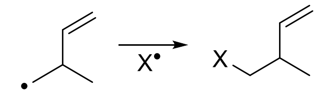			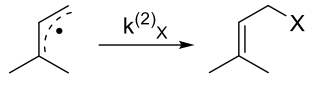			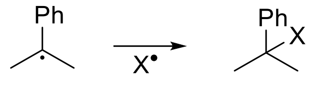		
X	O_2(1)	T• (2)	A•	O_2(3)	T•	A•	O_2(3)	T•	A•
uMPW1K	2.4	4.4	3.6	5.6	7.8	5.8	4.8	7.2	5.6
uB3LYP	0(4)	2.4	1.8	1.1	4.1	1.9	0.9	4.0	1.7

(1) Reported calculated $\Delta H^{\ddagger}$ in gas phase: 1.0 kcal/mol[25] or 1.5 kcal/mol[27]
(2) Experimental $\Delta H^{\ddagger}$ in solution for TEMPO + alkyl radicals are 0.9-2 kcal/mol[28]
(3) Reported range of $\Delta H^{\ddagger}$ in solution ranges from <2 kcal/mol to >5 kcal/mol, with the smallest value being the most recent.[29]
(4) purely dissociative curve.
We were unable to find any experimental or computational estimates of the activation barrier of recombination of any phenoxy radical with any C-centered radical.

Supplementary Table 13 Calculated activation energies, $\Delta G^{\ddagger}$ (kcal/mol) under standard conditions for β-scission of radicals at the uMPW1K/6-31+G(d,p) level in the gas phase.

aR•		sR•	
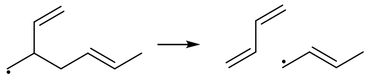	27.6	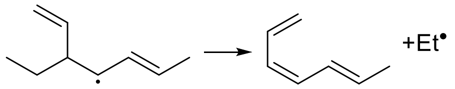	36.2
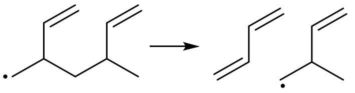	29.8	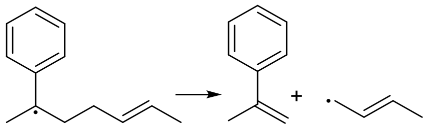	28.9
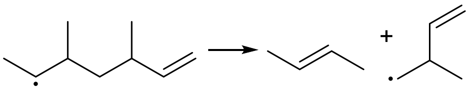	31.6		

Fracture of O-O bond in the backbone under mechanical load

Shearing air-saturated samples can be expected to generate chains containing peroxy, O-O, backbone bonds by recombination of ROO• and C-based macroradicals (Fig. 3, main text). When a segment of a chain containing such a bond is stretched, the chain may fracture by homolysis of the O-O bond rather than C-C bond, because the O-O bond is more labile than the C-C bond, although the selectivity decreases with force (Supplementary Fig. 22).

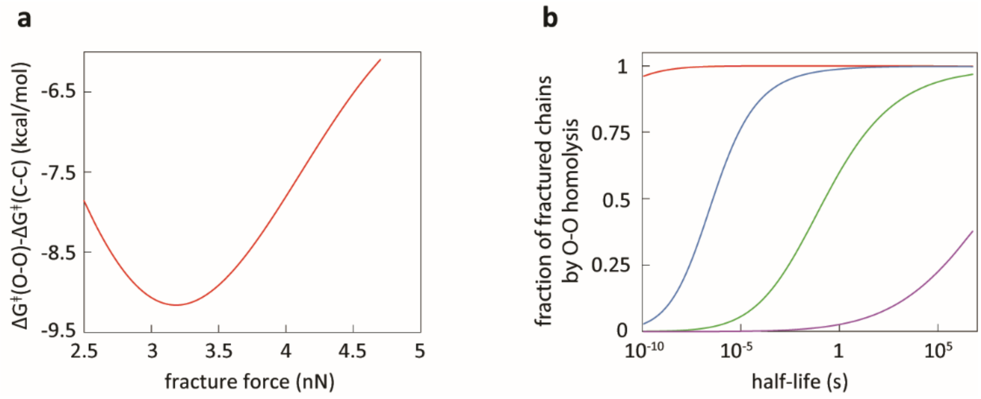

Supplementary Fig. 22 Computed effect of O-O backbone bonds on chain fracture. (a) Difference in the activation free energies of homolysis of the O-O bond and the effective C-C bond of the copolymer (Eq. (9)) as a function of the fracture force. The O-O bond does not exist at force >4.7 nN. (b) The fraction of overstretched chains of 1000 backbone bonds fracturing by homolysis of O-O bond vs. chain half-life. Chains contained 4 (purple), 10 (green), 20 (blue) and 100 (red) O-O bonds randomly distributed among the 1000 bonds; the remaining bonds were the average scissile backbone bond of the copolymer; the tensile force experienced by each backbone bond was assumed to decrease quadratically from the chain middle, reaching 0 at the termini.

$$\Delta G_{ef}^{\ddagger}(f) = -RT\ln\left(\sum_{i=1}^{11} \chi_i e^{-\frac{\Delta G_i^{\ddagger}(f)}{RT}}\right)$$

(9)

Estimated diffusion-limited rate constants

The high viscosity of the copolymer at 10 °C means that mass transport limits the rates of reactions between a small-molecule solute and a macroradical or between two macroradicals. The rate constant for reversible association of two freely diffusing species, A and B, into an encounter pair is given by (10))[30] which has been widely used in calculating of the diffusion-limited rate constant of bimolecular reactions.[31,32]

$$k_D = 4\pi\sigma N_A(D_A + D_B)$$

(10)

where σ is the "reaction distance", N_A is the Avogadro number, and D_A and D_B are the diffusion coefficients. No rigorous definition of the "reaction distance" exist, but it often is equated to the distance between the centres of mass of the two reacting moieties in the transition state.

We calculated the diffusion-limited rate constants for reactions between each small molecule solute and a macroradical by (11), where V_{O2} and V_S are the molecular volumes of O_2 (11.3 Å^3) and the solute, respectively, calculated quantum-mechanically, D_{O2} is the diffusion coefficient of O_2, given by (12).[26] (11) assumes that the diffusion of small molecules in the sample follows the Stokes diffusion law.

$$k_D = 4 \times 10^{-11}\pi\left(\sqrt[3]{0.75\pi V_S} + 3\right)N_A D_{O_2}\sqrt[3]{\frac{V_{O2}}{V_S}}\ M^{-1}s^{-1}$$

(11)

$$D_{O_2}(T) = 2.3 \times 10^{-3} e^{-\frac{3550}{T}}\ cm^2 s^{-1}$$

(12)

Supplementary Table 14 Estimated diffusion coefficients of $T^\bullet$ and AH and the diffusion-limited rate constants for reactions of O_2, $T^\bullet$ and AH with macroradicals in sheared copolymer at 10 °C based on DFT calculations. See Fig. 1 in main text for the structures of $T^\bullet$ and AH.

	O_2	$T^\bullet$	AH
V_s, Å^3	11.3	192	171
D, 10^{-9} cm^{2}s^{-1}	7.5*	2.9	3.0
k_D, 10^6 M^{-1}s^{-1}	2.1	0.5	0.4
Equivalent $\Delta H^\ddagger$, kcal/mol^	8.4	9.2	9.3

* from Eq. (12).
^ at 10 °C, assuming thermally-activated diffusion following the transition-state theory.[33]

Kinetic simulations based on the reaction mechanism

We simulated the remodeling of the copolymer predicted by the mechanism in Fig. 3 by representing every chain by a specific number of unbreakable inert repeat units, U, of 2.5 kDa each, connected by massless linkers each capable of linking an arbitrary number of U. All chemistry happens at a linker. The system is closed, of constant volume and spatially homogeneous, with no concentration gradients. The homogeneity of sheared samples was confirmed experimentally as described above. We ensured the finite size of the model without violating the conservation of mass by assuming that only chains whose concentration exceeds a threshold value react.

Fracture of a branched chain and addition of a macroradical to another chains create product chains of different topologies (see Supplementary Table 16-Supplementary Table 17 for examples). We manually defined the relationship between the distribution of product chain topologies for each reactant chain topology of up to 4 branch points, which allowed us to create product distributions programmatically (see next section). We described each chain by a size vector, which is a sequence of integers in specific order than uniquely define the size of each chain segment and their relative position in the molecule, and related to chain topology by systematic notation (Supplementary Table 15). A chain segment as a portion of a chain between two branch points or between a terminus and a branch point. Each number of the size vector is the number of U in each segment. A linear chain has a single segment and is coded by a single integer. An n-arm star chain is coded by a sequence of n integers in increasing order, each describing the size of each arm. See examples in Appendix 1.

In sheared material a chain of each structure is present in several chemically distinct forms, each comprising a unique component. For example, each linear chain, U_n, exists as a closed-shell macromolecule, and as a radical, with the unpaired electron at a terminus or at any of the ~n/2 unique linkers. Thus, the composition of a mixture is defined by a set of pairs of vectors, with each size vector paired with a concentration vector that specified the concentrations of various forms of the chain whose structure is defined by the size vector. The length and the structure of the concentration vector depend on the number of chemically-distinct species being tracked. For example, each linear chain in anaerobically-sheared neat copolymer and samples containing AH is characterized by a 4-element concentration vector, corresponding to the concentrations of the closed-shell form, a terminal alkyl radical, a terminal stabilized radical, and an internal radical. Because of the mechanism by which internal macroradicals were generated, they could only be stabilized and the unpaired electron was equally likely to localize between any pair of U, so that only the total concentration of internal macroradicals, rather than the concentration of each isomer of such macroradicals, need to be specified. In aerobically sheared material, the concentration vectors also include various isomers of $ROO^\bullet$ radicals and in sheared samples containing $T^\bullet$, the concentration vectors include the average number T_{poly} moieties in each chain at the same locations as the unpaired electron.

Supplementary Table 15 The notations of different chain topologies used in the code provided.

topol-ogy	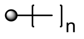	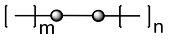	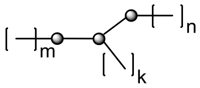	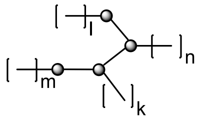	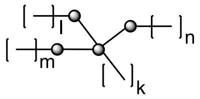
nota-tion	p_nx	p_mxpxp_n	$p_mxpxp_{k+1}xp_n$	$p_mxp_{k+1}xp_{n+1}xp_l$	$p_mxpxpxp_lp_{k+1}xp_n$

Distribution of product chain topologies

The chemical reaction for fracture of a linear chain U_n is:

$$U_n \rightarrow \sum_{i=1}^{n-1} a_i U_i \tag{13}$$

Where a_i is the stoichiometric coefficient for chain U_i:

$$a_i = 2\frac{e^{-\frac{\left(1-\frac{2i}{n}\right)^2}{\sigma}}}{\sum_{i=1}^{n-1} e^{-\frac{\left(1-\frac{2i}{n}\right)^2}{\sigma}}} \tag{14}$$

Where σ defines how fast the fragmentation probability decreases away from the chain center.

(13) applies to fracture of a branched chain if U_n is the overstretched segment. The generation of the product chains requires the knowledge of how branch points are distributed among the product chains, as illustrated in Supplementary Table 16. In this case a_i given by (14) are twice the probabilities of each reaction channel, except for fragmentation at multivalent linkers (i.e., branch points), where a_i are 4 times reaction channel probabilities. Reaction stoichiometries are obtained by consolidating identical chains resulting from different reaction channels.

We applied this approach to closed-shell chains with <13 segments. We approximated the products generated by mechanochemical fragmentation of larger chains by extrapolating and scaling product distributions derived from fracture of chains with 11 segments of the same topology.

We used a similar systematic combinatorial approach to generate distributions of products of addition of macroradicals with <13 segments to closed-shell chains (Supplementary Table 17). In this case, all reaction channels have identical probabilities. We assumed that any macroradical with >12 segments immediately converts to a closed-shell chain, which is equivalent to selective recombination of these macroradicals with the adventitious small-molecule radical scavenger.

Supplementary Table 16 An example of the method for determining the product distribution and stoichiometry of fragmentation of a branched chain, on example of a chain with 2 branch points. Fragmentation at a branched linker (branch point) usually generates two distinct products in equal amount, requiring further halving of corresponding α_i (14)). A vectorized version of this method is implemented in chainfracture.m file provided

		Products		Reaction channel probabilities
Reactant	Location of chain fracture	Chain structures	size-vectors[(a)]	
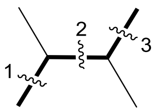<br>size vector: [5 **6 3** 6 **8**]<br>overstretched segment (bold): [6 3 8]<br>spanning length: 17	Anywhere between U1 and U5 (scenario 1)	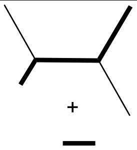	[1] + [5 5 3 6 8]<br>[2] + [5 4 3 6 8]<br>[3] + [5 3 3 6 8]<br>[4] + [5 2 3 6 8]<br>[5] + [5 1 3 6 8]	0.00917949702158470<br>0.0175118506797642<br>0.0304629457454259<br>0.0483212811158155<br>0.0698926769039515
	U5-U6	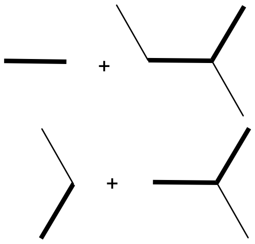	[6] + [8 6 8]<br>[10] + [4 6 8]	0.0460915809202469<br>0.0460915809202469
	Anywhere between U7 and U9 (scenario 2)	2 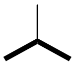	[5 6 1] + [2 6 8]<br>[5 6 2] + [1 6 8]	0.110865958822115<br>0.121582627870850
	U9-U10	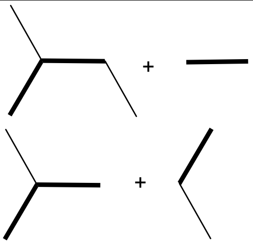	[5 6 3] + [14]<br>[5 6 10] + [8]	0.0607913139354248<br>0.0607913139354248
	Anywhere between U10 and U17 (scenario 3)	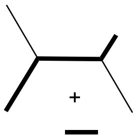	[5 6 3 6 1] + [7]<br>[5 6 3 6 2] + [6]<br>[5 6 3 6 3] + [5]<br>[5 6 3 6 4] + [4]<br>[5 6 3 6 5] + [3]<br>[5 6 3 6 6] + [2]<br>[5 6 3 6 7] + [1]	0.110865958822115<br>0.0921831618404937<br>0.0698926769039515<br>0.0483212811158155<br>0.0304629457454259<br>0.0175118506797642<br>0.00917949702158470

(a) The + sign signifies two components not algebraic sum.

Supplementary Table 17 An example of the method for generating size vectors of the products of addition of a macroradical to a closed-shell chain for a terminal macroradical (the unpaired electron is located at the terminus of the longest arm of 12 units, highlighted in bold in both the structure and the corresponding size vector). A vectorized version of this method is implemented in radicalgrowth.m file available at dx.doi.org/10.17638/datacat.liverpool.ac.uk/1697.

macroradical	Closed-shell chain	Location of addition	Products	Product size vectors
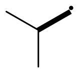<br>[5 9 **12**]	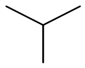<br>[3 5 6]	At terminus	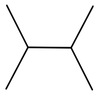	[5 9 15 5 8]<br>[5 9 17 3 8]<br>[5 9 18 3 5]
		At shortest arm<br>At middle arm<br>At longest arm	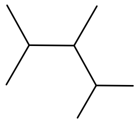	[5 9 12 1 2 5 6]<br>[5 9 12 2 1 5 6]<br>[5 9 12 1 4 3 6]<br>[5 9 12 2 3 3 6]<br>[5 9 12 3 2 3 6]<br>[5 9 12 4 1 3 6]<br>[5 9 12 5 1 3 5]<br>[5 9 12 4 2 3 5]<br>[5 9 12 3 3 3 5]<br>[5 9 12 2 4 3 5]<br>[5 9 12 1 5 3 5]
		At branch point	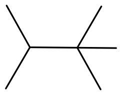	[5 9 12 3 5 6]

Code for generating product distributions.

Matlab functions chainfracture.m and radicalgrowth.m provided in the Supplementary Data file archive (doi: 10.17638/datacat.liverpool.ac.uk/1697) and the accompanying examples in examples.mat illustrate how we generated chains resulting from mechanochemical fracture of polymer chains or addition of macroradicals of various topologies to chains. The very many components comprising distributions of branched chains and multiple numbers that are needed to describe the structure and size of each branched chain require fairly large matrices to describe the polymer compositions in sufficient detail and large memory to manipulate these matrices. We ran our computations on HPC resources with at least 120 Gb of RAM and 500 Gb of scratch space; product mixtures for individual reactions can be generated with less memory, although it is impractical to use this code with <32 Gb of RAM except for the smallest chains. Because memory requirements scale with the number of segments of a chain as ~O(3) and ~O(4) for chainfracture.m and radicalgrowth.m, respectively, memory management is important. Memory requirements can be reduced, albeit at the expense of slower performance or degraded mass resolution and associated artifacts, with one of these strategies

1. Increase the mass of the effective repeat unit U: this was the strategy we used the most. The output of chainfracture.m is more sensitive to the number of U per chain than that of radicalgrowth.m and in either case results are unreliable for distributions containing fewer than 20 times the number of segments per chain. For HPCs with very fast I/O (e.g., at least SSD scratch), an optimal strategy is to increase the mass of U for input of radicalgrowth.m and reduce it for its output; the latter is achievable using the third function provided, reducebinsize.m.
2. Eliminate low-abundance chains, which is the least technically-demanding workaround.
3. Apply chainfracture or radicalgrowth sequentially to parts of reactant distributions instead of full distributions at once. This approach increases the time needed to generate full product distributions the most.
4. Reduce the size of each number used to define each chain, e.g., by using uint8 integers for sizes of segments and single-precision floating points for chain concentrations. This approach can lead

to large artifacts if the size of the product segment exceeds the maximum value supported by encoding (e.g., segment is larger than 255 units for uint8 definition).

Further details of how run the code is available by typing help [functionname] at Matlab prompt.

Macroscopic and microscopic rate constants

To make our simulations practical while explicitly tracking the evolution of 10^4-10^6 components (corresponding to chains of different mass and topology), we used a combination of rate constants that applied to chain of any size/structure and those that dependent on chain size/structure (Supplementary Table 18):

1. independent of the chain structure, which described radical recombinations (k_T, k_A, k_O for T•, A• and O_2 binding and k_r for macroradical recombination) or H abstraction from AH (k_{AH} and k'_{AH} for aR• and ROO•), and total rates of addition of a macroradical to another chain (k_a) and abstraction of H atom from another chain (k_h and k'_h); and
2. constants that are functions of either chain contour length (chain fracture, Eq. (15) below), or chain mass (addition of closed-shell chain *j*, to macroradical, $k_a(j)$, or H atom transfer from it to either aR• or ROO•, $k_h(j)$ and $k'_h(j)$, respectively, Eq. (16)).

Supplementary Table 18 The definitions of the rate constants used in the simulations and the corresponding model reactions used for DFT calculations of the reaction mechanism.

	Simulations	DFT calculations
k_a	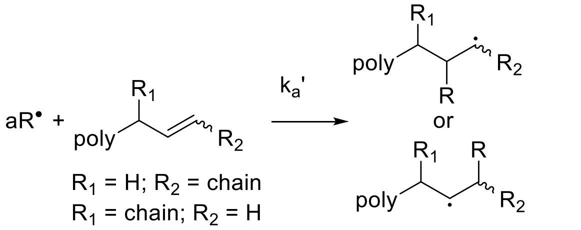 Pseudo-1st order rate constant:<br>$k_a = k_a'[sp^2C]$ (Supplementary Table 16)<br><br>Described the kinetics of consumption of aR• by addition.	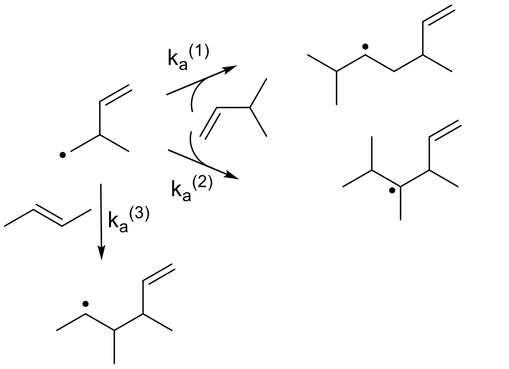 Pseudo-1st order rate constant:<br>$$k_a = \sum_{i=1}^{3} k_a^{(i)} C_i$$<br>Where C_i is the concentration of the corresponding type of sp²-C (Supplementary Table 9) and k_a(i) are calculated from ΔH in Supplementary Table 4.
k_h	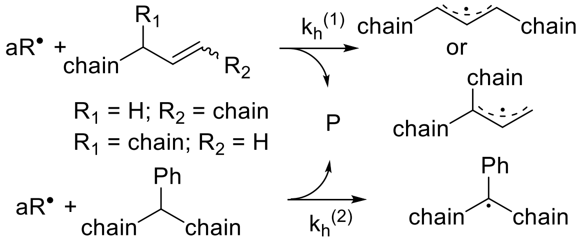 P is any close-shell macromolecule<br><br>Pseudo-1st order rate constant:<br>$k_h = k_h^{(1)}[H_{allylic}] + k_h^{(2)}[H_{benzylic}]$ (Supplementary Table 16);<br><br>Described the kinetics of consumption of aR• by H abstraction.	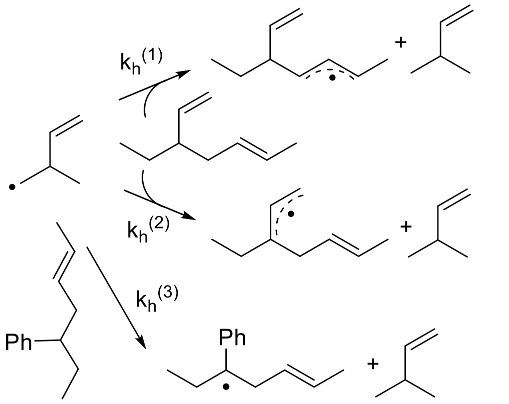 Pseudo-1st order rate constant:<br>$$k_h = \sum_{i=1}^{3} k_h^{(i)} C_i$$<br>Where C_i is the concentration of the corresponding type of H (Supplementary Table 9) and k_h(i) are calculated from ΔH in Supplementary Table 7.

k_h'	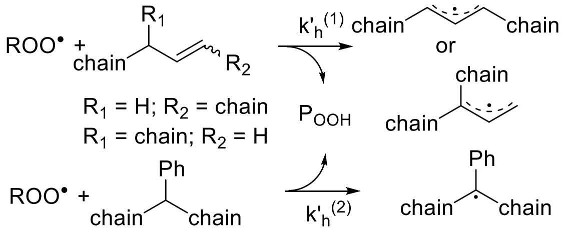<br>P is any close-shell macromolecule carrying an OOH group<br><br>Pseudo-1st order rate constant:<br>$k'_h = k'^{(1)}_h[H_{allylic}] + k'^{(2)}_h[H_{benzylic}]$ (Supplementary Table 16);<br><br>Described the kinetics of consumption of ROO• by H abstraction.	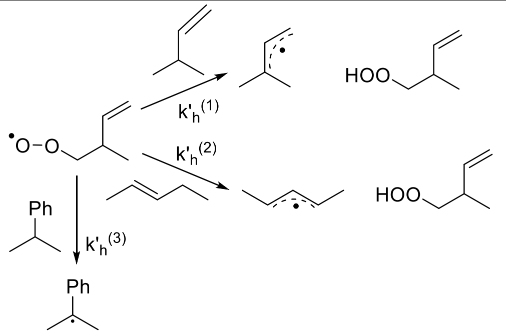<br>Pseudo-1st order rate constant:<br>$$k'_h = \sum_{i=1}^{3} k'^{(i)}_h C_i$$<br>Where C_i is the bulk concentration of the corresponding type of H (Supplementary Table 9) and $k'_h(i)$ are calculated from ΔH in Supplementary Table 5.
k_T,<br>k_O,<br>k_A,<br>k_r,	$R^\bullet + X^\bullet \xrightarrow{kX'} RX$; X = T•, A• or O_2<br>Pseudo-first order rate constants: (X = O_2 or T•):<br>$k_X = k_X'[X]_{bulk}$;<br>Bimolecular rate constant (X = A): $k_A = k_A'$<br>$R^\bullet + R'^\bullet \xrightarrow{kR} RX$<br><br>Described the kinetics of consumption of aR• or sR• by recombination.	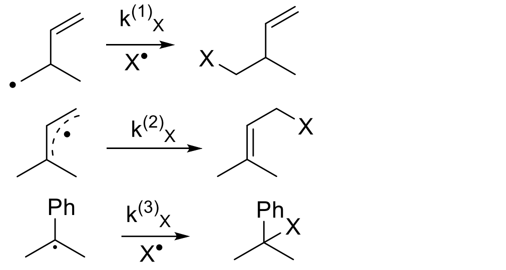<br>Pseudo-first order rate constants (X = O_2 or T•):<br>$$k_X = [X]_{bulk} \sum_{i=1}^{3} \frac{1}{\frac{1}{k_X^{(i)}} + \frac{1}{k_X^d}}$$<br>Bimolecular rate constant (X = A):<br>$$\frac{1}{k_A} = \left(\frac{1}{k'_A} + \frac{1}{k_A^d}\right)$$<br>Where $k_X(i)$ are calculated from ΔH in Supplementary Table 12 and k_X^d are the diffusion-limited rate constants (Supplementary Table 14)
k_{AH}<br>,<br>k'_{AH}	aR + AH $\xrightarrow{kAH}$ P + A<br>ROO + AH $\xrightarrow{kbAH}$ P_{OOH} + A<br>Pseudo-1st order rate constants:<br>$k_{AH} = k^{(b)}_{AH}[AH]_{bulk}$, $k'_{AH} = k'^{(b)}_{AH}[AH]_{bulk}$<br><br>Described the kinetics of consumption of aR• or ROO• by H abstraction from AH.	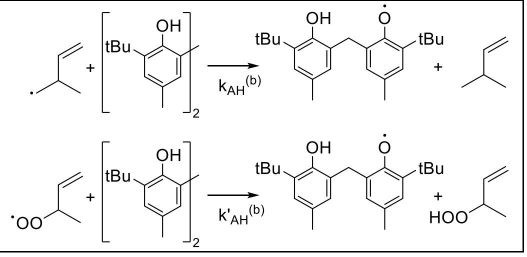

Chain-invariant rate constants, k_T, k_O, k_a, k_h and k'_h were pseudo-first order and therefore included time-independent multipliers corresponding to constant concentrations of T•, O_2, olefinic C atoms and abstractable H atoms, respectively. Chain-invariant rate constants k_A and k_r were 2nd order. Because our simulations were performed in normalized time, k_T, k_O, k_a, k_h and k'_h were unitless and k_A and k_r had the units of M^{-1}. The rate constant for fragmentation of chain *j*, $k_f(j) = (ecl(j)/63)^m$, where *ecl*(*j*) is the effective contour length of chain *j* (15)), sl_i is a spanning length of the chain and summation is over all unique spanning

lengths; for a linear chain the contour and spanning length are the same); m is a fitting parameter describing the scaling of the rate constant with chain size and 63 is the number of U in the reference chain.

$$ecl = \left(\sum_{i=1}^{N} sl_i^m\right)^{1/m} \quad (15)$$

The rate constants describing the reaction of chain U_j with a macroradical, $k_X(j)$, is

$$k_X(j) = k_X \frac{j}{\sum iU_i} \quad (16)$$

Where k_X is k_a, k_h or k'_h, depending on the reaction and the macroradical and summation is over all components in the sample.
Finally, we assumed that k_r and k_X of macroradicals with the unpaired electron at a branch point with valency, v >5, are proportional v-1, i.e., as the steric bulk around the C bearing the unpaired electron increases, the macroradical becomes less reactive toward addition to another chain or abstracting H atom from another chain, but its reactivity toward small radicals remains constant. A similar effect of steric bulk at the C bearing the unpaired electron on the kinetics of some macroradical reactions was suggested previously.[34]

To avoid expensive mathematical operations on chains that are present in such low concentrations that the concentrations of the products of their fragmentations or addition/H abstraction will be below the numerical accuracy of the code we assumed that at any time step a chain with concentration $<10^{-13}(ecl(i)/(1+\sigma))^{m-1}$ didn't fragment and a chain with concentration $<10^{-12}f_m(i)$ was inert in addition or H atom transfer reactions. In these formulas, $ecl(i)$ is the effective contour length of chain i (15)), m and σ quantify the sample and chain-level distributions of fragmentation probabilities as described above and in the main text, and $f_m(i)$ is the mass fraction of chain i in the sample. The formulas take into account that (a) the fraction of chains that fragment over a finite time step increases with their effective contour length ($ecl(i)$, (15)) but larger chains produce more fragments so that the concentration of each daughter chain is proportionally lower; and (b) the broader the distribution of fragmentation probabilities (σ), the more fragments are produced. The cutoff values for addition and H abstraction reflect the fact that the probability of a chain to donate a H atom or to add to a macroradical is proportional to the size of the chain. In simulations <0.1% of the chains would meet the above threshold. Accumulations of chains whose reactions were neglected in one step resulted in them being included in reactions in subsequent steps.

Fitting strategy

We validated mechanism in Fig. 3 (main text) by identifying a single set of normalized rate constants (listed in the Rate constants section), m, σ and contraction factors which reproduced change in aM_w vs. concentration of polymer-bound T or OOH moieties, $[X_{poly}]$, or change in aM_w vs. cycle (anaerobic shearing of neat and AH-doped samples only). Because of the length of each simulation to cover experimentally observed degrees of remodeling, we used the response-surface theory rather than the more conventional non-linear LSF algorithms (e.g., trust region reflective or Levenberg-Marquardt) for identifying the rate constants. We used the standard non-linear LSF algorithm as implemented in the lsqnonlin function of Matlab to optimize the contraction factors.

We started with simulations of remodeling of samples containing $T^\bullet$ at 100 mM, because these were the least demanding computations. We first established that for k_a>14.5, the apparent MD depended only weakly on the value of k_a, but strongly on the ratios k_h/k_a and k_T/k_a. This observation can be rationalized by noting that increasing k_a while maintaining constant k_h/k_a, k_r/k_a and k_T/k_a ratios only changes the fraction of primary macroradicals (i.e., products of chain fracture) that react over the time step, without affecting the relative contributions of different decay paths to the overall consumption rate of such radicals. Because at k_a>14.5, the steady-state concentration of primary radicals is already <3% of all macroradicals (depending on the values of k_h, k_r and k_T), reducing it further makes little difference on MDs.

Consequently, in our search for the parameter set we fixed k_a at 15 and only varied the ratios of the rate constants by sequential application of the central design. For each simulation, a full complement of the contraction factors was optimized by the standard non-linear LSF as described in the next section to enable comparisons to measured aM_w vs. $[X_{poly}]$ trends.

The rate constants for the initial cycle of parameter optimization was generated with the parameter limits of [0, 1], [1, 10^4], [0.1, 100], [1, 3], [0.1, 0.5] for k_h/k_a, k_r/k_a, k_T/k_a, m and σ, respectively and allowed us to constrain k_h/k_a <1 and $0.25 < \sigma < 0.35$; k_T/k_a and m were moderately correlated, yielding pairs of plausible values instead of individual limits; the dependences of aM_w vs. $[T_{poly}]$ on the k_r/k_a ratio were too weak to narrow the initial range. The second cycle using the newly constrained limits was applied to the sample $T^\bullet$-doped at 0.01 M and allowed us to constrain k_h/k_a <= 0.1, k_r/k_a <1500, $1<k_T/k_a<3$ (for total $T^\bullet$ concentration of 10 mM) and $2.2 < m < 2.6$.

Because k_h/k_a, k_r/k_a, k_{AH}/k_a and k_A/k_a could only be constrained on simulations of anaerobic shearing of neat or AH-doped polymer, and for such experiments only chain masses are both measured and simulated, we also needed a relationship between the simulation reaction time (which is expressed as a multiple of reference fragmentation rate constant, k_f^{ref}) and the experimental reaction time (which is expressed as the number of cycles). For each simulation of anaerobically sheared neat and AH-doped samples we performed non-linear LSF to find the best value of the conversion factor between simulated and experimental timescales.

The final set of values were (see below for the explanation of the uncertainty limits): k_a = 15, k_h = 1.0, k_T = (2600±300)$[T]_{bulk}$, k_{AH} = 170$[AH]_{bulk}$, k_A = 270 M^{-1}, k_r = 6800 M^{-1}, k_O = (18000±2000)$[O_2]_{bulk}$, k'_h = 150±30, k'_{AH} = (1800±600)$[AH]_{bulk}$, m =2.5±0.3, σ=0.3±0.1 and k_X = 0.75 (k_X is the rate constant for the background macroradical scavenging rate which is independent of the concentration of $T^\bullet$, AH or O_2); [T], [AH] and $[O_2]$ are in M. The best match between simulated and experimental aM_w vs. cycle correlations with these parameters for anaerobically sheared neat and AH-containing material required k_f^{ref} = 5.8×10^{-4} and 4.6×10^{-4} $cycle^{-1}$, respectively.

Estimates of the contraction factors

We are not aware of any published values of Mark-Houwink parameters or contraction factors for branched styrene/butadiene copolymers. The closest relevant literature data we could find is on polyisoprenes,[35] but the data is limited and contradictory. We measured the contraction factors for well-defined regular 3-arm and 4-arm star polystyrenes and an H-polystyrene. We prepared the star polymers by linking low-dispersity mono-CO_2H-terminated polystyrenes with M_n = 12.8, 26.1 and 48.0 kDa to either tris(3-hydroxypropyl) benzene-1,3,5-tricarboxylate or butane-1,4-diyl bis(3,5-bis(3-hydroxypropoxy)benzoate), to obtain 3-arm or 4-arm star polymers, respectively. The H polymer was obtained by esterifying a low-dispersity bis-CO_2H-terminated polystyrene with M_n=24.8 kDa with tris(3-hydroxypropyl) benzene-1,3,5-tricarboxylate and attaching mono-CO_2H-terminated polystyrenes with M_n = 12.8, 26.1 and 48.0 kDa to all 4 terminal OH groups. The knowledge of the accurate mass distributions of the linear reactant(s) allowed us to calculate the true mass distribution of the branched product. By comparing the latter to the measured apparent MDs we determined the contraction factors without relying on any assumptions. These were 0.88±0.02, 0.83±0.03 and 0.78±0.04, for 3-arm, 4-arm and H polymers, respectively and were independent of the chain mass, as expected.[36] Contraction factors of all other microstructures were estimated using the formula $g'=1+aN_{seg}+bN_{bp}$, where N_{seg} and N_{bp} is the number of segments and number of branch points and a and b are coefficients that were optimized to maximize the fits to experimental data. Across the whole data, a = -0.041±0.004 and b= -.0024±0.0007. The errors reflect the fact that the dependence of the fitted contraction factors on N_{seg} and N_{bp} is more complex than the equation above, which estimates the converged contraction factors to within ±15%.

We used these contraction factors to convert calculated true MMDs and $[T_{poly}]$/[styrene] vs. chain mass dependences to apparent MMDs (Supplementary Fig. 25), apparent differential MMDs (Supplementary Fig. 26) and $[T_{poly}]$/[styrene] vs. apparent chain mass (Supplementary Fig. 27) for comparison with experiments.

Estimates of the confidence intervals on fitting parameters

We derived the errors on m, σ, k_T/k_a from the Jacobian for aM_w vs. $[T_{poly}]$ estimated by (a) carrying out a series of simulations of T-doped copolymer (0.01 and 0.1 M) with the final set of rate constants, m, σ and contraction coefficients, except that in each simulation one of m, σ, or k_T was changed systematically to each of these values: m = 2.4 or 2.6, σ = 0.25 or 0.35, k_T = 2700[T] or 2400[T], (b) for each simulation calculating aM_w for each value of experimental value of $[T_{poly}]$ and (c) approximating the corresponding local value of the Jacobian by the ratio of the difference of aM_w for the standard set of parameters and each modified value divided by the difference of these parameter values. This approach approximates the calculations of Jacobians in conventional non-linear least-squares fits of a multivariate model. All other parameters were assumed to be fixed and contribute nothing to residuals. The Jacobian was converted to the confidence intervals using Eq. (3)-(4). We used the same approach to estimate the errors on k_O and k'_{AH} from simulations of aerobically sheared AH-doped (0.2 M) sample. Because extending such simulations to other experiments required unaffordable amount of computational resources and aM_w vs. $[X_{poly}]$ correlations in simulations of all other experiments were only weakly sensitive to the values of other rate constants (e.g., k_h), we were unable to estimate their confidence intervals. It seems plausible that the relative errors on the other rate constants are similar.

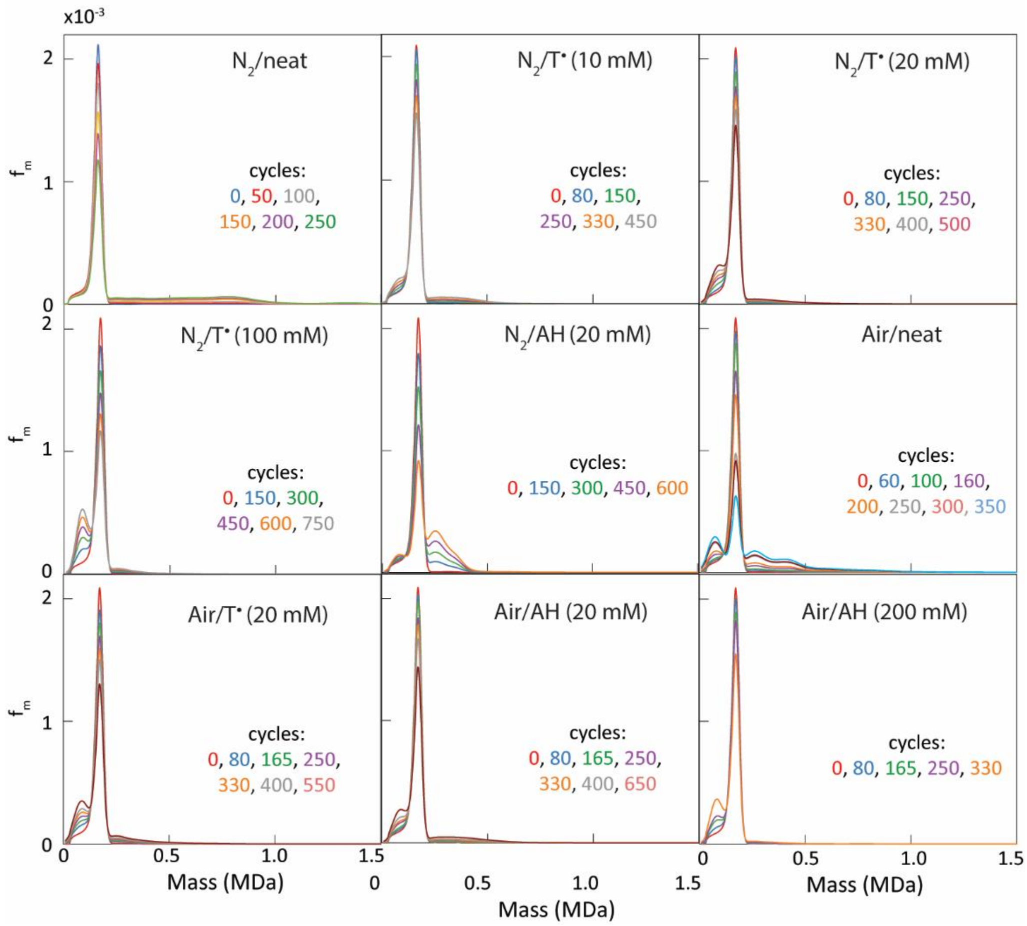

Supplementary Fig. 23 Simulated molar mass distributions (MMDs) whose aM_w most closely match corresponding measured values for each shearing condition; f_m is the relative mass of each 100 Da fraction.

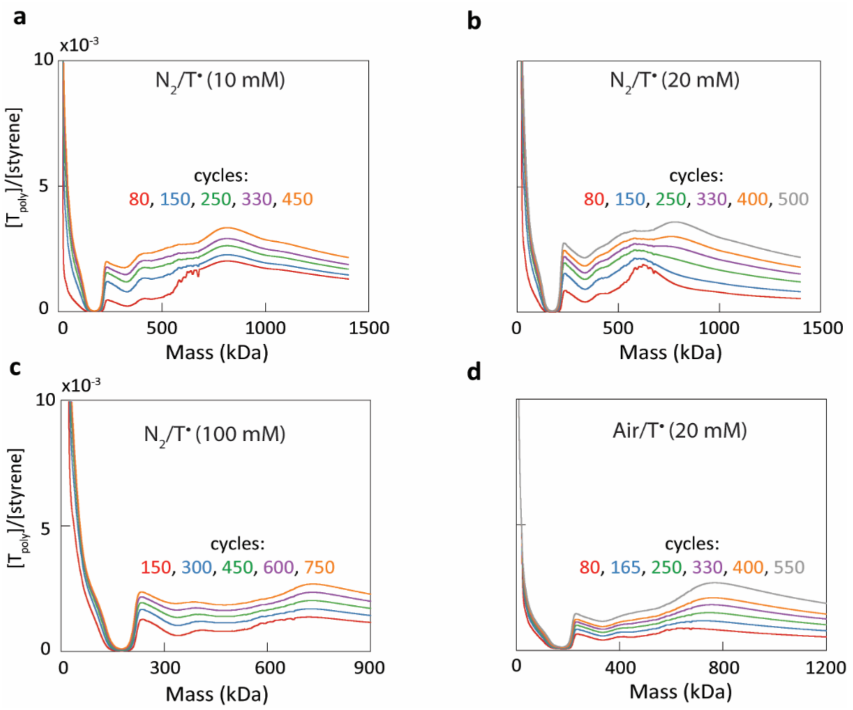

Supplementary Fig. 24 Simulated [T_{poly}]/[styrene] ratios for T-doped samples sheared in N_2 (a-c) or air (d). In aerobically-sheared samples (d), ~39% of consumed T• was in the form of the ROOT salt; this T is not included in the [T_{poly}]/[styrene] ratios because we assumed that these salts hydrolyzed immediately on exposing the THF solutions of sheared samples to air and thus not measured in SEC.

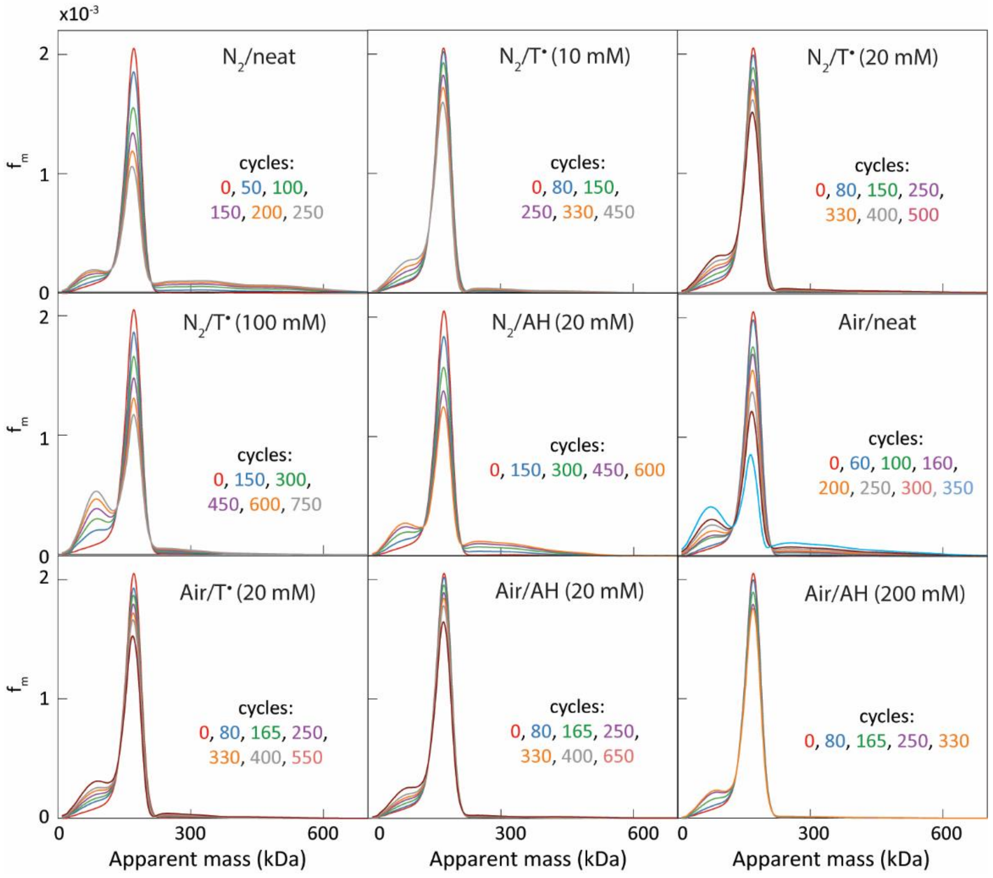

Supplementary Fig. 25 Simulated apparent molar mass distributions (aMMDs) that most closely match each measured aM_w for each shearing condition; f_m is the mass fraction of all chains in each 100 Da fraction of apparent chain masses.

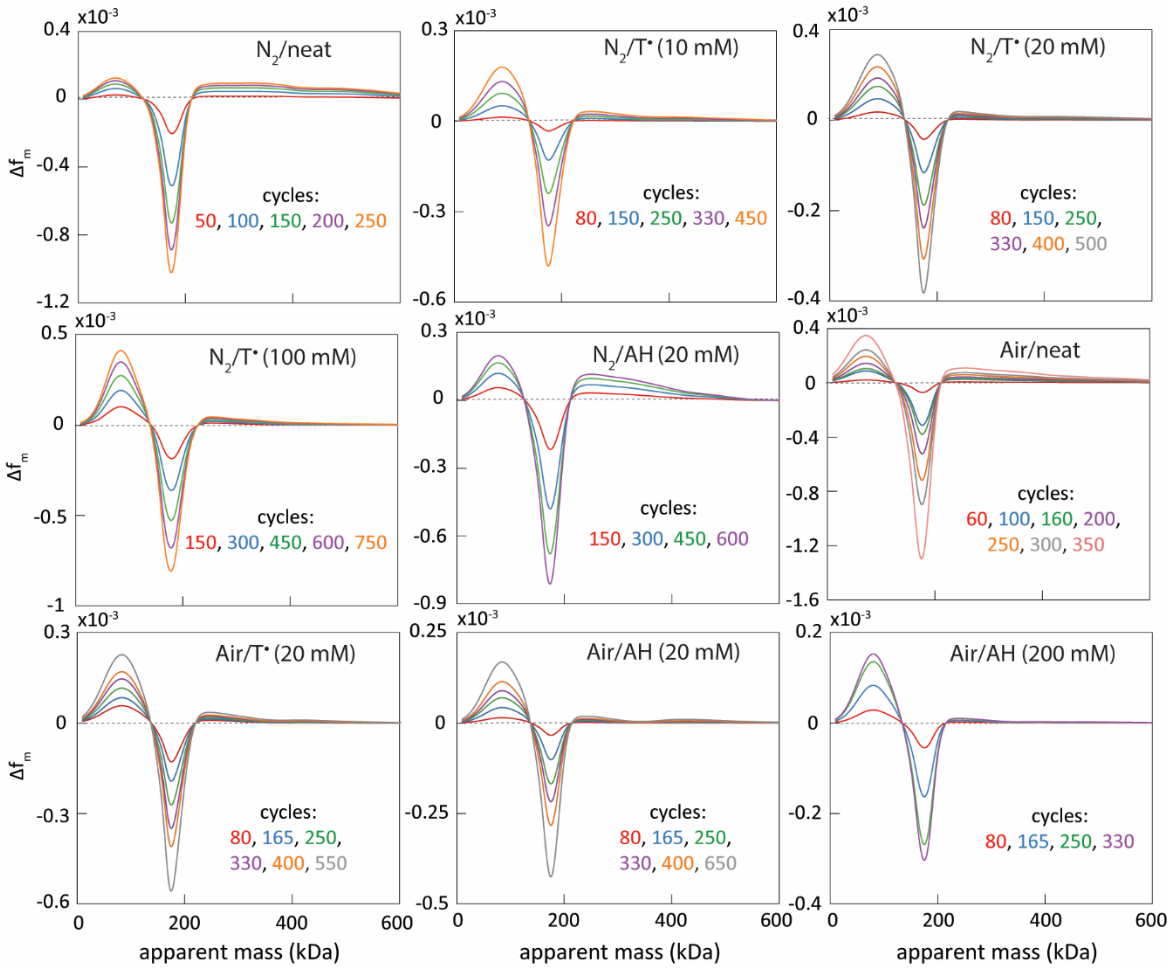

Supplementary Fig. 26 Simulated apparent differential mass distributions obtained by subtracting the mass distribution of the intact sample from those of sheared samples at each cycle shown. Δf_m is the different in the mass fraction of all chains in each 100 Da-wide bin f apparent chain masses.

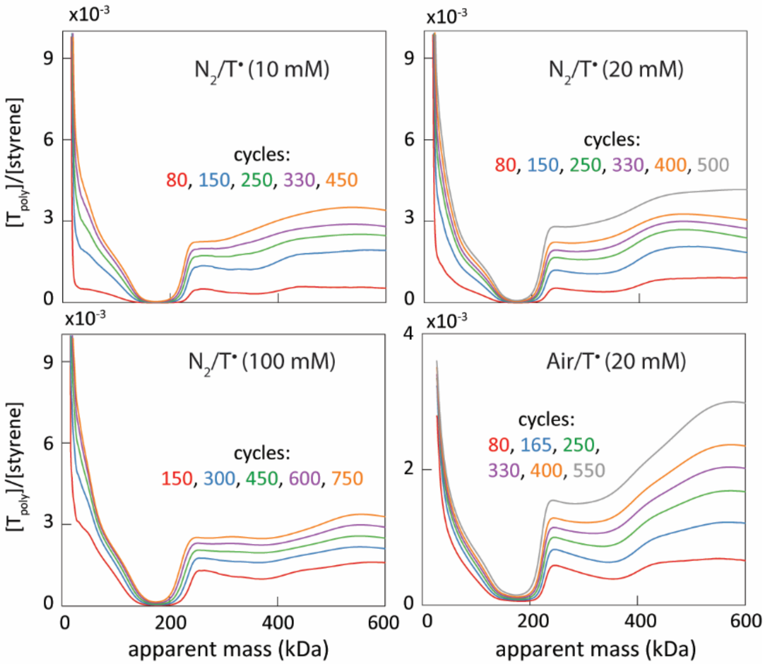

Supplementary Fig. 27 Simulated $[T_{poly}]/[\text{styrene}]$ ratios averaged over all chains with the apparent chain mass in each 100-Da wide bin that most closely match each experimental total $[T_{poly}]$ value.

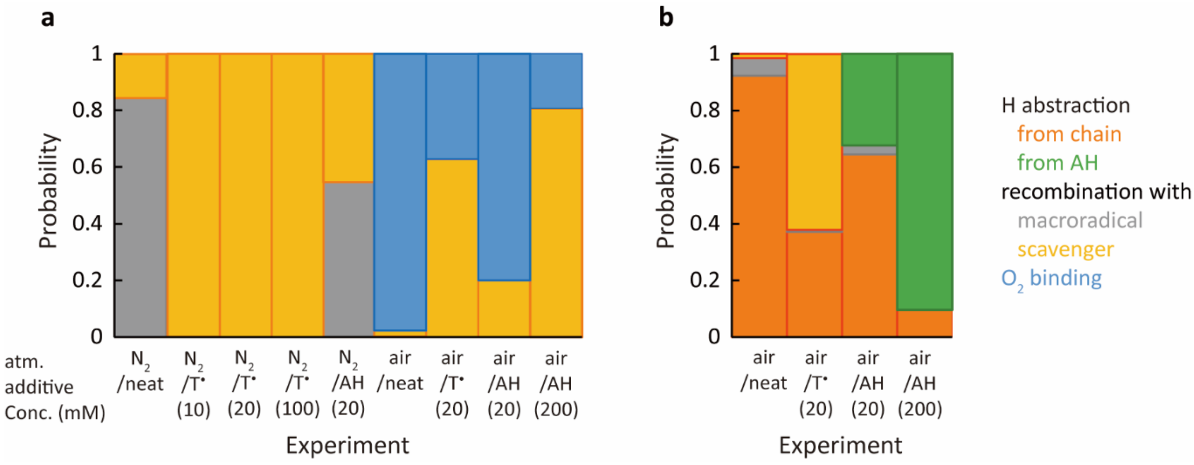

Supplementary Fig. 28 Reaction selectivities of sR• (a) and ROO• (b) macroradicals under different conditions. In sheared neat copolymer, the orange color signifies background rate of macroradical scavenging, presumably by an unknown small-molecule radical impurity.

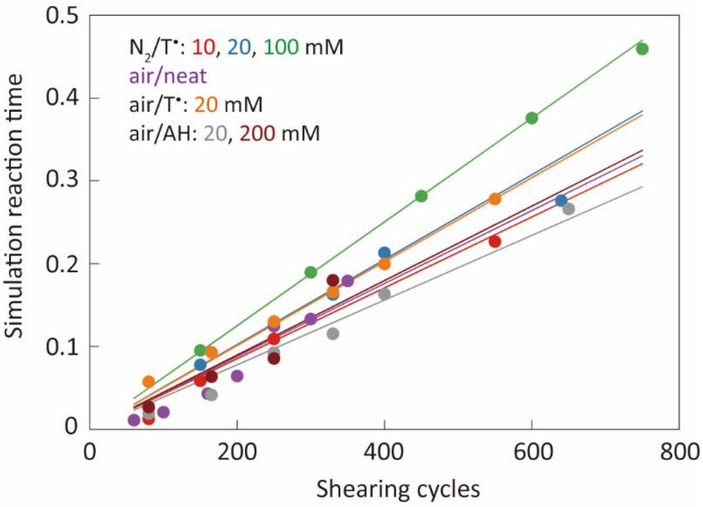

Supplementary Fig. 29 Correlation between simulation reaction time (unitless) and shearing cycles based on matching [T_{poly}]. Each dot corresponds to the simulation reaction time at which simulated [T_{poly}] matched that measured for the given cycle. Lines are LSFs using the robustfit algorithm with slopes of 4.274e-04, 5.126e-04, 6.263e-04, 4.403e-04, 5.065e-04, 3.903e-04, 4.493e-04 cycle^{-1}.

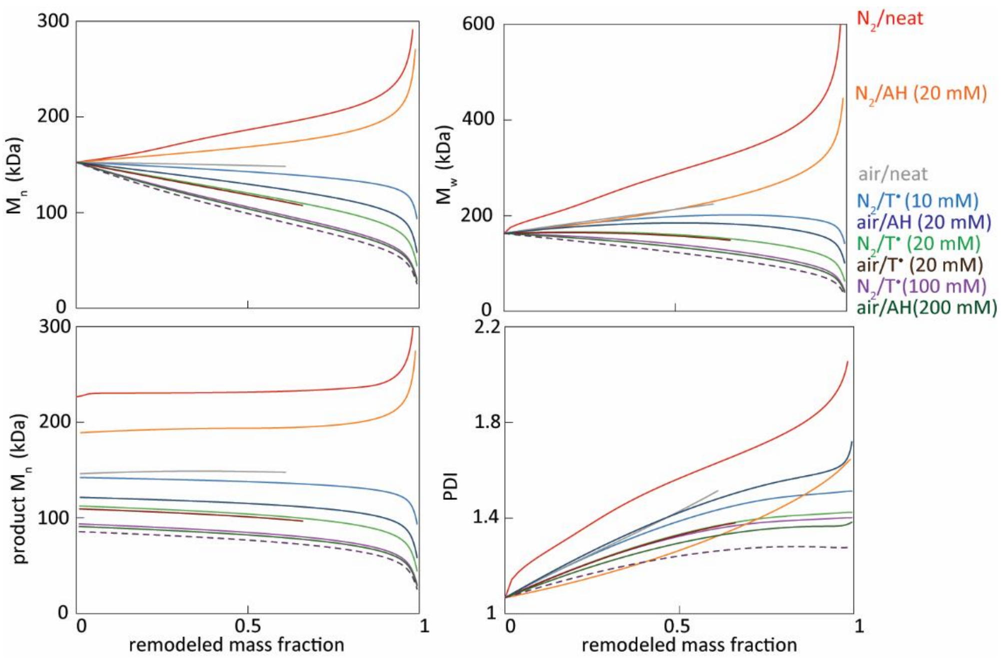

Supplementary Fig. 30 Number- and weight-average molar masses of the remodelling samples, M_n and M_w; the number-average molar mass of the product chains and the dispersity index of the sample as a function of the remodelled mass fraction. Dashed lines are values for a sample incapable of forming any C-C bonds (including by recombination) and is observed in sonicated dilute solutions of the copolymer.

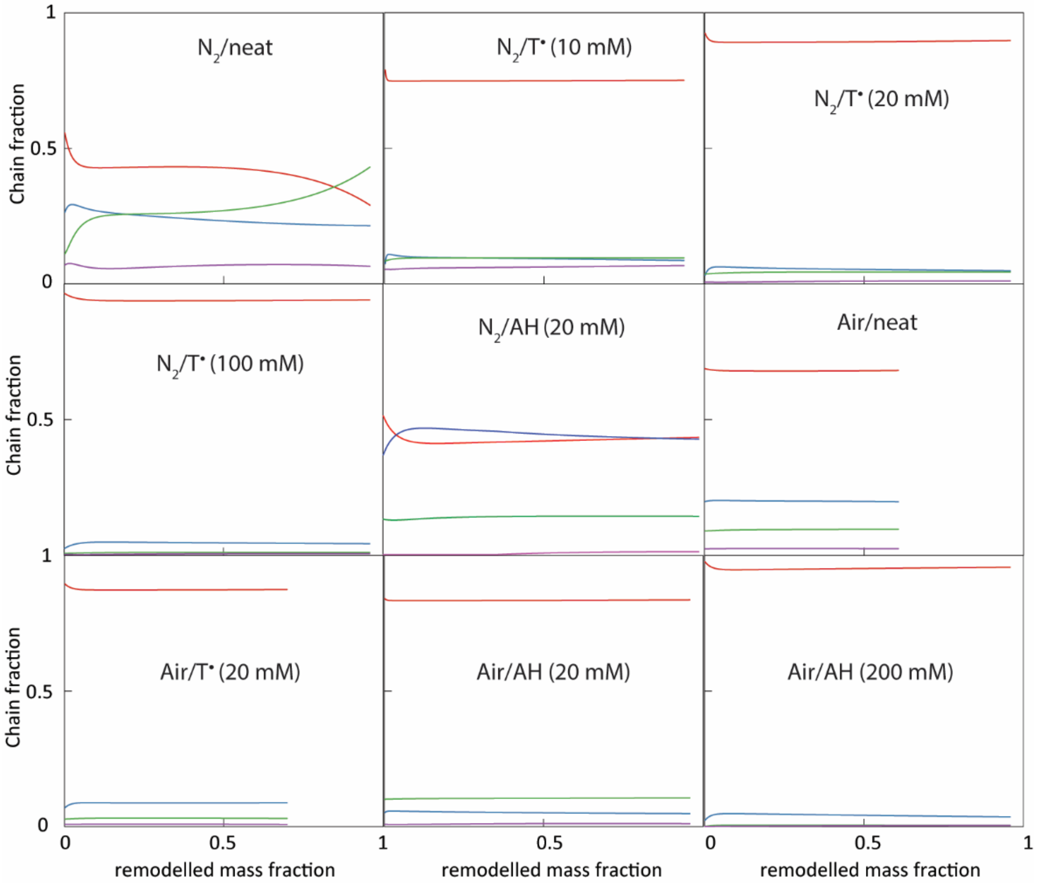

Supplementary Fig. 31 The fractions of chains of different topologies in the product mixtures as a function of the remodeled mass fraction: linear chains (LLM, red), 3-arm stars (blue), all other star chains (green), all other branched chains (magenta).

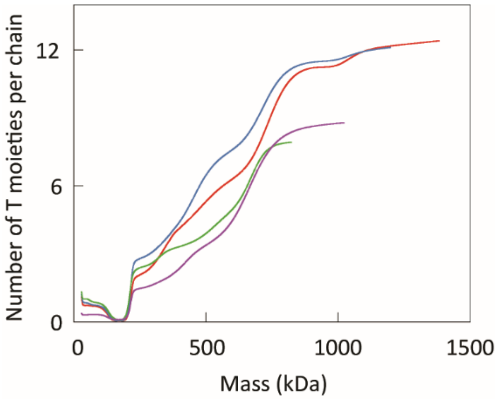

Supplementary Fig. 32 The average number of T moieties per chain of each mass at the end of each shearing experiment based on the contraction factors estimated from simulations: anaerobically sheared polymer containing T• at 10 (red), 20 (blue) and 100 (green) mM and aerobically sheared polymer containing 20 mM T• (magenta). Intact polymer dominates mass fractions at 160 -200 kDa, resulting in very low average T/chain ratios at these masses.

Supplementary Table 19 Estimates of the number of C-C bonds formed by radical addition per fractured C-C bonds, ν, based on the relative rate constants of addition to sp^2 carbons and abstraction of allylic H atoms using the calculated values for styrene/butadiene copolymer, ν^{ref}, k_a^{ref} and k_h^{ref}. Because styrene/butadiene copolymer has both primary and secondary sp^2-C atoms and both secondary and tertiary allylic H atoms, k_a^{ref} and k_h^{ref} are weighted as $k_j^{ref} = \frac{kT}{h}\left(c_1 e^{-\Delta H_1^{\ddagger}/RT} + c_2 e^{-\Delta H_2^{\ddagger}/RT}\right)$, where $\Delta H_i^{\ddagger}$ are the DFT-calculated enthalpies of activation for the two atom types and c_i is the correspondign concentration. The relevant data for the styrene/butadiene copolymer is summarized in Supplementary Table 4 ($\Delta H_a^{\ddagger}$), Supplementary Table 7 ($\Delta H_h^{\ddagger}$), Supplementary Table 9 (concentrations) All estimates assume that the termination rate is proportional to k_h, which accounts for both direct H atom abstraction and reaction diffusion leading to recombination.

	[sp^2-C], M	[H_{allyl}], M	k_a/k_a^{ref}	k_h/k_h^{ref}	Alternative estimate[(a)]
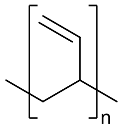	16	16	2.7	0.18	>10
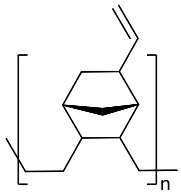	4.7	4.7	0.80	0.052	>5
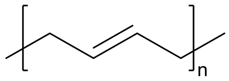	21	42	0.0004[(b)]	0.005[(c)]	~1

(a) using a modified equation for kinetic chain length of a radical polymerization without chain transfer: $\upsilon^{ref}\frac{k_a}{k_a^{ref}}\left(\frac{k_h^{ref}}{f(k_h+k_t)}\right)^{0.5}$, where f is the number of macroinitiators per chain fracture (f^{ref} = 1) and n^{ref} = 1.8, the measured number of C-C bonds formed per chain fracture in styrene/butadiene copolymer.

(b) Addition of sR• (fragmentation yields no aR•)

(c) the background rate constant of recombination with adventitious impurities estimated from microkinetic modeling of neat styrene/butadiene polymer

Appendix 1: numerical description of chain structures and concentrations

We described the composition of a remodeling copolymer by a set of pairs of vectors: a 1xn vector of integers describing chain size, and 1xm vector of single-precision floating point numbers describing concentrations of all derivatives of the chain (Supplementary Table 20). Here n is the number of segments of the chain and each integer is the number of repeat units, U, per segment. For example, a linear chain has a single segment, a 3-arm star chain has 3 segments and a chain with 2 trivalent branch points has 5 segments; their size vectors are 1x1, 1x3 and 1x5. The size of the concentration vector depended on the shearing conditions and the chain size as:

1. For anaerobic shearing of neat and AH-containing samples, it included (rows 1,5,9 in Supplementary Table 20 for examples):
    a. the concentration of the closed-shell chain;
    b. the concentrations of $aR^{\bullet}$ with the unpaired electron at each arm terminus (for chains with <6 termini), or the total concentration of terminal $aR^{\bullet}$, the fraction of these radicals with the unpaired electron at the shortest arm, the same value for the longest arm (for chains with 6+ termini);
    c. the concentration of terminal $sR^{\bullet}$ as a multiple of the total concentration of terminal $aR^{\bullet}$;
    d. concentration of macroradicals with unpaired electron at each branch point (these can only be $aR^{\bullet}$);
    e. concentration of macroradicals with the unpaired electron at any position along the chain (these can only be $sR^{\bullet}$, neglecting a small fraction of internal $aR^{\bullet}$ from fracture of branched chains at branch points).
2. For anaerobic shearing of T-containing samples (rows 2,6,10 in Supplementary Table 20 for examples) it included, in addition to a), b) and d) above, the number of T moieties, for both the closed-shell and $aR^{\bullet}$ chains, at:
    a. Each terminus (for chains with <6 termini) or at the shortest and longest termini (for chains with 6+ termini)
    b. Each branch point
    c. Elsewhere in the chain
3. For aerobically sheared neat and AH-containing samples (rows 3,7,12 in Supplementary Table 20 for examples) it included, in addition to a), b) and d) from point 1 above, the concentration of $ROO^{\bullet}$ with the OO moiety:
    a. At each terminus (for chains with <6 termini) or the total concentration of terminal $ROO^{\bullet}$ and the fraction of such radicals with the OO moiety at the shortest and the longest arms (for chains with 6+ termini)
    b. At each branch point
    c. Elsewhere in the chain.
4. For aerobically sheared T-containing samples (rows 4,8,12 in Supplementary Table 20 for examples) it included descriptors a), b) and d) from pt. 1, and all descriptors from pts. 2 and 3 above.

Chains with 13 or more segments were described by a size vector comprising the total chain size, effective contour length, *ecl*, (15)), the number of branching points, the valency of each branching point and a descriptor of chain topology (Supplementary Table 15). The concentration vector for such chains was:

1. For anaerobically sheared neat and AH-containing samples: (a) closed-shell chain, (b) terminal $aR^{\bullet}$, (c) terminal $sR^{\bullet}$, (d) radical at each branch point, (e) radical elsewhere in the chain.
2. For anaerobically sheared T-containing samples: in addition to (a), (b), (d) and (e), number of T moieties per chain, per terminal macroradical and at-branch-point macroradical: at all termini combined, at each branch point, elsewhere in the chain.
3. For aerobically sheared neat or AH-containing samples in addition to (a), (b), (d) and (e) from pt. 1, the total concentration of $tROO^{\bullet}$, $bROO^{\bullet}$ and $rROO^{\bullet}$ ($ROO^{\bullet}$ radicals with the OO moiety at any terminus, at each branch points and elsewhere in the chain).
4. For aerobically sheared T-containing samples), it contains components (a), (b), (d) and (e) from 1 and all components from 2 and 3.

During propagation of each time step, products of additions, fragmentations, and H-transfers were described by modified concentration vectors specific to each reaction. Such temporary concentration vectors could be longer or shorter than the standard vectors for a chain of the same topology, depending on the number of segments in the product chain. For example, the products of additions are macroradicals with unpaired electron only at a branch point and therefore their concentration vectors were truncated by eliminating elements describing closed-shell or terminal macroradicals. Likewise, fragmentations only generate terminal radicals (a small fraction of products that resulted from fragmentation at a branch point were assumed to be all $sR^{\bullet}$ and combined with internal radicals of the matching topology) and their temporary concentration vectors lacked elements for radicals at branch points. Conversely, for modeling T-containing samples, a subset of product chains with >5 segments were described with a concentration vector specifying the average number of T moieties at each terminus (rather than only the shortest and longest). An example is a chain generated by addition of a terminal 4-arm star macroradical to a 4-arm star that contains 3 branch points and 9 segments and would be initially described with a concentration vector containing 7 elements quantifying the number of T moieties at each of the 7 termini for remodeling of T-containing samples; eventually this part of the vector will be reduced to 2 elements by fitting to a linear dependence between the number of terminal T moieties and the size of the arm and scaling the resulting pair to preserve the total number of T moieties per chain. Likewise, the majority of product chains of fragmentation of a chain with 13 or more segments would have <13 segments but would temporarily be described by a coarse-grained size and concentration vectors.

These product-specific descriptions were standardized at the end of each propagation cycle to reduce the size of the variable describing the time-dependent composition and to allow consolidation of products of the current step with identical components carried over from preceding steps. Conversion from detailed to coarse-grained representations of speciation of chains with >5 segments and with >12 segments necessarily changed the total amount of radicals and/or polymer-bound T moieties by 1-3%. To maintain all mass balances, the concentrations of terminal macroradicals whose speciation was coarse-grained from every-terminus to shortest/longest termini were scaled so that their total concentration before and after coarse-graining didn’t change. For modeling of T-containing samples, a similar procedure was applied for the average number of terminal T moieties in the closed-shell and macroradicals to maintain the total amount of polymer-bound T before and after coarse-graining.

Supplementary Table 20 Examples of size and concentration vectors for different chain topologies and shearing conditions.

	Chain topology	conditions	Size vector	Concentration vector	notes
1		N_2/none or AH		$[\underbrace{2.62\times10^{-6}}_{closed-shell}, \underbrace{2.99\times10^{-7}}_{terminal\ aR^\bullet}, \underbrace{1.36}_{terminal\ \frac{sR^\bullet}{aR^\bullet}\ ratio}, \underbrace{1.63\times10^{-11}}_{radnomly\ localized\ sR^\bullet}]$	
2		$N_2/T^\bullet$	10	$[\underbrace{2.39\times10^{-6}}_{closed-shell}\ \underbrace{3.57\times10^{-7}}_{terminal\ aR^\bullet}\ \underbrace{\underbrace{\underbrace{0.21}_{at\ terminus}\ \underbrace{1.65\times10^{-3}}_{elsewhere}}_{closed\ shell}\ \underbrace{\underbrace{2.24\times10^{-2}}_{at\ terminus}\ \underbrace{3.56\times10^{-5}}_{elsewhere}}_{aR^\bullet}}_{T\ moieties\ per\ chain}]$	1
3		Air/none or AH		$\underbrace{2.43\times10^{-6}}_{closed-shell}\ \underbrace{3.07\times10^{-7}}_{terminal\ aR^\bullet}\ \underbrace{4.84\times10^{-7}}_{tROO^\bullet}\ \underbrace{1.42\times10^{-7}}_{rROO^\bullet}$	1-3
4		Air/$T^\bullet$		$\underbrace{1.36\times10^{-6}}_{closed-shell}\ \underbrace{3.37\times10^{-7}}_{terminal\ aR^\bullet}\ \underbrace{3.21\times10^{-7}}_{tROO^\bullet}\ \underbrace{1.59\times10^{-7}}_{rROO^\bullet}\ \underbrace{\underbrace{\underbrace{0.0415}_{at\ terminus}\ \underbrace{4.56\times10^{-3}}_{elsewhere}}_{closed\ shell}\ \underbrace{\underbrace{0.0233}_{at\ terminus}\ \underbrace{5.89\times10^{-4}}_{elsewhere}}_{aR^\bullet}}_{T\ moieties\ per\ chain}$	
5		N_2/none or AH		$[\underbrace{1.76\times10^{-8}}_{closed-shell}, \underbrace{4.09\times10^{-9}, 1.37\times10^{-9}, 7.24\times10^{-10}}_{terminal\ aR^\bullet}, \underbrace{1.22}_{terminal\ \frac{sR^\bullet}{aR^\bullet}\ ratio}, \underbrace{1.92\times10^{-8}}_{radical\ at\ branch\ pt}, \underbrace{4.34\times10^{-12}}_{radnomly\ localized\ sR^\bullet}]$	
6	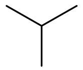	$N_2/T^\bullet$	[10 11 12]	$[\underbrace{1.37\times10^{-7}}_{closed-shell}\ \underbrace{2.12\times10^{-9}, 8.75\times10^{-10}, 4.31\times10^{-10}}_{terminal\ aR^\bullet}\ \underbrace{6.29\times10^{-9}}_{radical\ at\ branch\ pt}, \underbrace{\underbrace{\underbrace{0.62\ 0.47\ 0.44}_{at\ termini}\ \underbrace{0.98}_{at\ branch\ pt}\ \underbrace{5.60\times10^{-5}}_{elsewhere}}_{closed\ shell}\ \underbrace{\underbrace{0.11, 0.094, 0.087}_{at\ termini}\ \underbrace{0.47}_{at\ branch\ pt}\ \underbrace{0.0034}_{elsewhere}}_{aR^\bullet}}_{T\ moieties\ per\ chain}]$	6
7		Air/none or AH		$\underbrace{2.45\times10^{-7}}_{closed-shell}\ \underbrace{1.47\times10^{-9}, 3.57\times10^{-10}, 1.74\times10^{-10}}_{terminal\ aR^\bullet}\ \underbrace{4.86\times10^{-9}}_{radical\ at\ branch\ pt}, \underbrace{1.73\times10^{-9}, 5.43\times10^{-10}, 2.43\times10^{-10}}_{tROO^\bullet}\ \underbrace{3.76\times10^{-9}}_{bROO^\bullet}, \underbrace{7.46\times10^{-11}}_{rROO^\bullet}$	1-3
8		Air/$T^\bullet$		$\underbrace{3.05\times10^{-7}}_{closed-shell}\ \underbrace{8.71\times10^{-10}, 2.90\times10^{-10}, 1.02\times10^{-10}}_{terminal\ aR^\bullet}\ \underbrace{8.98\times10^{-10}}_{radical\ at\ branch\ pt}, \underbrace{7.77\times10^{-10}, 3.23\times10^{-10}, 1.07\times10^{-10}}_{tROO^\bullet}\ \underbrace{1.99\times10^{-9}}_{bROO^\bullet}, \underbrace{3.37\times10^{-12}}_{rROO^\bullet}$ $\underbrace{\underbrace{\underbrace{0.34\ 0.27\ 0.25}_{at\ terminus}\ \underbrace{0.52}_{at\ branch\ pt}\ \underbrace{4.86\times10^{-4}}_{elsewhere}}_{closed\ shell}\ \underbrace{\underbrace{0.14, 0.103, 0.075}_{at\ termini}\ \underbrace{0.26}_{at\ branch\ pt}\ \underbrace{0.0044}_{elsewhere}}_{aR^\bullet}}_{T\ moieties\ per\ chain}$	2-5
9		N_2/none or AH		$[\underbrace{4.12\times10^{-9}}_{closed-shell}, \underbrace{7.71\times10^{-10}, 0.31, 0.024}_{terminal\ aR^\bullet}, \underbrace{1.42}_{terminal\ \frac{sR^\bullet}{aR^\bullet}\ ratio}, \underbrace{1.27\times10^{-9}}_{radical\ at\ branch\ pt}, \underbrace{3.65\times10^{-12}}_{radnomly\ localized\ sR^\bullet}]$	7
10	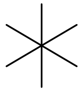	$N_2/T^\bullet$	[10 12 15 18 19 22]	$[\underbrace{5.46\times10^{-9}}_{closed-shell}\ \underbrace{4.64\times10^{-10}, 0.23, 0.11}_{terminal\ aR^\bullet}\ \underbrace{8.29\times10^{-10}}_{radical\ at\ branch\ pt}, \underbrace{\underbrace{\underbrace{0.64\ 0.29}_{at\ termini}\ \underbrace{0.98}_{at\ branch\ pt}\ \underbrace{1.51\times10^{-4}}_{elsewhere}}_{closed\ shell}\ \underbrace{\underbrace{0.14, 0.042}_{at\ termini}\ \underbrace{0.51}_{at\ branch\ pt}\ \underbrace{0.0022}_{elsewhere}}_{aR^\bullet}}_{T\ moieties\ per\ chain}$	7,8
11		Air/none or AH		$\underbrace{3.15\times10^{-8}}_{closed-shell}\ \underbrace{6.71\times10^{-10}, 0.19, 0.14}_{terminal\ aR^\bullet}\ \underbrace{4.86\times10^{-9}}_{radical\ at\ branch\ pt}, \underbrace{2.13\times10^{-9}, 0.20, 0.13}_{tROO^\bullet}\ \underbrace{4.34\times10^{-9}}_{bROO^\bullet}, \underbrace{1.04\times10^{-10}}_{rROO^\bullet}$	2,7
12		Air/$T^\bullet$		$[\underbrace{6.29\times10^{-9}}_{closed-shell}\ \underbrace{2.48\times10^{-10}, 0.20, 0.12}_{terminal\ aR^\bullet}\ \underbrace{6.09\times10^{-10}}_{radical\ at\ branch\ pt}, \underbrace{1.93\times10^{-9}, 0.22, 0.11}_{tROO^\bullet}\ \underbrace{2.75\times10^{-9}}_{bROO^\bullet}, \underbrace{4.94\times10^{-11}}_{rROO^\bullet}$ $\underbrace{\underbrace{\underbrace{0.32\ 0.18}_{at\ terminus}\ \underbrace{0.51}_{at\ branch\ pt}\ \underbrace{1.51\times10^{-4}}_{elsewhere}}_{closed\ shell}\ \underbrace{\underbrace{0.14, 0.042}_{at\ termini}\ \underbrace{0.31}_{at\ branch\ pt}\ \underbrace{0.0012}_{elsewhere}}_{aR^\bullet}}_{T\ moieties\ per\ chain}$	2,4,5, 7,8

1. [sR$^•$] is assumed to be negligible because it rapidly binds O_2 or T$^•$.
2. Only total amount of polymer-bound OOH moieties is tracked.
3. tROO$^•$: OO at a terminus; rROO$^•$: OO at a random location along the backbone (randomly-peroxidized chain); bROO$^•$: OO at a branch point
4. Number of terminal and randomly-distributed T moieties in tROO$^•$ and bROO$^•$ are assumed to be the same as in terminal aR$^•$ or radical-at-branch-pt aR$^•$, respectively, because both aR$^•$ and ROO$^•$ result from the same reaction (chain fracture or addition), followed by rapid O_2 binding in aerobic conditions.
5. Number of terminal and randomly-distributed T moieties in rROO$^•$ is assumed to be the same as in the closed-shell chain because rROO$^•$ is generated directly from closed-shell chains.
6. The numbers under the rubric "T moieties per chain/aR$^•$/at termini" is the total number of T moieties at all non-radical bearing termini; the concentration of aR with T at the radical-bearing terminus is very low (because such radicals are produced by the low-probability fracture of a branched chain at a branch point) and such chains are included in the "elsewhere" category.
7. The last 2 numbers in the "terminal aR$^•$" or "tROO$^•$" category are the fraction of all terminal aR$^•$ or terminal ROO$^•$ with the radical or OO moiety at the terminus of the shortest and longest arms. The fractions are assumed to be proportional to the length of each arm and the fractions of terminal aR or terminal ROO$^•$ with the radical (OO) at the terminus of an arm of an intermediate length is derived by linear interpolation.
8. The two numbers appearing under the rubric "T moieties per chain/closed-shell/at termini" and "T moieties per chain/aR$^•$/at termini" are for the shortest and longest arms with the number of T moieties at a terminus of an arm of either closed-shell or aR$^•$ chain assumed to be proportional to the length of the arm.

Supplementary references

1 Vilmin, F., Dussap, C. & Coste, N. Fast and robust method for the determination of microstructure and composition in butadiene, styrene-butadiene, and isoprene rubber by near-infrared spectroscopy. *Appl Spectrosc* **60**, 619-630, (2006).

2 Léger, L., Hervet, H., Charitat, T. & Koutsos, V. The stick–slip transition in highly entangled poly(styrene-butadiene) melts. *Advances in Colloid and Interface Science* **94**, 39-52, (2001).

3 Mori, S. & Barth, H. G. *Size Exclusion Chromatography*. (Springer-Verlag, 1999).

4 Striegel, A. M., Yau, W. W., Kirkland, J. J. & Bly, D. D. *Modern Size-Exclusion Liquid Chromatography*. 2 edn, (Wiley, 2009).

5 Voit, B. I. & Lederer, A. Hyperbranched and Highly Branched Polymer Architectures—Synthetic Strategies and Major Characterization Aspects. *Chemical Reviews* **109**, 5924-5973, (2009).

6 Georgantopoulos, C. K. *et al.* Modeling the spatial characteristics of extrusion flow instabilities for styrene-butadiene rubbers: Investigating the influence of molecular weight distribution, molecular architecture, and temperature. *Physics of Fluids* **33**, 093108, (2021).

7 Burfield, D. R. & Law, K. S. Determination of carbonyl groups in polymers by reaction with 2,4-dinitrophenylhydrazine. *Polymer* **20**, 620-626, (1979).

8 Arnold, A. R. & Staples, R. The determination of carbonyls as their 2,4-dinitrophenylhydrazine (DNPH) derivatives in peroxide-containing polymers. *Polymer* **33**, 1739-1741, (1992).

9 Liang, C., Li, J., Xia, M., Li, G. & Luo, Y. Performance and Kinetics Study of Self-Repairing Hydroxyl-Terminated Polybutadiene Binders Based on the Diels–Alder Reaction. *Polymers* **9**, (2017).

10 Cramer, C. J. *Essentials of Computational Chemistry*. 2nd ed. edn, (Wiley, 2004).

11 Kucharski, T. J. & Boulatov, R. The physical chemistry of mechanoresponsive polymers. *J. Mater. Chem.* **21**, 8237-8255, (2011).

12 Ochterski, J. W. Vibrational analysis in Gaussian. *help@gaussian.com*, (1999).

13 Leung, B. O., Reid, D. L., Armstrong, D. A. & Rauk, A. Entropies in solution from entropies in the gas phase. *J. Phys. Chem. A* **108**, 2720-2725, (2004).

14 Tian, Y. & Boulatov, R. Quantum-Chemical Validation of the Local Assumption of Chemomechanics for a Unimolecular Reaction. *ChemPhysChem* **13**, 2277-2281, (2012).

15 Akbulatov, S. *et al.* Experimentally realized mechanochemistry distinct from force-accelerated scission of loaded bonds. *Science (Washington, DC, U. S.)* **357**, 299-303, (2017).

16 Kucharski, T. J. & Boulatov, R. The physical chemistry of mechanoresponsive polymers. *J. Mater. Chem.* **21**, 8237-8255, (2011).

17 Tian, Y. & Boulatov, R. Comparison of the predictive performance of the Bell–Evans, Taylor-expansion and statistical-mechanics models of mechanochemistry. *Chem. Commun.* **49**, 4187-4189, (2013).

18 Akbulatov, S., Tian, Y. C. & Boulatov, R. Force-Reactivity Property of a Single Monomer Is Sufficient To Predict the Micromechanical Behavior of Its Polymer. *Journal of the American Chemical Society* **134**, 7620-7623, (2012).

19 Wang, J., Kouznetsova, T. B., Boulatov, R. & Craig, S. L. Mechanical gating of a mechanochemical reaction cascade. *Nat Commun* **7**, 13433, (2016).

20 Zhang, H. *et al.* Multi-modal mechanophores based on cinnamate dimers. *Nat Commun* **8**, 1147, (2017).

21 Akbulatov, S. & Boulatov, R. Experimental Polymer Mechanochemistry and its Interpretational Frameworks. *ChemPhysChem* **18**, 1422-1450, (2017).

22 Vandeputte, A. G. *et al.* Theoretical study of the thermodynamics and kinetics of hydrogen abstractions from hydrocarbons. *J. Phys. Chem. A* **111**, 11771-11786, (2007).

23 Sabbe, M. K., Reyniers, M. F., Van Speybroeck, V., Waroquier, M. & Marin, G. B. Carbon-centered radical addition and β-scission reactions: modeling of activation energies and pre-exponential factors. *ChemPhysChem* **9**, 124-140, (2008).

24 Knyazev, V. D. & Slagle, I. R. Thermochemistry of the R− O2 bond in alkyl and chloroalkyl peroxy radicals. *J. Phys. Chem. A* **102**, 1770-1778, (1998).

25 Lee, J. & Bozzelli, J. W. Thermochemical and kinetic analysis of the allyl radical with O2 reaction system. *Proceedings of the Combustion Institute* **30**, 1015-1022, (2005).

26 Van Krevelen, D. W. & Te Nijenhuis, K. *Properties of polymers: their correlation with chemical structure; their numerical estimation and prediction from additive group contributions*. (Elsevier, 2009).

27 Chen, C.-J. & Bozzelli, J. W. Thermochemical Property, Pathway and Kinetic Analysis on the Reactions of Allylic Isobutenyl Radical with O2: an Elementary Reaction Mechanism for Isobutene Oxidation. *The Journal of Physical Chemistry A* **104**, 9715-9732, (2000).

28 Bagryanskaya, E. G. & Marque, S. R. A. Scavenging of Organic C-Centered Radicals by Nitroxides. *Chemical Reviews* **114**, 5011-5056, (2014).

29 Maillard, B., Ingold, K. U. & Scaiano, J. C. Rate constants for the reactions of free radicals with oxygen in solution. *Journal of the American Chemical Society* **105**, 5095-5099, (1983).

30 Truhlar, D. G. Nearly encounter-controlled reactions: The equivalence of the steady-state and diffusional viewpoints. *J. Chem. Educ.* **62**, 104, (1985).

31 Leon-Carmona, J. R. & Galano, A. Is caffeine a good scavenger of oxygenated free radicals? *J. Phys. Chem. B* **115**, 4538-4546, (2011).

32 Pérez-González, A. & Galano, A. OH radical scavenging activity of edaravone: mechanism and kinetics. *J. Phys. Chem. B* **115**, 1306-1314, (2010).

33 Berry, R. S., Rice, S. A. & Ross, J. *Physical and chemical kinetics*. 2nd edn, Vol. 3 (Oxford University Press, 2002).

34 Matyjaszewski, K. & Xia, J. Atom Transfer Radical Polymerization. *Chemical Reviews* **101**, 2921-2990, (2001).

35 Kim, C., Beuve, J. S., Guilbert, S. & Bonfils, F. Study of chain branching in natural rubber using size-exclusion chromatography coupled with a multi-angle light scattering detector (SEC-MALS). *European Polymer Journal* **45**, 2249-2259, (2009).

36 Kratochvíl, P. & Netopilík, M. On the contraction factors of long-chain branched macromolecules. *European Polymer Journal* **51**, 177-181, (2014).
